# Decarboxylative-Allylation of Pyroglutamic Acid Derivatives: Stereocontrolled Access to Acyclic and Conformationally Restricted α,γ-Disubstituted γ-Amino Acids

**DOI:** 10.3390/molecules31122087

**Published:** 2026-06-14

**Authors:** Hugo Casas-Morales, Dácil Hernández, Mario Ordoñez, Alicia Boto, Ivan Romero-Estudillo

**Affiliations:** 1 Centro de Investigaciones Químicas-IICBA, Universidad Autónoma del Estado de Morelos, Av. Universidad 1001, Cuernavaca 62209, Mexico; hugo.casasmor@uaem.edu.mx (H.C.-M.); palacios@uaem.mx (M.O.); 2 Instituto de Productos Naturales y Agrobiología del CSIC, Avda. Astrofísico Fco. Sánchez, 3, 38206 La Laguna, Spain; dacil@ipna.csic.es

**Keywords:** γ-amino acids, diastereoselective alkylation, decarboxylation-allylation, conformationally constrained, stereoselective synthesis

## Abstract

The synthetic strategy relies on the highly diastereoselective alkylation at the C4 position of *L*-pyroglutamic acid derivatives, followed by a decarboxylation-allylation process that enables the incorporation of diverse substituents, including aromatic substituents, affording *trans*-3,5-disubstituted γ-lactams with excellent diastereiosmeric ratio (dr > 98:2). The resulting γ-lactams were efficiently transformed into a series of α,γ-disubstituted γ-amino acids through hydrogenation and acidic hydrolysis. Furthermore, cross-metathesis reactions with styrene and 1-decene enabled the introduction of structurally diverse lipophilic side chains, furnishing the corresponding γ-amino acids in good overall yields (71–77%) and high diastereoisomeric ratio from >98:2 to 92:8. In addition, *N*-allylation followed by ring-closing metathesis and hydrogenation provided access to a previously unexplored conformationally constrained γ-amino acid. Overall, seven α,γ-disubstituted γ-amino acids, including fluorinated and conformationally restricted derivatives, were synthesized from common intermediates with high stereocontrol. The developed methodology offers a versatile platform for the preparation of structurally diverse and underexplored γ-amino acid building blocks of potential interest in peptide synthesis, medicinal chemistry, and antimicrobial agent development.

## 1. Introduction

γ-Amino acid derivatives constitute important structural motifs in organic chemistry and have drawn much attention in drug discovery [1,2]. Among them, γ-aminobutyric acid (GABA), the simplest representative of this class, is a non-proteinogenic amino acid that functions as the principal inhibitory neurotransmitter in the mammalian central nervous system (CNS). Over the past decades, numerous chiral substituted γ-amino acids have been synthesized [3,4] and commercialized, such as (*S*)-pregabalin [5,6] (Lyrica^®^), (*S*)-vigabatrin [7,8] (Sabril^®^), (*R*)-baclofen [9,10] (Lioresal^®^) and gabapentin [11,12] (Neurotin^®^). Additionally, γ-amino acid derivatives represent attractive building blocks in the synthesis of peptides [13,14,15] and foldamers [16,17,18,19,20,21]. In this context, α,γ-disubstituted γ-amino acids represent a relatively underexplored class of amino acid derivatives [22,23,24,25,26].

These structural motifs are present in a variety of biologically active compounds, including inhibitors of neuronal GABA uptake [27,28] and of the human peptide transporter *hPepT1* [29], as well as derivatives of natural peptide tubulysin [30,31,32,33,34,35,36] which act as inhibitors of cancer cell proliferation. They are also incorporated into peptides exhibiting submicromolar affinities for human somatostatin receptors [37] and in short hybrid peptides displaying significant antimicrobial activity [38]. Furthermore, the conformationally constrained α,γ-disubstituted γ-amino acids have proven valuable in conformational studies, owing to their ability to modulate backbone geometry and secondary structure [39].

On the other hand, the introduction of a fluorine atom offers numerous advantages in medicinal chemistry. In many cases, fluorination improves physicochemical properties [40], enhances drug–receptor interactions [41], and restricts biotransformation, thereby reducing the formation of potentially toxic metabolites [42]. Therefore, the development of efficient synthetic methods for accessing fluorinated α,γ-disubstituted γ-amino acids remains highly desirable.

**Scheme 1:**
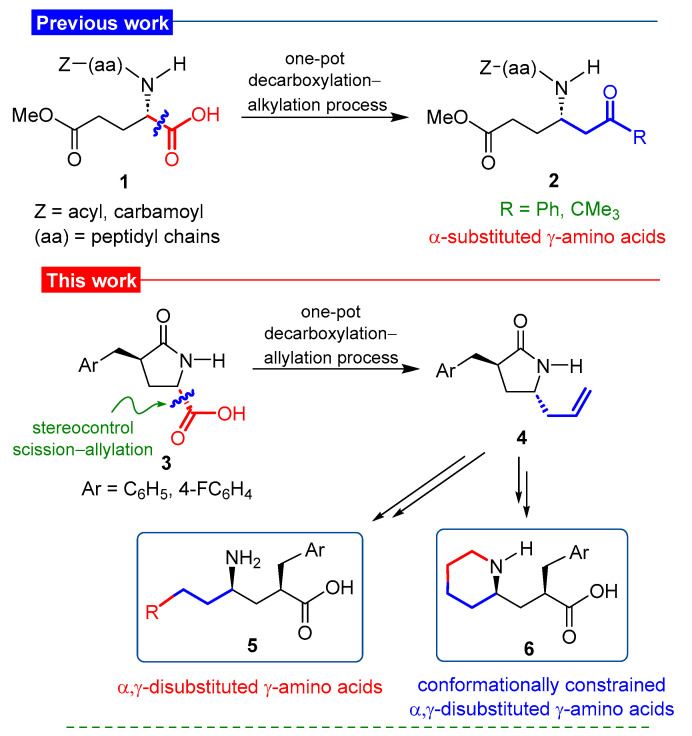
Previous work and current approach toward the synthesis of γ-amino acids.

In our previous work, we demonstrated that glutamic acid residues **1** are versatile intermediate “customizable units” for the synthesis of structurally diverse γ-amino acids embedded within peptide backbone **2**. This strategy relies on a one-pot decarboxylation-alkylation process [43,44,45,46] of orthogonally protected glutamic derivatives **1**, which affords the formation of hybrid α,γ-peptides **2** in good overall yields (Scheme 1) [47]. Despite these advances, many of the reported approaches for the synthesis of α,γ-disubstituted γ-amino acids remain limited by lengthy synthetic sequences, poor stereochemical control, and the requirement for expensive or scarcely available catalysts [22,23,24,25,26].

Herein, we report a novel and stereoselective approach for the synthesis of acyclic and conformationally restricted α,γ-disubstituted γ-amino acids **5** and **6** from α,γ-disubstituted γ-lactam **4,** easily obtained through a stereocontrolled decarboxylation-allylation process (Scheme 1). Unlike previous methodologies, this strategy enables the preparation of both acyclic and conformationally constrained γ-amino acids from a common intermediate derived from pyroglutamic acid **3**, a readily available chiral starting material. Additionally, the approach allows the introduction of side chains with diverse steric and electronic properties, including fluorinated substituents, while maintaining high levels of stereocontrol. These features make the methodology particularly attractive for the preparation of underexplored γ-amino acid building blocks with potential applications in peptide design and medicinal chemistry.

## 2. Results and Discussion

**Scheme 2:**
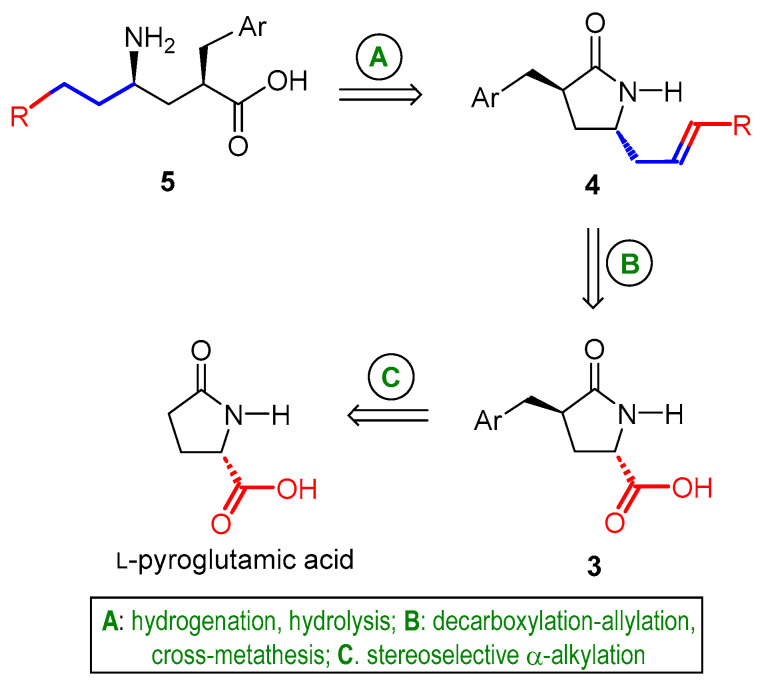
Retrosynthetic strategy for the synthesis of α,γ-disubstituted γ-amino acids **5**.

For the synthesis of our target α,γ-disubstituted γ-amino acid **5**, the α,γ-disubstituted γ-lactam **4** was proposed as a key intermediate, which can be obtained from 4-substituted pyroglutamic acid **3** through a stereocontrolled decarboxylation-allylation process followed by a cross-metathesis coupling. Additionally, 4-substituted pyroglutamic acid **3** can be prepared by stereoselective alkylation of appropriately protected *L*-pyroglutamic acid (Scheme 2).

**Scheme 3:**
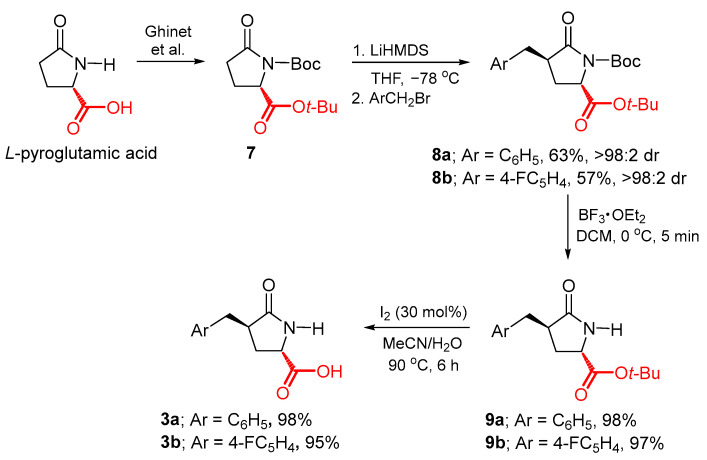
Synthesis of pyroglutamic acid derivatives **3a** and **b** [48].

Based on the proposed retrosynthetic analysis, we initiated the synthesis of our target molecules by focusing on the preparation of the chiral α,γ-disubstituted γ-lactam **4**. Following the procedure reported by Ghinet et al. [48], *L*-pyroglutamic acid was first converted into *tert*-butyl ester **16** and subsequently transformed into *N*-Boc *tert*-butyl pyroglutamate **7**, which reacted with lithium hexamethyldisilaside (LiHMDS) in THF at −78 °C, followed by the treatment with benzyl bromide, obtaining the (2*S*,4*R*)-*tert*-butyl *N*-Boc 4-benzylpyroglutamate **8a** in 63% yield and excellent diastereoselectivity (dr > 98:2) [49,50]. (Scheme 3).

Similarly, the alkylation reaction of **7** with 4-fluorobenzyl bromide produced the 4-substituted pyroglutamate **8b** in 57% yield and excellent diastereoselectivity (dr > 98:2) [51]. In the next step, *N*-Boc 4-substituted pyroglutamates **8a** and **b** were reacted with boron trifluoride ethyl etherate in dry dichloromethane at room temperature for 5 min [52], obtaining the corresponding (2*S*,4*R*)-*tert*-butyl 4-substituted pyroglutamates **9a** and **b** in 98 and 97% yield, respectively. Finally, treatment of **9a** and **b** with 30 mol% iodine in a MeCN/H_2_O mixture at 90 °C [53], provided the corresponding 4-substituted pyroglutamic acid derivatives **3a** and **b** in 98% and 95% yield, respectively (Scheme 3).

**Scheme 4:**
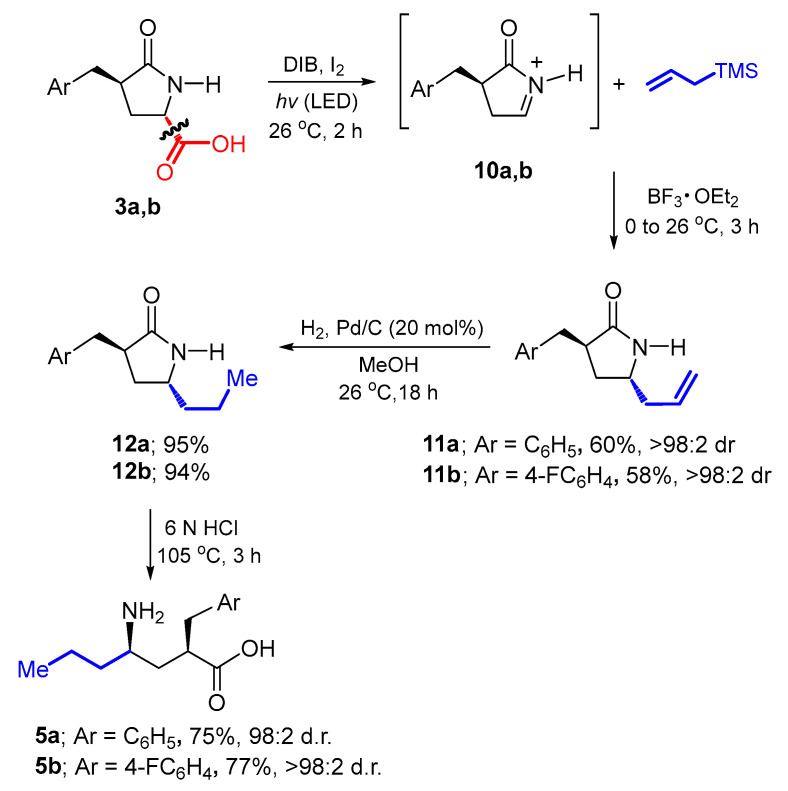
Synthesis of acyclic α,γ-disubstituted γ-amino acids **5a** and **b**.

Once 3-substituted pyroglutamic acid derivatives **3a** and **b** had been obtained, the decarboxylation-allylation process was investigated based on our previous work [54]. Initially, 4-substituted pyroglutamic acid derivatives **3a** and **b** were treated with (diacetoxyiodo)benzene (DIB) and iodine under white LED light irradiation (30 W) in dichloromethane, affording the corresponding acyliminium ion intermediates **10a** and **b**. Without further purification, these intermediates were subsequently reacted with allyltrimethylsilane and BF_3_·OEt_2_ at 0 to 26 °C, to furnish the corresponding 3,5-disubstituted γ-lactams **11a** and **b** in 60% and 58% yield, respectively, with a 98:2 diastereomeric ratio as determined by ^1^H NMR analysis of the crude reaction mixtures. The (*R*,*R*) configuration of **11a** and **b** was assigned based on NOE correlations between the protons at C2 and C4 (see the Supplementary Materials). Subsequently, catalytic hydrogenation of the double bond in 3,5-disubstituted γ-lactams **11a** and **b** under a hydrogen atmosphere in the presence of Pd/C (20 mol%) afforded the corresponding 3,5-disubstituted γ-lactams **12a** and **b** in 95% and 94% yield, respectively. Finally, acidic hydrolysis of **12a** and **b** with 6 N HCl at 105 °C furnished the desired α,γ-disubstituted γ-amino acids **5a** and **b** as their hydrochloride salts in 75% and 77%, respectively, with excellent diastereiosmeric ratios (dr = 98:2 and >98:2) (Scheme 4).

It is well known that short γ-peptides bearing an appropriate balance of lipophilic side chains and cationic residues display significant antimicrobial and antibiofilm properties [38,55,56]. In this context, the synthesis of γ-amino acids with lipophilic side chains represents an attractive strategy for the development of new antimicrobial agents.

**Scheme 5:**
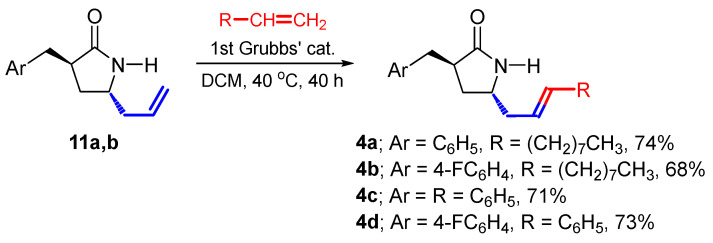
Cross-metathesis functionalization of 3,5-disubstituted γ-lactams **11a** and **b**.

With this in mind, we sought to evaluate the versatility of the allyl group in the 3,5-disubstituted γ-lactams **11a** and **b** for elongation of the lipophilic chain through a cross-coupling metathesis reaction. Thus, 3,5-disubstituted γ-lactam **11a** was subjected to cross-metathesis with 1-decene in the presence of first-generation Grubbs’ catalyst in dichloromethane at 40 °C, affording the corresponding 3,5-disubstituted γ-lactam **4a** in 74% yield. Under identical reaction conditions, precursor **11b** reacted with 1-decene to furnish the lactam **4b** in 68% yield. Additionally, the reaction of 3,5-disubstituted γ-lactams **11a** and **b** with styrene under the same cross-metathesis conditions afforded the corresponding 3,5-disubstituted γ-lactam **4c** and **d** in 71% and 73% yield, respectively (Scheme 5).

**Scheme 6:**
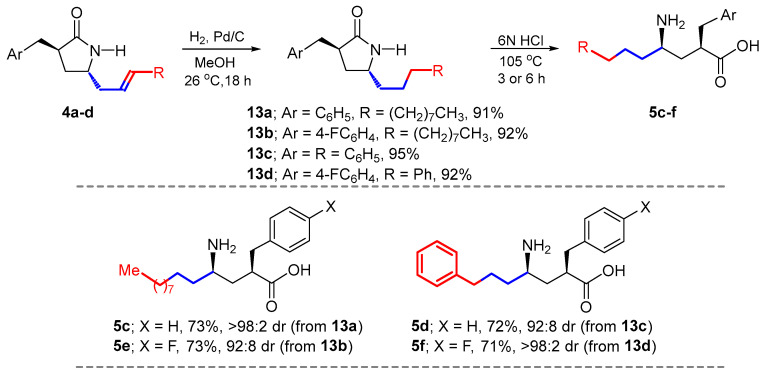
Synthesis of acyclic α,γ-disubstituted γ-amino acids **5c**–**f**.

Once lactams **4a**–**d** had been obtained, the next step involved the catalytic hydrogenation of the C=C bond under hydrogen atmosphere using Pd/C (20 mol%) in methanol at 26 °C, giving the corresponding 3,5-disubstituted γ-lactams **13a**–**d** in 91–95% yield. Finally, acidic hydrolysis of lactams **13a**–**d** with 6 N HCl at 105 °C furnished the target α,γ-disubstituted γ-amino acids **5c**–**f** as their hydrochloride salts in good yields 71–73% yield and with high diastereomeric ratios ranging from >98:2 to 92:8 (Scheme 6).

**Scheme 7:**
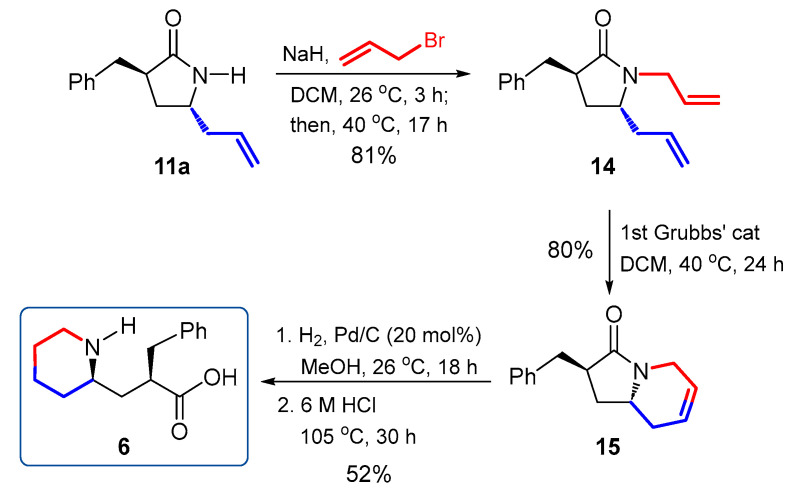
Synthesis of conformationally restricted α,γ-disubstituted γ-amino acid **6**.

In view of the significant role that conformationally restricted scaffolds play in drug discovery [57,58,59], this methodology could provide a versatile and strategically useful approach for the synthesis of structurally constrained γ-amino acids with potential pharmaceutical relevance. In this context, 3,5-disubstituted γ-lactam **11a** was treated with sodium hydride (NaH), followed by allyl bromide addition, to afford *N*-allyl 3,5-disubstituted γ-lactam **14** in 81% yield. Subsequent ring-closing metathesis (RCM) reaction [54] using first-generation Grubbs’ catalyst in dichloromethane at 40 °C, followed by the catalytic hydrogenation of the C=C bond under a hydrogen atmosphere using Pd/C (20 mol%) in methanol at 26 °C, furnished the bicyclic compound **15** in 80% yield. Finally, hydrolysis of the amide functionality in **15** with 6 N HCl afforded the conformationally constrained α,γ-disubstituted γ-amino acid **6** in 52% yield (Scheme 7).

Altogether, this methodology enables the generation of structurally diverse α,γ-disubstituted γ-amino acids from common precursors. Given the relevance of these scaffolds in peptide and medicinal chemistry, the present approach provides a valuable and modular platform for the preparation of acyclic and conformationally restricted α,γ-disubstituted γ-amino acids as building blocks with tunable structural and physicochemical properties.

## 3. Materials and Methods

All reagents and solvents were purchased from commercial suppliers and employed without additional purification. First-generation Grubbs’ catalyst was purchased from Sigma-Aldrich (Saint Louis, MO, USA) and handled under an argon atmosphere. Column chromatography was carried out using silica gel (Silica Flash 60^®^, 230–400 mesh, Quebec City, QC, Canada). Analytical thin-layer chromatography (TLC) was performed on silica gel 60 F_254_ plates (Merck, Boston, MA, USA), and spots were detected under UV irradiation or by staining with ninhydrin or potassium permanganate solutions. Melting points were measured on a Fisher-Johns melting point apparatus (Thermo Fisher Scientific, Waltham, MA, USA) and are reported without correction. Elemental compositions (C, H, and N) were determined using Thermo Scientific FLASH 2000 (Waltham, MA, USA), with experimental values differing by no more than ±0.4% from the theoretical values. NMR spectra (^1^H and ^13^C) were acquired in CDCl_3_ or CD_3_OD on Bruker (Billerica, MA, USA) AVANCE III HD (500.13 MHz) and JEOL (Peabody, MA, USA, 600 MHz) spectrometers. Tetramethylsilane (TMS) was used as the internal standard. Chemical shifts (δ) are reported in ppm, while coupling constants (*J*) are given in Hz.

*tert*-Butyl (*S*)-5-oxopyrrolidine-2-carboxylate (16) [48].

A solution of *L*-pyroglutamic acid (1.03 g, 8.0 mmol) in DCM (25 mL) was placed in a 100 mL round-bottom flask equipped with a magnetic stirring bar and cooled to 0 °C in an ice bath. Triethylamine (4.0 mL, 28.8 mmol) was added dropwise under stirring. Subsequently, ZnBr_2_ (4.50 g, 20.0 mmol) was added portionwise, followed by the slow addition of *tert*-butyl bromide (2.7 mL, 24.0 mmol). The resulting reaction mixture was allowed to warm to 40 °C and stirred for 15 h. After this period, the solvent was removed under reduced pressure. The resulting crude solid was washed with EtOAc (3 × 15 mL). The combined organic layers were washed with H_2_O (9 × 25 mL), dried over anhydrous Na_2_SO_4_, filtered, and concentrated under reduced pressure. The residue was dried under high vacuum without further purification to afford compound **16** (1.00 g, 67%) as a white crystalline solid; m.p. 102–103 °C; [α]_D_^20^ = +7.0° (*c* 1.0, MeOH).

Di-*tert*-butyl (*S*)-5-oxopyrrolidine-1,2-dicarboxylate (**7**) [50].

A solution of *tert*-butyl ester **16** (0.69 g, 3.7 mmol) in MeCN (5 mL) was placed in a 50 mL round-bottom flask equipped with a magnetic stirring bar. Di-*tert*-butyl dicarbonate (1.07 g, 4.9 mmol) and DMAP (0.37 g, 3.0 mmol) were added. The reaction mixture was stirred at 26 °C for 48 h. After this period, the solvent was removed under reduced pressure. The resulting crude product was purified by flash column chromatography using DCM:MeOH (95:05) as the eluent to afford compound **17** (0.76 g, 72%) as a white crystalline solid; m.p. 51–53 °C; [α]_D_^20^ = −36.9° (*c* 1.0, CHCl_3_).

Di-*tert*-butyl (2*S*)-4-benzyl-5-oxopyrrolidine-1,2-dicarboxylate (**8a**) [49,50].

A solution of di-*tert*-butyl (*S*)-5-oxopyrrolidine-1,2-dicarboxylate **17** (1.00 g, 3.5 mmol) in dry THF (50 mL) was placed in a 100 mL round-bottom flask equipped with a magnetic stirring bar and cooled to −70 °C. Lithium bis(trimethylsilyl)amide (LiHMDS) (3.7 mL, 3.7 mmol) was added dropwise and stirred for 1 h. After this period, benzyl bromide (0.4 mL, 3.7 mmol) was then added slowly, and the reaction mixture was stirred at the same temperature for 2 h. After completion, the solvent was removed under reduced pressure. The crude product was treated with 10% solution of NH_4_Cl (3 mL) and brine (10 mL). The resulting mixture was extracted with DCM (3 × 15 mL). The combined organic layers were dried over anhydrous Na_2_SO_4_, filtered, and concentrated under reduced pressure. The resulting crude product was purified by flash column chromatography using Hexane:EtOAc (80:20) as eluent to afford compound **8a** (0.83 g, 63%) as a white crystalline solid; m.p. 124–125 °C; [α]_D_^20^ = −49.1° (*c* 0.9, CHCl_3_).

Di-*tert*-butyl (2*S*)-4-(4-fluorobenzyl)-5-oxopyrrolidine-1,2-dicarboxylate (**8b**) [51].

A solution of di-*tert*-butyl (*S*)-5-oxopyrrolidine-1,2-dicarboxylate **17** (1.00 g, 3.5 mmol) in dry THF (50 mL) was placed in a 100 mL round-bottom flask equipped with a magnetic stirring bar and cooled to −70 °C. LiHMDS (3.7 mL, 3.7 mmol) was added dropwise and stirred for 1 h. After this time, 4-fluorobenzyl bromide (0.5 mL, 3.7 mmol) was then added slowly, and the reaction mixture was stirred at the same temperature for 2 h. After this period, the solvent was removed under reduced pressure. The crude product was treated with 10% solution of NH_4_Cl (3 mL) and brine (10 mL). The resulting mixture was extracted with DCM (3 × 15 mL). The combined organic layers were dried over anhydrous Na_2_SO_4_, filtered, and concentrated under reduced pressure. The resulting crude product was purified by flash column chromatography using Hexane:EtOAc (80:20) as the eluent to afford compound **8b** (0.78 g, 57%) as a white crystalline solid; m.p. 101–102 °C; [α]_D_^20^ = −48.0° (*c* 0.9, CHCl_3_).

*tert*-Butyl (2*S*,4*R*)-4-benzyl-5-oxopyrrolidine-2-carboxylate (**9a**).

A solution of di-*tert*-butyl (2*S*)-4-benzyl-5-oxopyrrolidine-1,2-dicarboxylate **8a** (1.17 g, 3.1 mmol) in dry DCM (1.5 mL) was placed in a 25 mL round-bottom flask equipped with a magnetic stirring bar and cooled to 0 °C in an ice bath. BF_3_·OEt_2_ (0.4 mL, 3.1 mmol) was added dropwise and stirred for 5 min. After this period, the reaction mixture was treated with a saturated solution of NaHCO_3_ (1.5 mL) and stirred for 15 min. The resulting mixture was extracted with DCM (3 × 15 mL). The combined organic layers were dried over anhydrous Na_2_SO_4_, filtered, and concentrated under reduced pressure. The resulting crude product was purified by flash column chromatography using Hexane:EtOAc (50:50) as eluent to afford compound **9a** (0.84 g, 98%) as a white crystalline solid; m.p. 67–69 °C; [α]_D_^20^ = −9.6° (*c* 1.0, CHCl_3_). Anal. Calcd for C_16_H_21_NO_3_: C, 69.79; H, 7.69; N, 5.09. Found: C, 69.86; H, 7.71; N, 5.07. ^1^H NMR (500 MHz, CDCl_3_) δ 1.43 (s, 9H, CCH_3_), 2.13 (ddd, *J* = 13.4, 8.9, 8.0 Hz, 1H, 3-H_a_), 2.22 (ddd, *J* = 13.4, 8.8, 3.8 Hz, 1H, 3-H_b_), 2.70 (dd *J* = 13.6, 9.3 Hz, 1H, CH_a_Ph), 2.78 (tdd, *J* = 8.7, 8.7, 8.0, 4.0 Hz, 1H, 4-H), 3.18 (dd, *J* = 13.7, 3.9 Hz, 1H, CH_b_Ph), 3.88 (ddd, *J* = 9.0, 3.8, 0.9 Hz, 1H, CHN), 6.10 (br s, 1H, NH), 7.19–7.25 (m, 3H, *o*-Ar + *p*-Ar), 7.30 (dd, *J* = 7.5, 6.9 Hz, 2H, *m*-Ar). ^13^C NMR (125.7 MHz, CDCl_3_) δ 28.1 (3 × CH_3_, CCH_3_), 30.5 (CH_2_, 3-C), 36.7 (CH_2_, CH_2_Ph), 41.6 (CH, 4-C), 54.4 (CH, 2-C), 82.5 (C, CCH_3_), 126.7 (CH, CH*_p_*_-arom_), 128.7 (2 × CH, *o*-Ar_),_ 129.2 (2 × CH, *m*-Ar_),_ 138.9 (C, Ar), 171.3 (C=O), 179.0 (C=O).

*tert*-Butyl (2*S*,4*R*)-4-(4-fluorobenzyl)-5-oxopyrrolidine-2-carboxylate (**9b**).

A solution of di-*tert*-butyl (2*S*)-4-(4-fluorobenzyl)-5-oxopyrrolidine-1,2-dicarboxylate **8b** (1.03 g, 2.6 mmol) in dry DCM (1.5 mL) was placed in a 25 mL round-bottom flask equipped with a magnetic stirring bar and cooled to 0 °C in an ice bath. BF_3_·OEt_2_ (0.3 mL, 2.6 mmol) was added dropwise and stirred for 5 min. After this period, the reaction mixture was treated with a saturated solution of NaHCO_3_ (1.5 mL) and stirred for 15 min. The resulting mixture was extracted with DCM (3 × 15 mL). The combined organic layers were dried over anhydrous Na_2_SO_4_, filtered, and concentrated under reduced pressure. The resulting crude product was purified by flash column chromatography using Hexane:EtOAc (50:50) as eluent to afford compound **9b** (0.75 g, 97%) as a white solid; m.p. 79–81 °C; [α]_D_^20^ = −7.1° (*c* 1.0, CHCl_3_). Anal. Calcd for C_16_H_20_FNO_3_: C, 65.51; H, 6.87; N, 4.78. Found: C, 65.66; H, 6.89; N, 4.76.). ^1^H NMR (500 MHz, CDCl_3_) δ 1.44 (s, 9H, CCH_3_), 2.06–2.14 (m, 1H, 3-H_a_), 2.18–2.26 (m, 1H, 3-H_a_), 2.68–2.77 (m, 2H, CH_a_Ph + 4-H), 3.08–3.13 (m, 1H, CH_b_Ph), 3.86 (ddd, *J* = 9.0, 3.7, 0.8 Hz, 1H, CHN), 6.06 (br s, 1H, NH), 6.98 (ddd, *J* = 8.9, 8.8, 2.2 Hz, 2H, *m*-Ar), 7.17 (ddd, *J* = 8.8, 5.5, 2.9 Hz, 2H, *o*-Ar). ^13^C NMR (125.7 MHz, CDCl_3_) δ 28.1 (3 × CH_3_, CCH_3_), 30.3 (CH_2_, 3-C), 35.8 (CH_2,_ CH_2_Ph), 41.5 (CH, 4-C), 54.3 (CH, 2-C), 82.6 (C, CCH_3_), 115.5 (2 × CH, d, *J* = 21.7 Hz, *m*-Ar), 130.7 (2 × CH, *o*-Ar), 134.4 (C, Ar), 161.8 (C, d, *J* = 245 Hz, CF), 171.2 (C=O), 178.7 (C=O).

(2*S*,4*R*)-4-Benzyl-5-oxopyrrolidine-2-carboxylic acid (**3a**).

A solution of *tert*-butyl (2*S*,4*R*)-4-benzyl-5-oxopyrrolidine-2-carboxylate **9a** (0.83 g, 3.0 mmol) in MeCN (8.0 mL) was placed in a 25 mL round-bottom flask equipped with a magnetic stirring bar. The resulting mixture was treated with H_2_O (40 μL) and iodine (0.23 g, 0.9 mmol). The resulting reaction mixture was allowed to warm to 90 °C and stirred for 6 h. After this period, DCM (5 mL) was added and washed with 10% solution of Na_2_S_2_O_3_ (3 × 10 mL). The combined organic layers were dried over anhydrous Na_2_SO_4_, filtered, and concentrated under reduced pressure. The resulting crude product was purified by flash column chromatography using DCM:MeOH (90:10) as the eluent to afford compound **3a** (0.65 g, 98%) as a white solid; m.p. 146–148 °C; [α]_D_^20^ = −5.4° (*c* 1.0, MeOH). Anal. Calcd for C_12_H_13_NO_3_: C, 65.74; H, 5.98; N, 6.39. Found: C, 65.57; H, 6.00; N, 6.41. ^1^H NMR (500 MHz, CD_3_OD) δ 2.10–2.22 (m, 2H, 3-H_2_), 2.67 (dd, *J* = 13.6, 9.2 Hz, 1H, CH_a_Ph), 2.77 (tdd, *J* = 8.9, 8.7, 8.7, 4.1 Hz, 1H, 4-H), 3.09 (dd, *J* = 13.6, 4.1 Hz, 1H, CH_b_Ph), 3.96 (dd, *J* = 8.7, 4.0 Hz, 1H, 2-H), 7.18–7.24 (m, 3H, *o*-Ar + *p*-Ar)_,_ 7.28 (dd, *J* = 7.5, 6.9 Hz, 2H, *m*-Ar). ^13^C NMR (125.7 MHz, CD_3_OD) δ 31.9 (CH_2_, 3-C), 37.3 (CH_2_, CH_2_Ph), 43.3 (CH, 4-C), 55.4 (CH, 2-C), 127.5 (CH, *p*-Ar), 129.6 (2 × CH, *o*-Ar), 130.1 (2 × CH, *m*-Ar), 140.2 (C, Ar), 176.5 (C=O), 181.7 (C=O).

(2*S*,4*R*)-4-(4-Fluorobenzyl)-5-oxopyrrolidine-2-carboxylic acid (**3b**).

A solution of *tert*-butyl (2*S*,4*R*)-4-(4-fluorobenzyl)-5-oxopyrrolidine-2-carboxylate **9b** (0.76 g, 2.6 mmol) in MeCN (8.0 mL) was placed in a 25 mL round-bottom flask equipped with a magnetic stirring bar. The resulting mixture was treated with H_2_O (40 μL) and iodine (0.20 g, 0.8 mmol). The resulting reaction mixture was allowed to warm to 90 °C and stirred for 6 h. After this period, DCM (5 mL) was added and washed with 10% solution of Na_2_S_2_O_3_ (3 × 10 mL). The combined organic layers were dried over anhydrous Na_2_SO_4_, filtered, and concentrated under reduced pressure. The resulting crude product was purified by flash column chromatography using DCM:MeOH (90:10) as the eluent to afford compound **3b** (0.58 g, 95%) as a white solid; m.p. 129–131 °C; [α]_D_^20^ = −5.0° (*c* 1.0, MeOH). Anal. Calcd for C_12_H_12_FNO_3_; C, 60.76; H, 5.10; N, 5.90. Found: C, 60.74; H, 5.11; N, 5.88. ^1^H NMR (500 MHz, CD_3_OD) δ 2.15–2.19 (m, 2H, 3-H_2_), 2.69 (dd, *J* = 13.6, 9.0 Hz, 1H, CH_a_Ph), 2.76 (tdd, *J* = 8.7, 8.6, 8.6, 4.1 Hz, 1H, 4-H), 3.07 (dd, *J* = 13.6, 4.1 Hz, 1H, CH_b_Ph), 4.00 (dd, *J* = 6.8, 5.7 Hz, 1H, 2-H), 7.01 (ddd, *J* = 8.9, 8.8, 3.1 Hz, 2H, *m*-Ar), 7.23 (ddd, *J* = 8.8, 5.4, 3.1 Hz, 2H, *o*-Ar). ^13^C NMR (125.7 MHz, CD_3_OD) δ 31.6 (CH_2_, 3-C), 36.4 (CH_2_, CH_2_Ph), 43.1 (CH, 4-C), 55.0 (CH, 2-C), 116.1 (2 × CH, d, *J* = 22.6 Hz, *m*-Ar), 131.8 (2 × CH, d, *J* = 7.5 Hz, *o*-Ar), 136.1 (C, Ar), 163.1 (C, d, *J* = 242.6 Hz, CF), 175.8 (C=O), 181.5 (C=O).

General procedure A: Decarboxylation-allylation.

A solution of the corresponding carboxylic acid **3a** or **3b** (1.0 equiv) in dry DCM was placed in a 25 mL round-bottom flask equipped with a magnetic stirring bar. The reaction mixture was treated with (diacetoxyiodo)benzene (DIB) (0.7 equiv) and iodine (0.2 equiv). The reaction mixture was stirred at 26 °C for 3 h under irradiation with white LED light (30 W). After this period, the reaction mixture was cooled to 0 °C in an ice bath. Allyltrimethylsilane (2.5 equiv) was added dropwise, followed by the treatment of BF_3_·OEt_2_ (1 equiv). The reaction mixture was allowed to warm to room temperature and stirred for 3 h. The resulting mixture was then washed with 10% aqueous Na_2_S_2_O_3_ (3 × 10 mL). The combined organic layers were dried over anhydrous Na_2_SO_4_, filtered, and concentrated under reduced pressure. The crude product was purified by flash column chromatography to afford the corresponding product.

(3*R*,5*R*)-5-Allyl-3-benzylpyrrolidin-2-one (**11a**).

According to General procedure A: A solution of the acid **3a** (0.11 g, 0.5 mmol) in DCM (10.0 mL) was treated with DIB (0.11 g, 0.35 mmol), iodine (0.025 g, 0.1 mmol), allyltrimethylsilane (0.2 mL, 1.25 mmol) and BF_3_·OEt_2_ (0.1 mL, 0.5 mmol). The crude product was purified by flash column chromatography using EtOAc:Hexane (70:30) as eluent to afford lactam **11a** (0.065 g, 60%) as a white solid; m.p. 92–93 °C; [α]_D_^20^ = −28.4° (*c* 1.0, CHCl_3_). Anal. Calcd for C_14_H_17_NO; C, 78.10; H, 7.96; N, 6.51. Found: C, 77.97; H, 7.93; N, 6.52. ^1^H NMR (500 MHz, CDCl_3_) δ 1.83 (ddd, *J* = 13.2, 8.9, 4.2 Hz, 1H, 4-H_a_), 1.95 (ddd, *J* = 13.2, 7.9, 7.0 Hz, 1H, 4-H_b_), 2.10–2.25 (m, 2H, 1′-H_2_), 2.66 (dd, *J* = 13.6, 9.8 Hz, 1H, CH_a_Ph), 2.72–2.79 (m, 1H, 3-H), 3.17 (dd, *J* = 13.6, 4.0 Hz, 1H, CH_b_Ph), 3.45–3.51 (m, 1H, 5-H), 5.05–5.11 (m, 2H, 3′-H_2_), 5.66–5.70 (m, 1H, 2′-H), 6.13 (br s, 1H, NH), 7.20–7.23 (m, 1H, *p*-Ar),7.21 (d, *J* = 6.6 Hz, 2H, *o*-Ar), 7.29 (ddd, *J* = 7.3, 7.0, 1.6 Hz, 2H, *m*-Ar). ^13^C NMR (125.7 MHz, CDCl_3_) δ 32.0 (CH_2_, 4-C), 37.0 (CH_2_, CH_2_Ph), 41.0 (CH_2_, 1′-C), 42.3 (CH, 3-C), 51.7 (CH, 5-C), 118.6 (CH_2_, 3′-C), 126.5 (CH, *p*-Ar), 128.6 (2 × CH, *o*-Ar), 129.2 (2 × CH, *m*-Ar), 133.7 (CH, 2′-C), 139.4 (C, Ar), 179.1 (C=O).

(3*R*,5*R*)-5-Allyl-3-(4-fluorobenzyl)pyrrolidin-2-one (**11b**).

According to General procedure A: A solution of the acid **3b** (0.047 g, 0.2 mmol) in DCM (4.0 mL) was treated with DIB (0.045 g, 0.14 mmol), iodine (0.010 g, 0.04 mmol), allyltrimethylsilane (0.08 mL, 0.5 mmol) and BF_3_·OEt_2_ (0.02 mL, 0.2 mmol). The crude product was purified by flash column chromatography using EtOAc:Hexane (70:30) as eluent to afford lactam **11b** (0.027 g, 58%) as a colorless oil; [α]_D_^20^ = −23.2° (*c* 1.0, CHCl_3_). Anal. Calcd for C_14_H_16_FNO; C, 72.08; H, 6.91; N, 6.00. Found: C, 72.15; H, 6.89; N, 5.99. ^1^H NMR (500 MHz, CDCl_3_) δ 1.83 (ddd, *J* = 13.0, 8.7, 4.0 Hz, 1H, 4-H_a_), 1.97 (ddd, *J* = 13.1, 8.0, 7.0 Hz, 1H, 4-H_b_), 2.10–2.25 (m, 2H, CH_2_CH), 2.68–2.73 (m, 2H, CH_a_Ph + 3-H), 3.10 (dd, *J* = 13.1, 3.5 Hz, 1H, CH_b_Ph), 3.46 (m, 1H, 5-H), 5.08–5.14 (m, 2H, 3′-H_2_), 5.64–5.74 (m, 1H, 2′-H), 5.98 (br, 1H, NH), 6.97 (ddd *J* = 8.7, 8.7, 3.1 Hz, 2H, *m*-Ar), 7.16 (ddd, *J* = 8.8, 5.4, 3.1 Hz, 2H, *o*-Ar), ^13^C NMR (125.7 MHz, CDCl_3_) δ 31.8 (CH_2_, 4-C), 36.1 (CH_2_, CH_2_Ph), 41.1 (**C**H_2_, 1′-C), 42.2 (CH, 3-C), 51.6 (CH, 5-C), 115.4 (2 × CH, d, *J* = 20.1 Hz, *m*-Ar), 118.7 (CH_2_, 3′-C), 130.6 (2 × CH, d, *J* = 8.8 Hz, *o*-Ar), 133.7 (CH, 2′-C), 134.9 (C, Ar), 161.8 (C, d, *J* = 243.9 Hz, CF), 178.7 (C=O).

General procedure B: Olefin metathesis.

A solution of allyl derivatives **11a** or **11b** (1 equiv) in dry DCM was placed in a 25 mL round-bottom flask equipped with a magnetic stirring bar. The corresponding alkene (3 equiv) was added, followed by the addition of 1st generation Grubbs’ catalyst (0.1 equiv). The reaction mixture was heated to 40 °C and stirred for 40 h. After this period, the solvent was removed under reduced pressure, and the residue was purified by flash column chromatography using EtOAc:Hexane (70:30) to afford the corresponding product.

(3*R*,5*R*)-3-Benzyl-5-((*E*)-undec-2-en-1-yl)pyrrolidin-2-one (**4a**).

According to General Procedure B: A solution of **11a** (0.35 g, 0.16 mmol) in CH_2_Cl_2_ (2.0 mL) was treated with 1-decene (0.10 mL, 0.48 mmol) and Grubbs catalyst (0.013 g, 0.016 mmol). The crude product was purified by flash column chromatography using EtOAc:Hexane (70:30) as eluent to afford compound **4a** (0.039 g, 74%) as a dark yellow oil; [α]_D_^20^ = −6.4° (*c* 0.5, CHCl_3_). Anal. Calcd for C_22_H_33_NO; C, 80.68; H, 10.16; N, 4.28. Found: C, 80.55; H, 10.19; N, 4.27. ^1^H NMR (500 MHz, CDCl_3_) δ 0.88 (t, *J* = 7.0 Hz, 3H, CH_3_), 1.25 (m, 12H, (CH_2_)_6_), 1.81 (ddd, *J* = 13.1, 8.9, 4.0 Hz, 1H, 1′-H_a_), 1.90–2.10 (m, 3H, 1′-H_b_ +, 4′-H_2_), 2.05 (m, 1H, 4-H_a_), 2.12 (m, 1H, 4-H_b_), 2.65 (dd, *J* = 13.6, 9.9 Hz, 1H, CH_a_Ph), 2.70–2.79 (m, 1H, 3-H), 3.19 (dd, *J* = 13.6, 3.7 Hz, 1H, CH_b_Ph), 3.39–3.46 (m, 1H, 5-H), 5.27 (ddd, *J* = 15.4, 7.9, 6.3 Hz, 1H, 2′-H or 3′-H), 5.45–5.53 (m, 1H, 2′-H or 3′-H), 5.86 (d, *J* = 11.4 Hz, 1H, NH), 7.19–7.23 (m, 1H, *p*-Ar), 7.21 (dd, *J* = 6.5, 1.8 Hz, 2H, *o*-Ar), 7.29 (ddd, *J* = 7.7, 7.2, 1.1 Hz, 2H, *m*-Ar), ^13^C NMR (125.7 MHz, CDCl_3_) δ 14.2 (CH_3_), 22.8 (CH_2_), 29.3 (CH_2_), 29.4 (CH_2_), 29.5 (CH_2_), 29.6 (CH_2_), 29.7 (CH_2_), 32.0 (CH_2_), 32.7 (CH_2_), 37.0 (CH_2_, CH_2_Ph), 39.9 (**C**H_2_, 4-C), 42.3 (CH, 3-C), 52.1 (CH, 5-C), 124.1 (CH, 2′-C or 3′-C), 124.8 (CH, 2′-C or 3′-C), 126.5 (CH, *p*-Ar), 128.6 (2 × CH, *o*-Ar), 129.2 (2 × CH, *m*-Ar), 139.5 (C, Ar), 178.9 (C=O).

(3*R*,5*R*)-3-Benzyl-5-cinnamylpyrrolidin-2-one (**4b**).

According to General Procedure B: A solution of **11b** (0.040 g, 0.19 mmol) in CH_2_Cl_2_ (2.0 mL) was treated with styrene (0.06 mL, 0.56 mmol) and Grubbs catalyst (0.015 g, 0.019 mmol). The crude product was purified by flash column chromatography using EtOAc:Hexane (70:30) as eluent to afford compound **4b** (0.037 g, 68%) as a brown solid; m.p. 91–93 °C; [α]_D_^20^ = −28.1° (*c* 0.2, CHCl_3_). Anal. Calcd for C_22_H_32_FNO; C, 76.48; H, 9.34; N, 4.05. Found: C, 76.45; H, 9.35; N, 4.06. ^1^H NMR (500 MHz, CDCl_3_) δ 1.88 (ddd, *J* = 13.1, 8.9, 4.0 Hz, 1H, 1′-H_a_), 2.03 (dt, *J* = 13.1, 7.9 Hz, 1H, 1′-H_b_), 2.27–2.39 (m, 2H, 4′-H_2_), 2.67 (dd, *J* = 13.6, 9.8 Hz, 1H, CH_a_Ph), 2.74–2.81 (m, 1H, 3-H), 3.18 (dd, *J* = 13.6, 4.0 Hz, 1H, CH_b_Ph), 3.56 (m, 1H, 5-H), 6.08 (ddd, *J* = 15.8, 7.9, 6.7 Hz, 1H, 2′-H), 6.31 (br s, 1H, NH), 6.44 (d, *J* = 15.8 Hz, 1H, 3′-H), 7.16–7.24 (m, 4H, Ar), 7.27–7.35 (m, 6H, Ar). ^13^C NMR (125.7 MHz, CDCl_3_) δ 32.0 (CH_2_, 1′-C), 37.0 (CH_2,_ CH_2_Ph), 40.3 (CH_2_, 4-C), 42.2 (CH, 3-C), 52.1 (CH, 5-C), 125.1 (CH, 2′-C), 126.3 (2 × CH, *m*-Ar), 126.5 (CH, *p*-Ar), 127.6 (2 × CH, *o*-Ar), 128.6 (CH, *p*-Ar), 128.7 (2 × CH, *o*-Ar), 129.2 (2 × CH, *m*-Ar), 133.6 (CH, 3′-C), 137.0 (C, Ar), 139.4 (C, Ar), 179.1 (C=O).

(3*R*,5*R*)-3-(4-Fluorobenzyl)-5-((*E*)-undec-2-en-1-yl)pyrrolidin-2-one (**4c**).

According to General Procedure B: A solution of **11a** (0.036 g, 0.16 mmol) in CH_2_Cl_2_ (2.0 mL) was treated with 1-decene (0.09 mL, 0.48 mmol) and Grubbs catalyst (0.013 g, 0.015 mmol). The crude product was purified by flash column chromatography using EtOAc:Hexane (70:30) as eluent to afford compound **4c** (0.037 g, 71%) as a light brown oil; [α]_D_^20^ = −10.4° (*c* 1.0, CHCl_3_). Anal. Calcd for C_20_H_21_NO; C, 82.44; H, 7.26; N, 4.81. Found: C, 82.66; H, 7.28; N, 4.80. ^1^H NMR (500 MHz, CDCl_3_) A mixture of *Z*/*E* isomers was observed δ 0.87 (t, *J* = 6.9 Hz, 3H, CH_3_), 1.20–1.35 (m, 12H, (CH_2_)_6_), 1.78–1.84 (m, 1H, 4′-H_a_), 1.87–2.24 (m, 5H, 1′-H_2_ + 4′-H_b_ + 4-H_2_), 2.60–2.75 (m, 2H, CH_a_Ph + 3-H), 3.08–3.14 (m, 1H, CH_b_Ph), 3.37–3.44 (m, 1H, 5-H), 5.22–5.30 (m, 1H, 3′-H), 5.44–5.53 (m, 1H, 2′-H), 5.89 (d, *J* = 15.6 Hz, 1H, NH), 6.97 (ddd, *J* = 8.7, 8.7, 3.3 Hz, 2H, *m*-Ar), 7.15 (ddd, *J* = 8.6, 5.5, 3.1 Hz, 2H, *o*-Ar). ^13^C NMR (125.7 MHz, CDCl_3_) A mixture of *Z*/*E* isomers was observed δ 14.2 (CH_3_), 22.8 (CH_2_), 27.6 (CH_2_), 29.3/29.4 (CH_2_), 29.5/29.6 (CH_2_), 29.7/29.8 (CH_2_), 31.8/32.0 (CH_2_), 32.7 (CH_2_, 2′-C), 34.4 (CH_2_, 1′-C), 36.1 (CH_2_, CH_2_Ph), 39.9 (CH_2_, 4-C), 42.2 (CH, 3-C), 52.1 (CH, 5-C), 115.4 (2 × CH, d, *J* = 21.4 Hz, m-Ar), 124.0 (CH_,_ 2′-C), 124.7 (CH, 3′-C), 130.6 (2 × CH, d, *J* = 7.5 Hz, *o*-Ar), 134.1 (C, Ar), 161.8 (C, d, *J* = 245.1 Hz, CF), 178.7 (C=O).

(3*R*,5*R*)-5-Cinnamyl-3-(4-fluorobenzyl)pyrrolidin-2-one (**4d**).

According to General Procedure B: A solution of **11b** (0.38 g, 0.16 mmol) in CH_2_Cl_2_ (2.0 mL) was treated with styrene (0.06 mL, 0.48 mmol) and Grubbs catalyst (0.013 g, 0.016 mmol). The crude product was purified by flash column chromatography using EtOAc:Hexane (70:30) as eluent to afford compound **4d** (0.36 g, 73%) as a light brown oil; [α]_D_^20^ = −15.9° (*c* 0.2, CHCl_3_). Anal. Calcd for C_20_H_20_FNO; C, 77.64; H, 6.52; N, 4.53. Found: C, 77.82; H, 6.50; N, 4.53. ^1^H NMR (500 MHz, CDCl_3_) δ 1.89 (ddd, *J* = 12.9, 8.6, 3.9 Hz, 1H, 1′-H_a_), 1.99 (dt, *J* = 13.2, 7.5 Hz, 1H, 1′-H_b_), 2.27–2.40 (m, 2H, 4-H_2_), 2.69 (dd, *J* = 13.5, 9.4 Hz, 1H, CH_a_Ph), 2.70–2.76 (m, 1H, 3-H), 3.11 (dd, *J* = 13.1, 3.5 Hz, 1H, CH_b_Ph), 3.50–3.56 (m, 1H, 5-H), 6.08 (ddd, *J* = 15.9, 8.0, 6.6 Hz, 1H, 2′-H), 6.12 (br s, 1H, NH), 6.42 (d, *J* = 15.9 Hz, 1H, 3′-H), 6.97 (ddd *J* = 8.7, 8.7, 3.3 Hz, 2H, *m*-Ar), 7.16 (ddd, *J* = 8.6, 5.5, 3.1 Hz, 2H, *o*-Ar), 7.23 (ddd, *J* = 6.9, 6.8, 1.8 Hz, 1H, *p*-Ar), 7.31 (m, 4H, *o,m*-Ar). ^13^C NMR (125.7 MHz, CDCl_3_) δ 31.9 (CH_2_, 1′-C), 36.1 (CH_2_, CH_2_Ph), 40.4 (CH_2_, 4-C), 42.1 (CH, 3-C), 52.0 (CH, 5-C), 115.4 (2 × CH, d, *J* = 21.4 Hz, *m*-Ar), 125.0 (CH, 2′-C), 126.3 (2 × CH, *m*-Ar), 127.7 (CH, *p*-Ar), 128.7 (2 × CH, *o*-Ar), 130.6 (2 × CH, d, *J* = 8.8 Hz, *o*-Ar), 133.8 (CH, 3′-C), 134.9 (C, Ar), 136.9 (C, Ar), 161.8 (C, d, *J* = 243.9 Hz, C-F), 178.7 (C=O).

General Procedure C: Catalytic reduction.

A solution of allyl derivatives **11a** and **b**, **4a**–**d** or **15** (1.0 equiv) in MeOH was placed in a 25 mL round-bottom flask equipped with a magnetic stirring bar. Pd/C (20% *w*/*w*) was added, and the reaction mixture was stirred at room temperature for 18 h under an atmosphere of hydrogen. After this period, the mixture was filtered through a pad of Celite^®^, and the filtrate was concentrated under reduced pressure to afford the corresponding product. The resulting products **12a** and **b**, **13a**–**d**, and **18** were used directly in the next step without further purification.

(3*R*,5*R*)-3-Benzyl-5-propylpyrrolidin-2-one (**12a**).

According to General Procedure C: A solution of (3*R*,5*R*)-5-allyl-3-benzylpyrrolidin-2-one **11a** (0.025 g, 0.12 mmol) in MeOH (2.0 mL) was treated with Pd/C (5 mg). The reaction mixture was stirred under the conditions described above to afford compound **12a** (0.024 g, 95%) as a colorless oil; [α]_D_^20^ = −15.5° (*c* 0.5, CHCl_3_). Anal. Calcd for C_14_H_19_NO; C, 77.38; H, 8.81; N, 6.45. Found: C, 77.33; H, 8.83; N, 6.46. ^1^H NMR (500 MHz) CDCl_3_) δ 0.89 (t, *J* = 7.3 Hz, 3H, CH_3_), 1.23–1.32 (m, 2H, 2′-H_2_), 1.34–1.40 (m, 1H, 1′-H_a_), 1.42–1.46 (m, 1H, 1′-H_b_), 1.77 (ddd, *J* = 13.0, 8.9, 4.2 Hz, 1H, 4-H_a_), 1.98 (ddd, *J* = 13.0, 7.8, 7.1 Hz, 1H, 4-H_b_), 2.64 (dd, *J* = 13.6, 10.0 Hz, 1H, CH_a_Ph), 2.74 (dddd, *J* = 10.0, 8.8, 7.9, 4.0 Hz, 1H, 3-H), 3.18 (dd, *J* = 13.7, 3.9 Hz, 1H, CH_b_Ph), 3.38–3.45 (m, 1H, 5-H), 6.30 (br s, 1H, NH), 7.22 (d, *J* = 7.9 Hz, 2H, *o*-Ar), 7.20–7.24 (m, 1H, *p*-Ar)_,_ 7.26–7.32 (m, 2H, *m*-Ar). ^13^C NMR (125.7 MHz, CDCl_3_) δ 14.0 (CH_3_), 19.3 (CH_2_, 2′-C), 32.6 (CH_2_, 1′-C), 37.0 (CH_2_, CH_2_Ph), 38.9 (CH_2_, 4-C), 42.2 (CH, 3-C), 52.3 (CH, 5-C), 126.5 (CH, *p*-Ar), 128.6 (2 × CH, *o*-Ar), 129.2 (2 × CH, *m*-Ar), 139.6 (C, Ar), 179.2 (C=O).

(3*R*,5*R*)-3-(4-Fluorobenzyl)-5-propylpyrrolidin-2-one (**12b**).

According to General Procedure C: A solution of (3*R*,5*R*)-5-allyl-3-(4-fluorobenzyl)pyrrolidin-2-one **11b** (0.020 g, 0.09 mmol) in MeOH (2.0 mL) was treated with Pd/C (4 mg). The reaction mixture was stirred under the conditions described above to afford compound **12b** (0.019 g, 94%) as a colorless oil; [α]_D_^20^ = −23.1° (*c* 1.0, CHCl_3_). Anal. Calcd for C_14_H_18_FNO C, 71.46; H, 7.71; N, 5.95. Found: C, 71.41; H, 7.74; N, 5.95. ^1^H NMR (500 MHz, CDCl_3_) δ 0.89 (t, *J* = 7.2 Hz, 3H, CH_3_), 1.24–1.34 (m, 2H, 2′-H_2_), 1.35–1.50 (m, 2H, 1′-H_2_), 1.77 (ddd, *J* = 13.0, 8.7, 4.1 Hz, 1H, 4-H_a_), 1.95 (ddd, *J* = 13.1, 7.9, 6.9 Hz, 1H, 4-H_b_), 2.65 (dd, *J* = 13.3, 9.5 Hz, 1H, CH_a_Ph), 2.70 (tdd, *J* = 8.2, 8.2, 6.9, 3.6 Hz, 1H, 3-H), 3.10 (dd, *J* = 13.3, 3.7 Hz, 1H, CH_b_Ph), 3.39 (m, 1H, 5-H), 6.53 (br s, 1H, NH), 6.97 (ddd, *J* = 8.9, 8.8, 2.0 Hz, 2H, *m*-Ar), 7.15 (ddd, *J* = 8.7, 5.3, 3.2 Hz, 2H, *o*-Ar). ^13^C NMR (125.7 MHz, CDCl_3_) δ 14.0 (CH_3_), 19.3 (CH_2_, 2′-C), 32.4 (CH_2_, 1′-C), 36.1 (CH_2_, CH_2_Ph), 38.9 (CH_2_, 4-C), 42.3 (CH, 3-C), 52.3 (CH, 5-C), 115.4 (2 × CH, d, *J* = 21.4 Hz, *m*-Ar), 130.5 (2 × CH, d, *J* = 8.8 Hz, *o*-Ar), 135.1 (C, Ar), 161.7 (C, d, *J* = 245.1 Hz, CF), 179.1 (C=O).

(3*R*,5*R*)-3-Benzyl-5-undecylpyrrolidin-2-one (**13a**).

According to General Procedure C: A solution of (3*R*,5*R*)-3-benzyl-5-((*E*)-undec-2-en-1-yl)pyrrolidin-2-one **4a** (0.023 g, 0.07 mmol) in MeOH (2.0 mL) was treated with Pd/C (4 mg). The reaction mixture was stirred under the conditions described above to afford compound **13a** (0.021 g, 91%) as a colorless oil; [α]_D_^20^ = −7.2° (*c* 0.5, CHCl_3_). Anal. Calcd for C_22_H_35_NO; C, 80.19; H, 10.71; N, 4.25. Found: C, 80.28; H, 10.68; N, 4.24. ^1^H NMR (500 MHz, CDCl_3_) δ 0.87 (t, *J* = 6.9 Hz, 3H, CH_3_), 1.20–1.32 (m, 18H, (CH_2_)_9_), 1.33–1.49 (m, 2H, 1′-H_2_), 1.77 (ddd, *J* = 13.0, 8.8, 4.1 Hz, 1H, 4-H_a_), 1.98 (dt, *J* = 13.1, 7.5 Hz, 1H, 4-H_b_), 2.65 (dd, *J* = 13.6, 9.9 Hz, 1H, CH_a_Ph), 2.73 (ddt, *J* = 12.8, 8.9, 4.0 Hz, 1H, 3-H), 3.18 (dd, *J* = 13.6, 4.0 Hz, 1H, CH_b_Ph), 3.36–3.43 (m, 1H, 5-H), 6.19 (br s, 1H, NH), 7.22 (dd, *J* = 8.1, 2.0 Hz, 2H, *o*-Ar), 7.20–7.25 (m, 1H, *p*-Ar), 7.29 (ddd, *J* = 7.6, 7.5, 2.6 Hz, 2H, *m*-Ar). ^13^C NMR (125.7 MHz, CDCl_3_) δ 14.3 (CH_3_), 22.8 (CH_2_), 26.1 (CH_2_), 29.5 (CH_2_), 29.57 (CH_2_), 29.62 (CH_2_), 29.66 (CH_2_), 29.73 (CH_2_), 32.0 (CH_2_), 32.6 (CH_2_), 36.8 (CH_2,_ CH_2_Ph), 37.1 (CH_2_, 1′-C), 42.2 (CH, 3-C), 52.5 (CH, 5-C), 126.5 (CH, *p*-Ar), 128.6 (2 × CH, *o*-Ar), 129.2 (2 × CH, *m*-Ar), 139.6 (C, Ar), 179.1 (C=O).

(3*R*,5*R*)-3-(4-Fluorobenzyl)-5-undecylpyrrolidin-2-one (**13b**).

According to General Procedure C: A solution of (3*R*,5*R*)-3-(4-fluorobenzyl)-5-((*E*)-undec-2-en-1-yl)pyrrolidin-2-one **4b** (0.027 g, 0.08 mmol) in MeOH (2.0 mL) was treated with Pd/C (5 mg). The reaction mixture was stirred under the conditions described above to afford compound **13b** (0.025 g, 92%) as a yellow oil; [α]_D_^20^ = −7.0° (*c* 1.0, CHCl_3_). Anal. Calcd for C_22_H_34_FNO; C, 76.04; H, 9.86; N, 4.03; Found: C, 76.17; H, 9.84; N, 4.04. ^1^H NMR (500 MHz, CDCl_3_) δ 0.87 (t, *J* = 6.9 Hz, 3H, CH_3_), 1.20–1.29 (m, 18H, (CH_2_)_9_), 1.34–1.48 (m, 2H, 1′-H_2_), 1.78 (ddd, *J* = 12.8, 8.5, 4.0 Hz, 1H, 4-H_a_), 1.96 (dt, *J* = 13.0, 7.5 Hz, 1H, 4-H_a_), 2.66 (dd, *J* = 12.9, 9.3 Hz, 1H, CH_a_Ph), 2.68–2.74 (m, 1H, 3-H), 3.12 (dd, *J* = 13.0, 3.2 Hz, 1H, CH_b_Ph), 3.37 (ddd, *J* = 7.1, 7.0, 4.0 Hz, 1H, 5-H), 6.05 (br s, 1H, NH), 6.97 (ddd, *J* = 8.7, 8.7, 3.1 Hz, 2H, *m*-Ar), 7.16 (ddd, *J* = 8.6, 5.5, 3.1 Hz, *o*-Ar). ^13^C NMR (125.7 MHz, CDCl_3_) δ 14.3 (CH_3_), 22.8 (CH_2_), 26.1 (CH_2_), 29.5 (CH_2_), 29.57 (CH_2_), 29.63 (CH_2_), 29.66 (CH_2_), 29.7 (CH_2_), 29.9 (CH_2_), 32.0 (CH_2_), 32.5 (CH_2_), 36.2 (CH_2_, CH_2_Ph), 36.8 (CH_2_, 4-C), 42.1 (CH, 3-C), 52.4 (CH, 5-C), 115.4 (2 × CH, d. *J* = 20.1 Hz, m-Ar), 130.5 (2 × CH, d. *J* = 8.8 Hz, o-Ar), 135.1 (C, Ar), 161.8 (C, d. *J* = 238.8 Hz, CF), 178.8 (C=O).

(3*R*,5*R*)-3-Benzyl-5-(3-phenylpropyl)pyrrolidin-2-one (**13c**).

According to General Procedure C: A solution of (3*R*,5*R*)-3-benzyl-5-cinnamylpyrrolidin-2-one **4c** (0.022 g, 0.08 mmol) in MeOH (2.0 mL) was treated with Pd/C (4 mg). The reaction mixture was stirred under the conditions described above to afford compound **13c** (0.021 g, 95%) as a colorless oil; [α]_D_^20^ = −11.6° (*c* 0.5, CHCl_3_). Anal. Calcd for C_20_H_23_NO; C, 81.87; H, 7.90; N, 4.77. Found: C, 81.95; H, 7.93; N, 4.76. ^1^H NMR (500 MHz, CDCl_3_) δ 1.40–1.55 (m, 2H, 2′-H_2_), 1.55–1.63 (m, 2H, 1′-H_2_), 1.77 (ddd, *J* = 13.0, 8.8, 4.1 Hz, 1H, 4-H_a_), 1.99 (dt, *J* = 13.1, 7.8 Hz, 1H, 4-H_b_), 2.59 (t, *J* = 7.6 Hz, 2H, 3′-H_2_), 2.65 (dd, *J* = 13.5, 9.8 Hz, 1H, CH_a_Ph), 2.72 (ddt, *J* = 8.8, 7.1, 3.9 Hz, 1H, 3-H), 3.18 (dd, *J* = 13.5, 3.9 Hz, 1H, CH_b_Ph), 3.39 (m, 1H, 5-H), 6.15 (br s, 1H, NH), 7.12 (dd, *J* = 8.2, 1.5 Hz, 2H, *o*-Ar), 7.15–7.23 (m, 4H, 2 × *p*-Ar + *m*-Ar), 7.23–7.33 (m, 4H, *o,m*-Ar). ^13^C NMR (125.7 MHz, CDCl_3_) δ 27.9 (CH_2_, 2′-C), 32.6 (CH_2_, 1′C), 35.8 (CH_2_, 3′-C), 36.3 (**C**H_2_, 4-C), 37.0 (CH_2_, **C**H_2_Ph), 42.2 (CH, 3-C), 52.3 (CH, 5-C), 126.1 (CH, *p*-Ar), 126.5 (CH, *p*-Ar), 128.47 (2 × CH, *o*-Ar), 128.54 (2 × CH, *o*-Ar), 128.6 (2 × CH, *m*-Ar), 129.2 (2 × CH, *m*-Ar), 139.5 (C, Ar), 141.8 (C, Ar), 179.1 (C=O).

(3*R*,5*R*)-3-(4-Fluorobenzyl)-5-(3-phenylpropyl)pyrrolidin-2-one (**13d**).

According to General Procedure C: A solution of (3*R*,5*R*)-5-cinnamyl-3-(4-fluorobenzyl)pyrrolidin-2-one **4d** (0.026 g, 0.08 mmol) in MeOH (2.0 mL) was treated with Pd/C (5 mg). The reaction mixture was stirred under the conditions described above to afford compound **13d** (0.024 g, 92%) as a white solid; m.p. 82–83 °C; [α]_D_^20^ = −7.4° (*c* 1.0, CHCl_3_). Anal. Calcd for C_20_H_22_FNO; C, 77.14; H, 7.12; N, 4.50. Found: C, 77.22; H, 7.11; N, 4.51. ^1^H NMR (500 MHz CDCl_3_) δ 1.43–1.68 (m, 4H, 1′-H_2_ + 2′-H_2_), 1.77 (ddd, *J* = 12.8, 8.5, 4.0 Hz, 1H, 4-H_a_), 1.94 (ddd, *J* = 13.0, 7.9, 6.9 Hz, 1H, 4-H_b_), 2.60 (t, *J* = 7.5 Hz, 2H, 3′-H_2_), 2.62–2.76 (m, 2H, CH_a_Ph + 3-H), 3.10 (dd, *J* = 12.6, 3.2 Hz, 1H, CH_b_Ph), 3.34–3.40 (m, 1H, 5-H), 6.06 (br s, 1H, NH), 6.97 (ddd, *J* = 8.9, 8.8, 3.2 Hz, 2H, *m*-Ar), 7.11–7.20 (m, 5H, *p.o*-Ar), 7.27 (dd, *J* = 8.7, 7.3 Hz, 2H, *m*-Ar). ^13^C NMR (125.7 MHz, CDCl_3_) δ 27.8 (CH_2_, 2′-C), 32.4 (CH_2_, 1′-C), 35.8 (CH_2_, 3′-C), 36.1 (**C**H_2_, 4-C), 36.3 (CH_2_Ph), 42.1 (CH, 3-C), 52.3 (CH, 5-C), 115.4 (2 × CH, d. *J* = 20.1 Hz, *m*-Ar), 126.1 (2 × CH, *m*-Ar), 128.5 (CH, *p*-Ar), 128.6 (2 × CH, *o*-Ar), 130.5 ((2 × CH, d. *J* = 8.8 Hz, o-Ar), 135.0 (C, Ar), 141.8 (C, Ar), 161.8 (C, d. *J* = 243.9 Hz, CF), 178.8 (C=O).

(3*R*,5*R*)-1,5-Diallyl-3-benzylpyrrolidin-2-one (**14**).

A solution of (3*R*,5*R*)-5-allyl-3-benzylpyrrolidin-2-one **11a** (0.030 g, 0.1 mmol) in dry DCM (2.0 mL) was placed in a 25 mL round-bottom flask equipped with a magnetic stirring bar and cooled to 0 °C in an ice bath. NaH (60% dispersion in mineral oil) (0.012 g, 0.3 mmol) was added and stirred for 0.5 h. Allyl bromide (0.02 mL, 0.2 mmol) was added dropwise. The resulting reaction mixture was allowed to warm to 40 °C and stirred for 20 h. After this period, a saturated solution of NaHCO_3_ (5 mL) was added and extracted with EtOAc (3 × 10 mL). The combined organic layers were dried over anhydrous Na_2_SO_4_, filtered, and concentrated under reduced pressure. The resulting crude product was purified by flash column chromatography using Hexane:EtOAc (80:20) as the eluent to afford compound **14** (0.029 g, 81%) as a colorless oil; [α]_D_^20^ = −5.4° (*c* 0.2, MeOH). Anal. Calcd for C_17_H_21_NO; C, 79.96; H, 8.29; N, 5.49. Found: C, 80.04; H, 8.31; N, 5.50. ^1^H NMR (500 MHz, CDCl_3_) δ 1.88 (m, 2H, 1′-H_2_), 2.16 (dddt, *J* = 14.0, 8.2, 7.0, 1.2 Hz, 1H, 4-H_a_), 2.33–2.40 (m, 1H, 4-Hb), 2.71 (dd, *J* = 13.8, 9.3 Hz, 1H, CH_a_Ph), 2.82–2.86 (m, 1H, 3-H), 3.24 (dd, *J* = 13.7, 4.1 Hz, 1H, CH_b_Ph), 3.46–3.52 (m, 1H, NCH_a_), 3.55 (dddd, *J* = 8.4, 6.9, 4.9, 3.6 Hz, 1H, NCH_b_), 4.41 (ddt, *J* = 15.5, 4.8, 1.7 Hz, 1H, 5-H), 5.10–5.25 (m, 4H, 2 × CH_2=_), 5.62–5.75 (m, 2H, 2 × CH), 7.20–7.30 (m, 3H, *o,p*-Ar), 7.33 (ddd, *J* = 7.7, 7.3, 1.1 Hz, 2H, *m*-Ar). ^13^C NMR (125.7 MHz, CDCl_3_) δ 29.4 (CH_2_, 1′-C), 37.2 (CH_2_, CH_2_Ph), 37.4 (CH_2_, 4-C), 42.4 (CH, 3-C), 43.4 (CH_2_, N**C**H_2_), 55.0 (CH, 5-C), 117.8 (CH_2_, **C**H_2=_), 118.8 (CH_2_, **C**H_2=_), 126.4 (CH, *p*-Ar), 128.6 (2 × CH, *o*-Ar), 129.3 (2 × CH, *m*-Ar), 132.8 (CH, CH=), 133.1 (CH, CH=), 139.5 (C, Ar), 175.8 (C=O).

(2*R*,8a*R*)-2-Benzyl-1,5,8,8a-tetrahydroindolizin-3(2*H*)-one (**15**).

A solution of (3*R*,5*R*)-1,5-diallyl-3-benzylpyrrolidin-2-one **14** (0.028 g, 0.1 mmol) in dry DCM (2.0 mL) was placed in a 25 mL round-bottom flask equipped with a magnetic stirring bar. 1st generation Grubbs’catalyst (0.009 g, 0.01 mmol) was added. The reaction mixture was heated to 40 °C and stirred for 18 h. After this period, the solvent was removed under reduced pressure, and the residue was purified by flash column chromatography using Hexane:EtOAc (70:30) to afford **15** (0.020 g, 80%) as a yellow oil; [α]_D_^20^ = −3.3° (*c* 0.3, CHCl_3_). Anal. Calcd for C_15_H_17_NO_;_ C, 79.26; H, 7.54; N, 6.16. Found: C, 79.28; H, 7.56; N, 6.16. ^1^H NMR (500 MHz, CDCl_3_) δ 1.75 (ddd, *J* = 13.3, 9.2, 4.4 Hz, 1H, 8-H_a_), 1.84–1.97 (m, 1H, 1-H_a_), 2.03 (ddd, *J* = 13.1, 8.0, 6.2 Hz, 1H, 8-H_b_), 2.12–2.18 (m, 1H, 1-H_b_), 2.73–2.82 (m, 2H, CH_a_Ph + 2-H), 3.16 (dd, *J* = 12.6, 2.7 Hz, 1H, CH_b_Ph), 3.29 (ddt, *J* = 10.6, 8.4, 4.4 Hz, 1H, 5-H_a_), 3.45–3.51 (m, 1H, 5-H_b_), 4.32 (ddd, *J* = 18.6, 3.3, 2.9 Hz, 1H, 8a-H), 5.64–5.69 (m, 1H, 6-H or 7-H), 5.71–5.76 (m, 1H, 6-H or 7-H), 7.15–7.25 (m, 3H, *o,p*-Ar), 7.28 (dd, *J* = 7.8, 7.7 Hz, 2H, *m*-Ar). ^13^C NMR (125.7 MHz, CDCl_3_) δ 30.9 (CH_2_, 1-C), 32.0 (CH_2_, 8-C), 37.4 (CH_2_, CH_2_Ph), 40.5 (CH_2_, 5-C), 42.6 (CH, 2-C), 51.5 (CH, 8a-C), 123.8 (CH, 6-C or 7-C), 124.4 (CH, 6-C or 7-C), 126.5 (CH, *p*-Ar), 128.6 (2 × CH, *o*-Ar), 129.2 (2 × CH, *m*-Ar), 139.4 (C, Ar), 175.2 (C=O).

(2*R*,8a*R*)-2-Benzylhexahydroindolizin-3(2*H*)-one (**18**).

According to General Procedure C: A solution of (2*R*,8a*R*)-2-benzyl-1,5,8,8a-tetrahydroindolizin-3(2*H*)-one **15** (0.017 g, 0.08 mmol) in MeOH (2.0 mL) was treated with Pd/C (3 mg). The reaction mixture was stirred under the conditions described above to afford compound **18** (0.016 g, 93%) as a yellow oil; [α]_D_^20^ = −48.9° (*c* 0.7, CHCl_3_). Anal. Calcd for C_15_H_19_NO_;_ C, 78.56; H, 8.35; N, 6.11. Found: C, 78.53; H, 8.33; N, 6.11. ^1^H NMR (500 MHz, CDCl_3_) δ 1.08 (qd, *J* = 12.7, 3.5 Hz, 1H, 8-H_a_), 1.25–1.38 (m, 2H, 7-Ha + 8-Hb), 1.60–1.69 (m, 2H, 6-H_2_), 1.72 (tdd, *J* = 14.1, 3.3, 1.5 Hz, 1H, 7-H_b_), 1.78–1.84 (m, 1H, 1-H_a_), 1.94 (ddd, *J* = 13.1, 7.7, 4.5 Hz, 1H, 1-H_b_), 2.54 (td, *J* = 12.6, 3.7 Hz, 1H, 5-H_a_), 2.71 (dd, *J* = 13.0, 9.0 Hz, 1H, CH_a_Ph), 2.73–2.81 (m, 1H, 2-H), 2.97–3.04 (m, 1H, 5-H_b_), 3.09 (dd, *J* = 12.9, 3.4 Hz, 1H, CH_b_Ph), 4.10–4.14 (m, 1H, 8a-H), 7.17–7.23 (m, 3H, *o,p*-Ar), 7.28 (dd, *J* = 6.9, 6.7 Hz, 2H, *m*-Ar). ^13^C NMR (125.7 MHz, CDCl_3_) δ 23.9 (CH_2_, 6-C), 24.6 (CH_2_, 7-C), 30.6 (CH_2_, 1-C), 33.4 (CH_2_, 8-C), 37.5 (CH_2_, CH_2_Ph), 40.5 (CH_2_, 5-C), 43.0 (CH, 2-C), 55.9 (CH, 8a-C), 126.5 (CH, p-Ar), 128.5 (2 × CH, *o*-Ar), 129.3 (2 × CH, *m*-Ar), 139.4 (C, Ar), 174.8 (C=O).

General Procedure D: Hydrolysis of lactams.

A solution of derivatives **12a** and **b**, **13a**–**d** or **16** (1 equiv) in 6 N HCl (2.0 mL) was heated to 105 °C and stirred for 3–30 h. After completion of the reaction, as monitored by TLC CH_2_Cl_2_:MeOH (80:20), the solvent was removed under reduced pressure. The resulting solid residue was purified by flash column chromatography using DCM:MeOH (85:15) to afford the corresponding hydrochloride salt.

(2*R*,4*R*)-4-Amino-2-benzylheptanoic acid hydrochloride (**5a**).

According to General Procedure D: A solution of (3*R*,5*R*)-3-benzyl-5-propylpyrrolidin-2-one **12a** (0.014 g, 0.06 mmol) in 6 N HCl was stirred for 3 h under the conditions described above. The crude product was purified by flash column chromatography using DCM:MeOH (85:15) as eluent to afford compound **5a** (0.013 g, 75%) as a yellow oil; [α]_D_^20^ = −9.0° (*c* 0.9, MeOH). Anal. Calcd for (C_14_H_22_ClNO_2_·H_2_O) C, 58.02; H, 8.35; N, 4.83. Found: C, 58.11; H, 8.37; N, 4.82. ^1^H NMR (500 MHz, CD_3_OD) δ 0.94 (t, *J* = 7.3 Hz, 3H, CH_3_), 1.30–1.42 (m, 2H, 6-H_2_), 1.54 (td, *J* = 7.3, 6.6 Hz, 2H, 5-H_2_), 1.65 (ddd, *J* = 14.8, 8.7, 3.9 Hz, 1H, 3-H_a_), 1.97 (ddd, *J* = 14.6, 10.4, 4.1 Hz, 1H, 3-H_b_), 2.78 (dd, *J* = 13.5, 7.4 Hz, 1H, CH_a_Ph), 2.84–2.91 (m, 1H, 2-H), 3.06 (dd, *J* = 13.4, 6.8 Hz, 1H, CH_b_Ph), 3.15–3.21 (m, 1H, 4-H), 7.15–7.26 (m, 3H, *p,o*-Ar), 7.29 (ddd, *J* = 7.3, 7.3, 1.1 Hz, 2H, *m*-Ar). ^13^C NMR (125.7 MHz, CD_3_OD) δ 14.0 (CH_3_, 6-Me), 19.4 (CH_2_, 6-C), 35.4 (CH_2_, 3-C), 36.5 (CH_2_, CH_2_Ph), 39.7 (CH_2_, 5-C), 45.2 (CH, 2-C), 51.3 (CH, 4-C), 127.6 (CH, *p*-Ar), 129.7 (2 × CH, *o*-Ar), 130.1 (2 × CH, *m*-Ar), 140.1 (C, Ar), 178.5 (C=O).

(2*R*,4*R*)-4-Amino-2-(4-fluorobenzyl)heptanoic acid hydrochloride (**5b**).

According to General Procedure D: A solution of (3*R*,5*R*)-3-(4-fluorobenzyl)-5-propylpyrrolidin-2-one **12b** (0.017 g, 0.07 mmol) in 6 N HCl was stirred for 3 h under the conditions described above. The crude product was purified by flash column chromatography using DCM:MeOH (85:15) as eluent to afford compound **5b** (0.016 g, 77%) as a colorless oil; [α]_D_^20^ = −4.8° (*c* 0.5, MeOH). Anal. Calcd for (C_14_H_21_ClFNO_2_·H_2_O) C; 54.63; H, 7.53; N, 4.55. Found: C, 54.52; H, 7.56; N, 4.53. ^1^H NMR (500 MHz, CD_3_OD) δ 0.95 (t, *J* = 7.3 Hz, 3H, 6-Me), 1.34–1.42 (m, 2H, 6-H_2_), 1.56 (q, *J* = 7.0 Hz, 2H, 5-H_2_), 1.66 (ddd, *J* = 14.8, 8.6, 3.8 Hz, 1H, 3-H_a_), 1.98 (ddd, *J* = 14.7, 10.5, 4.2 Hz, 1H, 3-H_b_), 2.79 (dd, *J* = 13.3, 6.9 Hz, 1H, CH_a_Ar), 2.86 (ddd, *J* = 10.4, 7.0, 3.7 Hz, 1H, 2-H), 3.00 (dd, *J* = 13.3, 7.1 Hz, 1H, CH_b_Ar), 3.16–3.22 (m, 1H, 4-H), 7.02 (td, *J* = 8.9, 3.1 Hz, 2H, *m*-Ar), 7.24 (ddd, *J* = 8.7, 5.3, 3.1 Hz, 2H, *o*-Ar). ^13^C NMR (125.7 MHz, CD_3_OD) δ 14.0 (CH_3_, 6-Me), 19.3 (CH_2_, 6-C), 35.5 (CH_2_, 3-C), 36.4 (CH_2_, CH_2_Ar), 38.8 (CH_2_, 5-C), 45.2 (CH, 2-C), 51.4 (CH, 4-C), 116.1 (2 × CH, d, *J* = 22.6 Hz, *m*-Ar), 131.8 (2 × CH, d, *J* = 8.8 Hz, *o*-Ar), 135.9 (C, Ar), 163.2 (C, d, *J* = 242.6 Hz, CF), 178.1 (C=O).

(2*R*,4*R*)-4-Amino-2-benzylpentadecanoic acid hydrochloride (**5c**).

According to General Procedure D: A solution of (3*R*,5*R*)-3-benzyl-5-undecylpyrrolidin-2-one **13a** (0.015 g, 0.04 mmol) in 6 N HCl was stirred for 6 h under the conditions described above. The crude product was purified by flash column chromatography using DCM:MeOH (85:15) as eluent to afford compound **5c** (0.013 g, 73%) as a colorless oil; [α]_D_^20^ = −8.9° (*c* 1.0, MeOH). Anal. Calcd for (C_22_H_38_ClNO_2_·2H_2_O); C, 62.91; H, 10.08; N, 3.33. Found: C, 62.79; H, 10.03 N, 3.34. ^1^H NMR (500 MHz, CD_3_OD) δ 0.90 (t, *J* = 6.5 Hz, 3H, CH_3_), 1.20–1.45 (m, 18H, 9 × CH_2_), 1.50–1.67 (m, 2H, 5-H_2_), 1.68–1.74 (m, 1H, 3-H_a_), 1.87 (ddd, *J* = 14.2, 11.2, 3.3 Hz, 1H, 3-H_b_), 2.69 (dd, *J* = 13.8, 7.8 Hz, 1H, CH_a_Ph), 2.78 (m, 1H, 2-H), 3.11 (dd, *J* = 13.3, 6.3 Hz, 1H, CH_b_Ph), 3.23–3.29 (m, 1H, 4-H), 7.18–7.30 (m, 5H, Ar). ^13^C NMR (125.7 MHz, CD_3_OD) δ 14.4 (CH_3_, Me), 23.2 (CH_2_), 23.7 (CH_2_), 26.2 (CH_2_), 27.4 (CH_2_), 30.2 (CH_2_), 30.5 (CH_2_), 30.6 (CH_2_), 30.7 (CH_2_), 32.9 (CH_2_, 5-C), 33.1 (CH_2_), 35.1 (CH_2_, CH_2_Ph), 39.8 (CH_2_, 3-C), 43.5 (CH, 2-C), 51.2 (CH. 4-C), 127.4 (CH, *p*-Ar), 129.5 (2 × CH, *o*-Ar), 130.1 (2 × CH, *m*-Ar), 140.9 (C, Ar), 176.0 (C=O).

(2*R*,4*R*)-4-Amino-2-benzyl-7-phenylheptanoic acid hydrochloride (**5d**).

According to General Procedure D: A solution of (3*R*,5*R*)-3-benzyl-5-(3-phenylpropyl)pyrrolidin-2-one **13b** (0.013 g, 0.04 mmol) in 6 N HCl was stirred for 3 h under the conditions described above. The crude product was purified by flash column chromatography using DCM:MeOH (85:15) as eluent to afford compound **5d** (0.011 g, 72%) as a colorless oil; [α]_D_^20^ = −4.2° (*c* 0.5, MeOH). Anal. Calcd for (C_20_H_26_ClNO_2_·H_2_O); C, 65.65; H, 7.71; N, 3.83. Found: C, 65.55; H, 7.68; N, 3.81. ^1^H NMR (500 MHz, CD_3_OD) δ 1.51–1.65 (m, 5H, 3-H_a_ + 5-H_2_ + 6-H_2_), 1.86 (ddd, *J* = 13.4, 10.0, 3.5 Hz, 1H, 3-H_b_), 2.60 (t, *J* = 7.2 Hz, 2H, 7-H_2_), 2.67 (dd, *J* = 13.5, 8.2 Hz, 1H, 2-CH_a_Ph), 2.71–2.79 (m, 1H, 2-H), 3.08 (dd, *J* = 13.4, 6.4 Hz, 1H, 2-CH_b_Ph), 3.17–3.24 (m, 1H, 4-H), 7.16 (dd, *J* = 6.9, 1.4 Hz, 2H, *o*-Ar), 7.19–7.28 (m, 8H, Ar). ^13^C NMR (125.7 MHz, CD_3_OD) δ 28.1 (CH_2_), 30.7 (CH_2_, CH_2_Ph), 34.0 (CH_2,_ CH_2_Ph), 35.1 (CH_2_), 36.3 (CH_2_), 39.8 (CH, 2-C), 51.1 (CH, 4-C), 127.0 (CH, *p*-Ar), 127.4 (CH, *p*-Ar), 129.4 (2 × CH, *o*-Ar), 129.5 (2 × CH, *o*-Ar), 129.5 (2 × CH, *m*-Ar), 130.1 (2 × CH, *o*-Ar), 140.8 (C, Ar), 142.8 (C, Ar), 181.1 (C=O).

(2*R*,4*R*)-4-Amino-2-(4-fluorobenzyl)pentadecanoic acid hydrochloride (**5e**).

According to General Procedure D: A solution of (3*R*,5*R*)-3-(4-fluorobenzyl)-5-undecylpyrrolidin-2-one **13c** (0.019 g, 0.05 mmol) in 6 N HCl was stirred for 6 h under the conditions described above. The crude product was purified by flash column chromatography using DCM:MeOH (85:15) as eluent to afford compound **5e** (0.016 g, 73%) as a yellow oil; [α]_D_^20^ = −6.1° (*c* 0.9, MeOH). Anal. Calcd for (C_22_H_37_ClFNO_2_·2H_2_O); 60.33; H, 9.44; N, 3.20. Found: C, 60.38; H, 9.41; N, 3.19. ^1^H NMR (500 MHz, CD_3_OD) δ 0.90 (t, *J* = 7.0 Hz, 3H, CH_3_), 1.20–1.39 (m, 18H, (CH_2_)_9_), 1.50–1.90 (m, 4H, 3-H_2_ + 5-H_2_), 2.65–2.76 (m, 2H, 2-CH_a_Ar + 2-H), 3.07 (dd, *J* = 12.7, 5.8 Hz, 1H, 2-CH_b_Ar), 3.18 (m, 1H, 4-H), 6.99 (td, *J* = 8.9, 4.6 Hz, 2H, *m*-Ar), 7.25 (ddd, *J* = 8.6, 5.3, 2.5 Hz, 2H, *o*-Ar). ^13^C NMR (125.7 MHz, CD_3_OD) δ 14.4 (CH_3_), 23.7 (CH_2_), 26.3 (CH_2_), 30.4 (CH_2_), 30.47 (CH_2_), 30.50 (CH_2_) 30.73 (CH_2_), 30.76 (CH_2_), 33.1 (CH_2_), 34.6 (CH_2_, 3-C or 5-C), 35.2 (CH_2_, CH_2_Ar), 38.9 (CH_2_, 5-C or 3-C), 43.9 (CH, 2-C), 51.2 (CH, 4-C), 116.0 (2 × CH, d, *J* = 21.4 Hz, *m*-Ar), 131.7 (2 × CH, d, *J* = 7.5 Hz, *o*-Ar), 137.0 (C, Ar), 163.0 (C, d, *J* = 242.6 Hz, CF), 175.4 (C=O).

(2*R*,4*R*)-4-Amino-2-(4-fluorobenzyl)-7-phenylheptanoic acid hydrochloride (**5f**).

According to General Procedure D: A solution of (3*R*,5*R*)-3-(4-fluorobenzyl)-5-(3-phenylpropyl)pyrrolidin-2-one **13d** (0.018 g, 0.06 mmol) in 6 N HCl was stirred for 3 h under the conditions described above. The crude product was purified by flash column chromatography using DCM:MeOH (85:15) as eluent to afford compound **5f** (0.015 g, 71%) as a colorless oil; [α]_D_^20^ = −2.4° (*c* 0.7, MeOH). Anal. Calcd for C_20_H_25_ClFNO_2_·H_2_O); C, 62.58; H, 7.09; N, 3.65 Found: C, 62.67; H, 7.11; N, 3.63. ^1^H NMR (500 MHz, CD_3_OD) δ 1.52–1.70 (m, 5H, 5-H_2_ + 6-H_2_ + 3-H_a_), 1.91 (ddd, *J* = 13.9, 10.1, 3.7 Hz, 1H, 3-H_b_), 2.62 (t, *J* = 7.1 Hz, 2H, 7-H_2_), 2.70 (dd, *J* = 13.3, 7.3 Hz, 1H, CH_a_Ar), 2.72–2.78 (m, 1H, 2-H), 3.02 (dd, *J* = 13.1, 6.4 Hz, 1H, CH_b_Ar), 3.17–3.23 (m, 1H, 4-H), 6.99 (ddd, *J* = 8.9, 8.9, 3.1 Hz, 2H, *m*-Ar), 7.17 (d, *J* = 7.0 Hz, 2H, *o*-Ar), 7.22 (dd, *J* = 8.8, 5.3 Hz, 2H, *o*-Ar), 7.26 (m, 3H, *m,p*-Ar). ^13^C NMR (125.7 MHz, CD_3_OD) δ 28.0 (CH_2_, 6-C), 30.8 (CH_2_, 7-C), 33.9 (CH_2_, CH_2_Ar), 35.3 (CH_2_, 5-C), 36.2 (CH_2_, 3-C), 38.9 (CH, 2-C), 51.2 (CH, 4-C), 116.1 (2 × CH, d, *J* = 21.4 Hz, *m*-Ar), 127.1 (2 × CH, *m*-Ar), 129.4 (CH, p-Ar), 129.5 (2 × CH, *o*-Ar), 131.7 (2 × CH, d, *J* = 8.8 Hz, *o*-Ar), 136.5 (C, Ar), 142.7 (C, Ar), 163.1 (C, d, *J* = 242.6 Hz, CF). The (C) signal corresponding to the C=O was not observed.

(*R*)-2-Benzyl-3-((*R*)-piperidin-2-yl)propanoic acid hydrochloride (**6**).

According to General Procedure D: A solution of (2*R*,8a*R*)-2-benzylhexahydroindolizin-3(2*H*)-one **18** (0.012 g, 0.05 mmol) in 6 N HCl was stirred for 30 h under the conditions described above. The crude product was purified by flash column chromatography using DCM:MeOH (85:15) as eluent to afford compound **6** (0.008 g, 52%) as a yellow oil; [α]_D_^20^ = −2.1° (*c* 1.0, MeOH). Anal. Calcd for (C_15_H_22_ClNO_2_·H_2_O) C, 59.69; H, 8.02; N, 4.64. Found: C, 59.61; H, 8.05; N, 4.65. ^1^H NMR (500 MHz, CD_3_OD) δ 0.78–0.97 (m, 2H, 6-H_2_), 1.30–2.00 (m, 6H, 3-H_2_ + 5-H_2_ + 7-H_2_), 2.75–3.20 (m, 6H, CH_2_Ph + CH_2_N + 2-H + 4-H), 7.15–7.40 (m, 5H, Ph). ^13^C NMR (125.7 MHz, CD_3_OD) δ 23.1 (CH_2_, 7-C), 23.3 (CH_2_, 6-C), 23.7 (CH_2_, 5-C), 30.7 (CH_2_, CH_2_Ph), 33.0 (CH_2_, 3-C), 41.5 (CH, 2-C), 46.2 (CH_2_, 8-C), 57.5 (CH, 4-C), 127.7 (CH, p-Ar), 129.6 (2 × CH, *o*-Ar), 130.2 (2 × CH, *m*-Ar), 139.8 (C, Ar), 177.7 (C=O).

## 4. Conclusions

In summary, we have developed an efficient and stereoselective synthetic route to both acyclic and conformationally constrained α,γ-disubstituted γ-amino acids starting from *L*-pyroglutamic acid. The strategy relies on a highly diastereoselective C4 alkylation followed by a decarboxylative allylation sequence, providing *trans*-3,5-disubstituted γ-lactams with excellent diastereiosmeric ratio (dr > 98:2). The synthetic utility of these intermediates was demonstrated through cross-metathesis, hydrogenation, and hydrolysis reactions, affording acyclic α,γ-disubstituted γ-amino acids in good overall yields (71–77%) and with high diastereiosmeric ratio (dr = 92:8 and >98:2). Furthermore, a *C*-allylation/*N*-allylation sequence followed by ring-closing metathesis enabled access to previously unexplored conformationally constrained α,γ-disubstituted γ-amino acids.

Compared with previously reported approaches, this methodology employs readily available chiral starting materials and provides efficient access to structurally diverse and underexplored γ-amino acid derivatives with high levels of stereocontrol. The incorporation of fluorinated substituents and conformational constraints further expands the chemical space of γ-amino acids, features of considerable interest in medicinal chemistry. Although the present study focused on aromatic and fluorinated substrates, further investigations are underway to broaden the substrate scope and evaluate the antimicrobial potential of these compounds, as well as their incorporation into peptide frameworks.

## Data Availability

Data contained within the article or Supplementary Materials.

## Supplementary Materials

The following supporting information can be downloaded at: https://www.mdpi.com/article/10.3390/molecules31122087/s1.
