# Supplementary material for: Decarboxylative-Allylation of Pyroglutamic Acid Derivatives: Stereocontrolled Access to Acyclic and Conformationally Restricted α,γ-Disubstituted γ-Amino Acids
Source: Molecules. 2026 Jun 14;31(12):2087. doi: 10.3390/molecules31122087 (PMC13306036; doi:10.3390/molecules31122087)

# Decarboxylative–Allylation of Pyroglutamic Acid Derivatives: Stereocontrolled Access to Acyclic and Conformationally Restricted α,γ-Disubstituted γ-Amino Acids

Hugo Casas-Morales[a], Dácil Hernández [b], Mario Ordóñez[a],
Alicia Boto*[b] and Ivan Romero-Estudillo*[a]

***tert*-butyl (*S*)-5-oxopyrrolidine-2-carboxylate (16).**

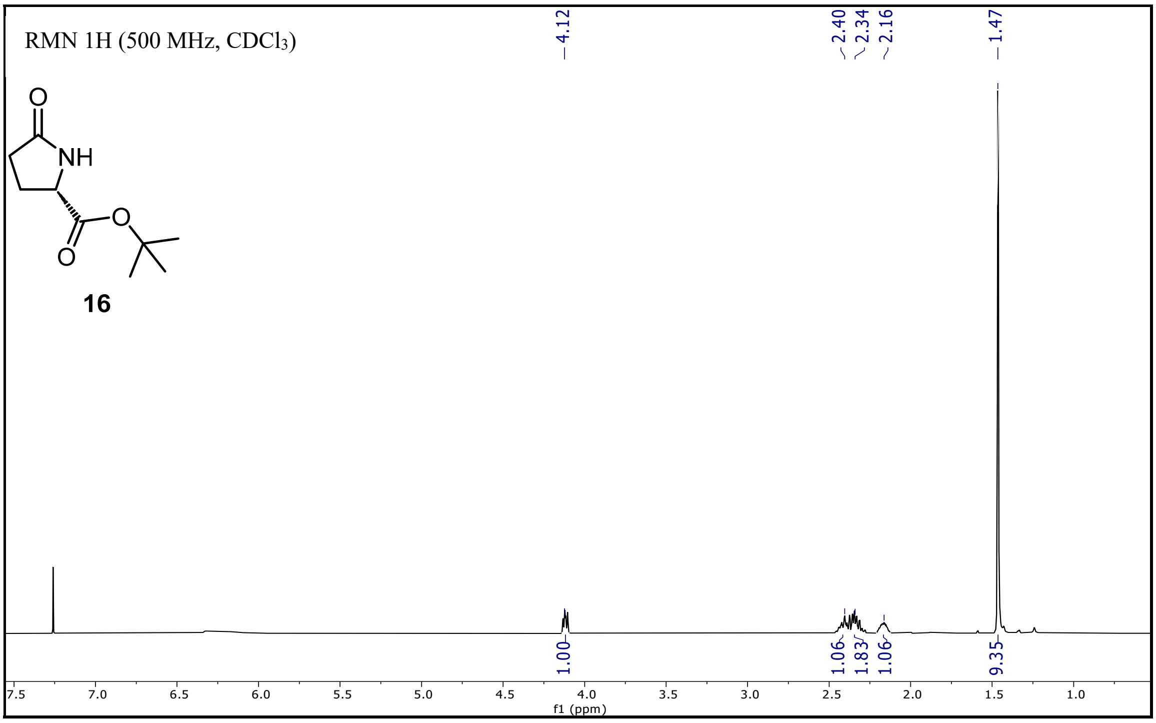

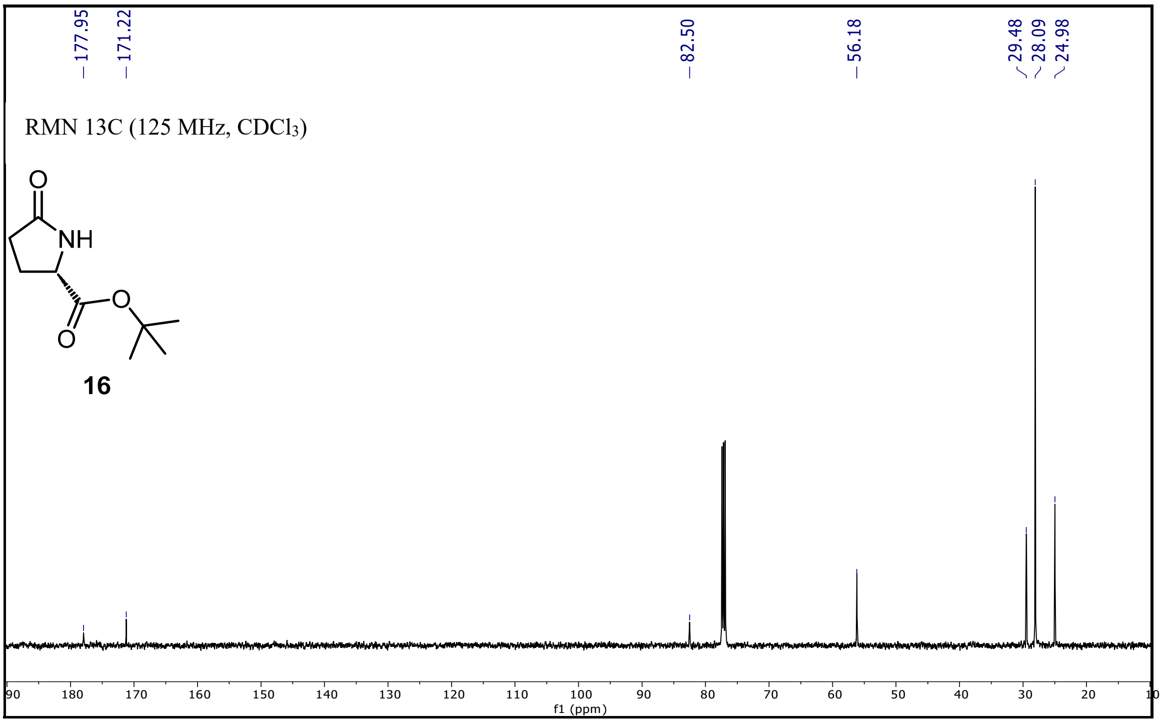


**di-*tert*-butyl (*S*)-5-oxopyrrolidine-1,2-dicarboxylate (7).**

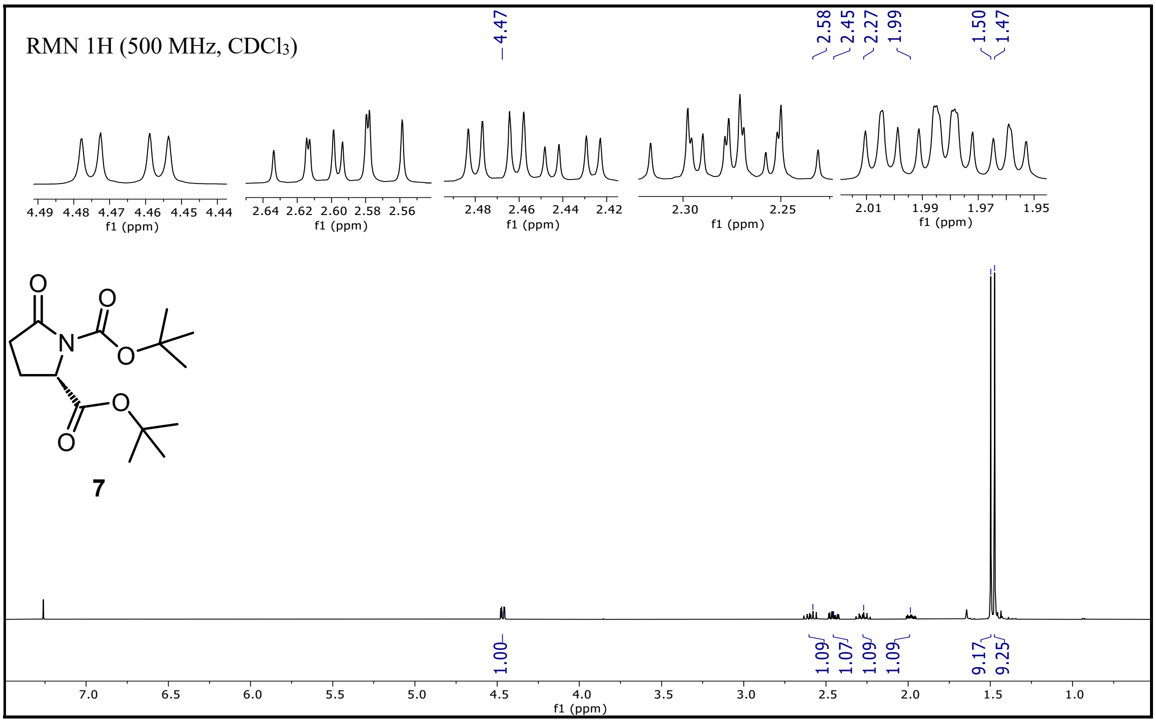

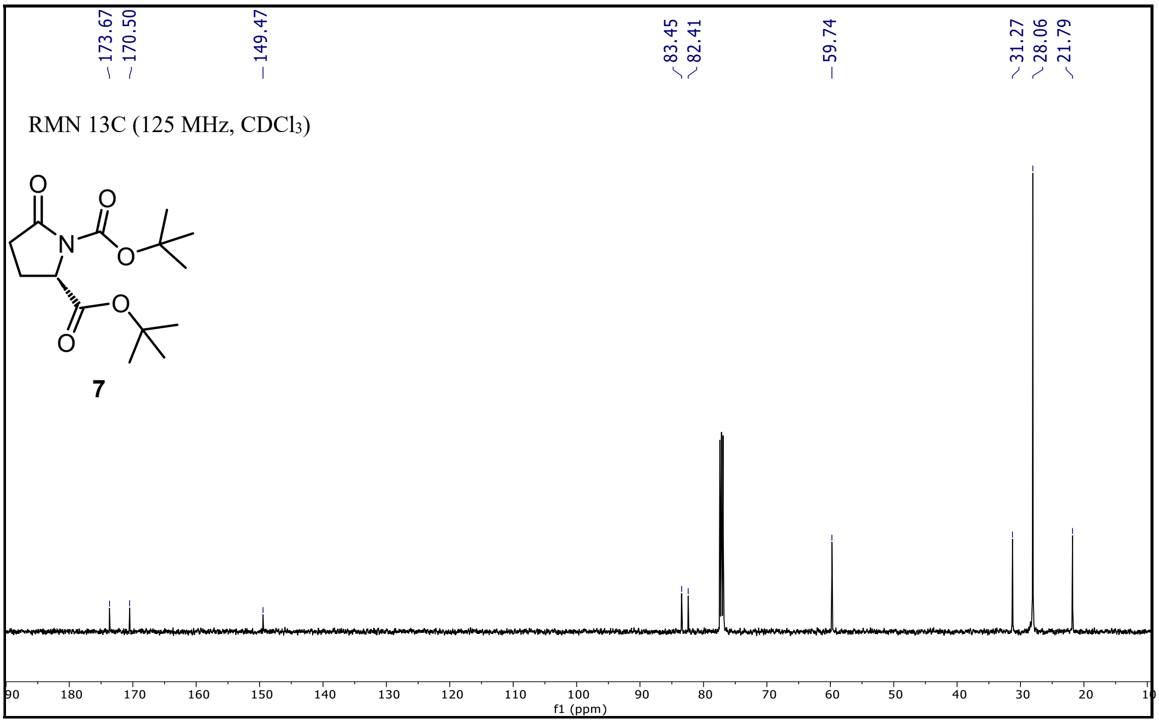


**di-*tert*-butyl (2*S*)-4-benzyl-5-oxopyrrolidine-1,2-dicarboxylate (8a).**

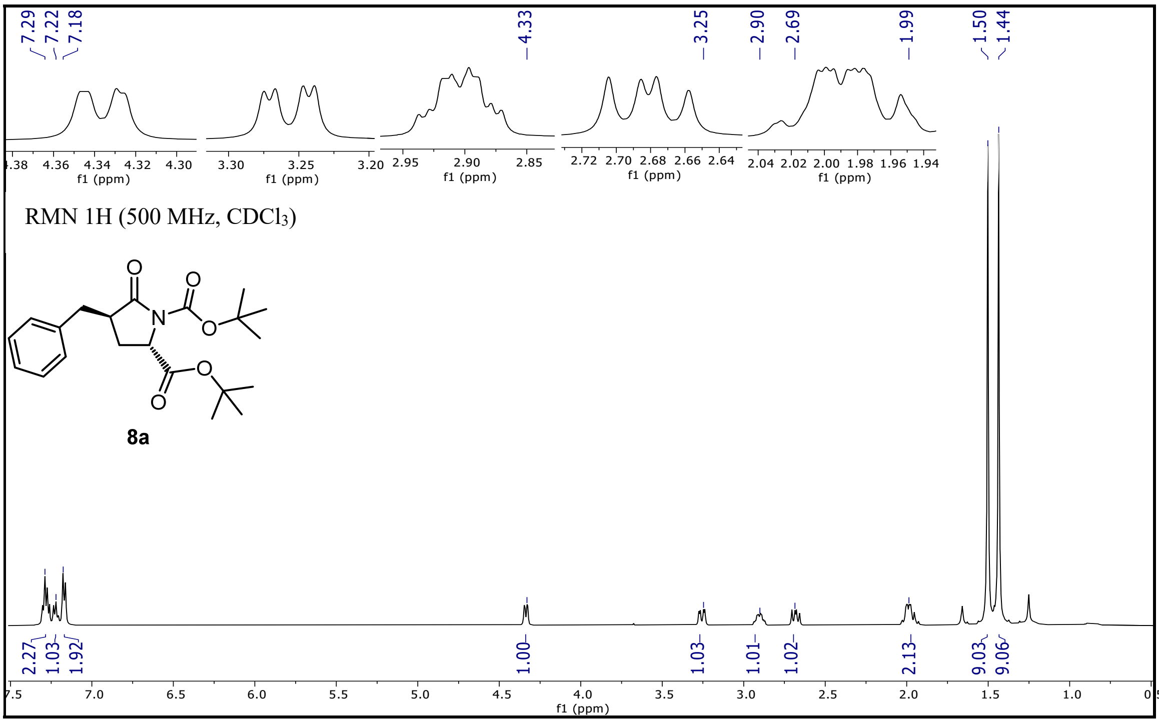

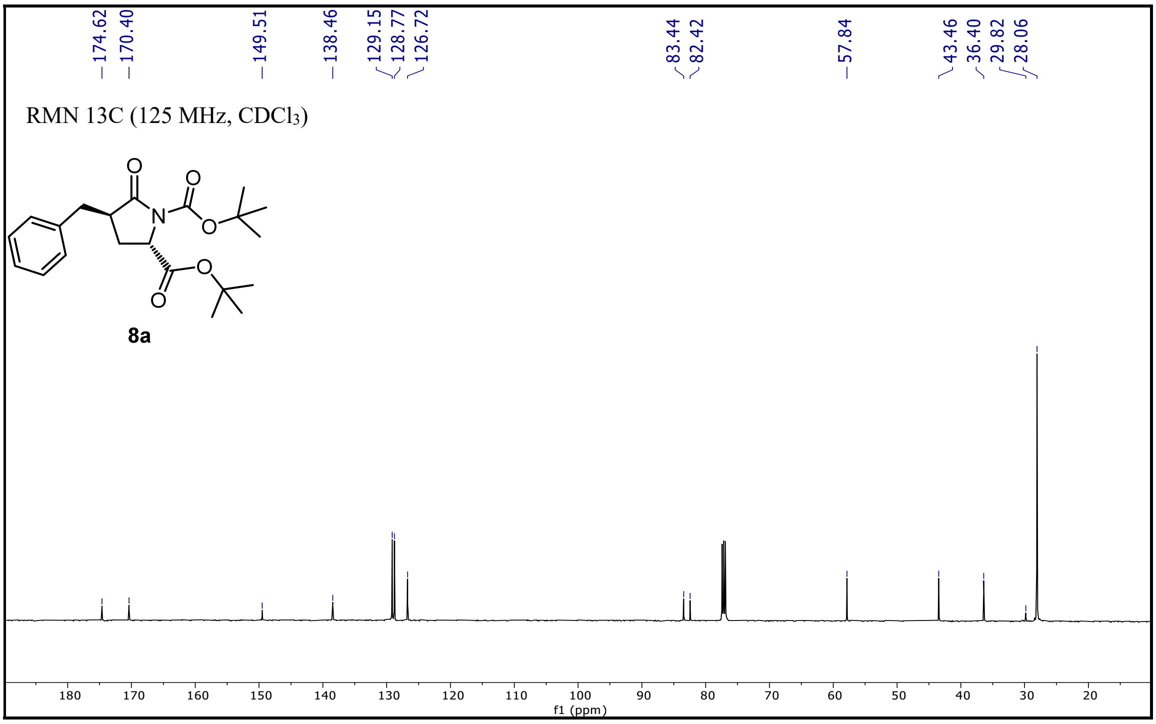


**di-*tert*-butyl (2*S*)-4-(4-fluorobenzyl)-5-oxopyrrolidine-1,2-dicarboxylate (8b).**

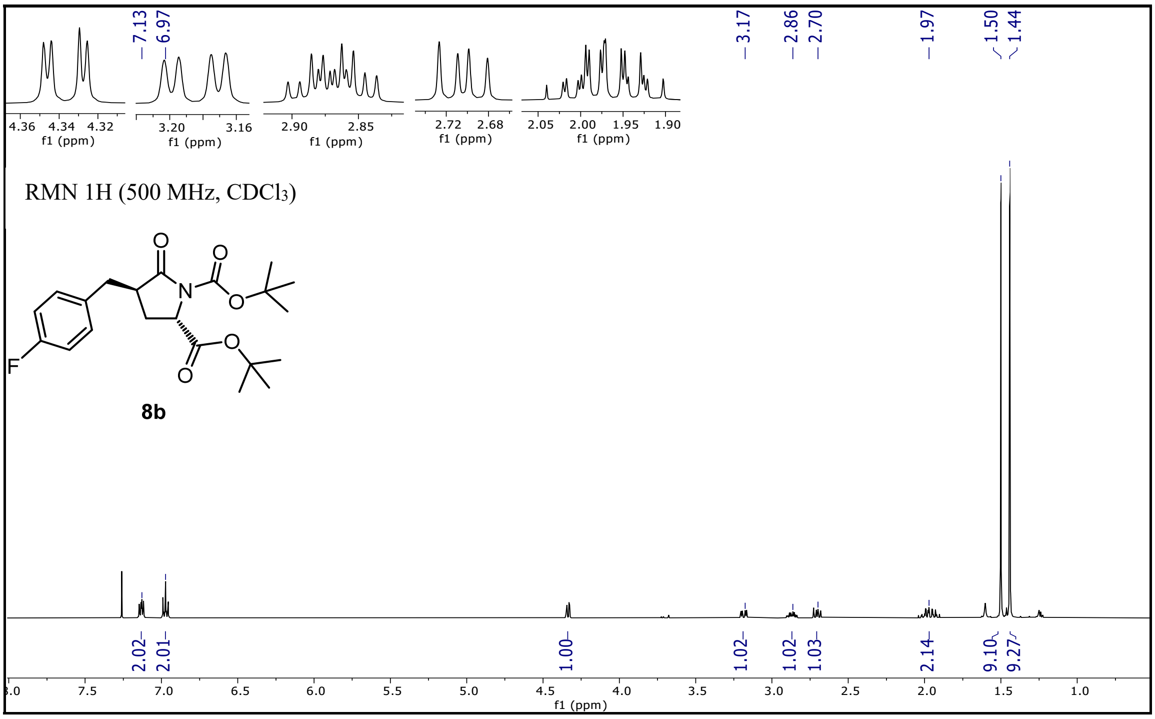

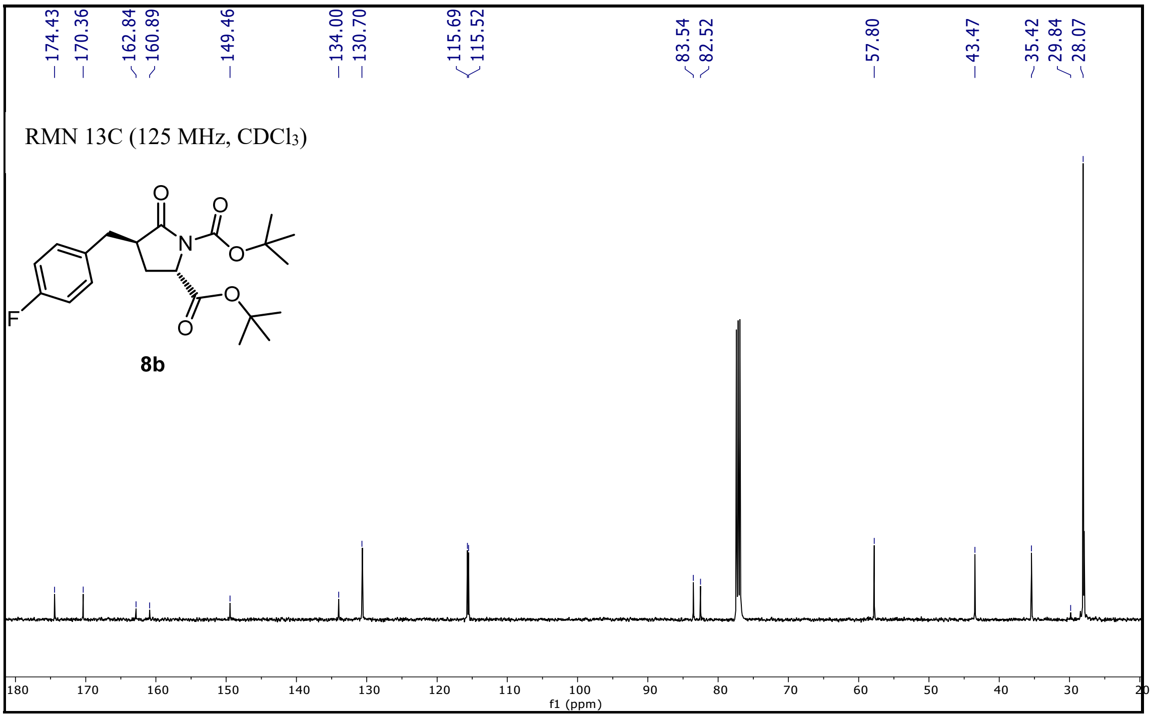


**_tert_-butyl (2_S_,4_R_)-4-benzyl-5-oxopyrrolidine-2-carboxylate (9a).**

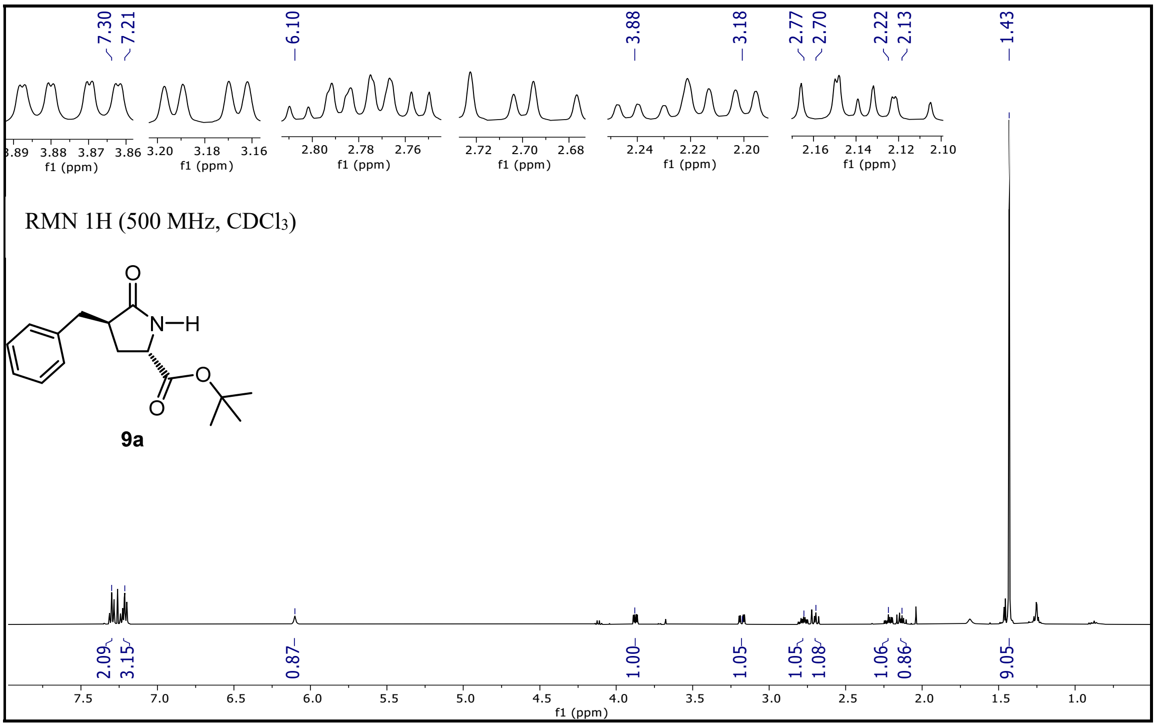

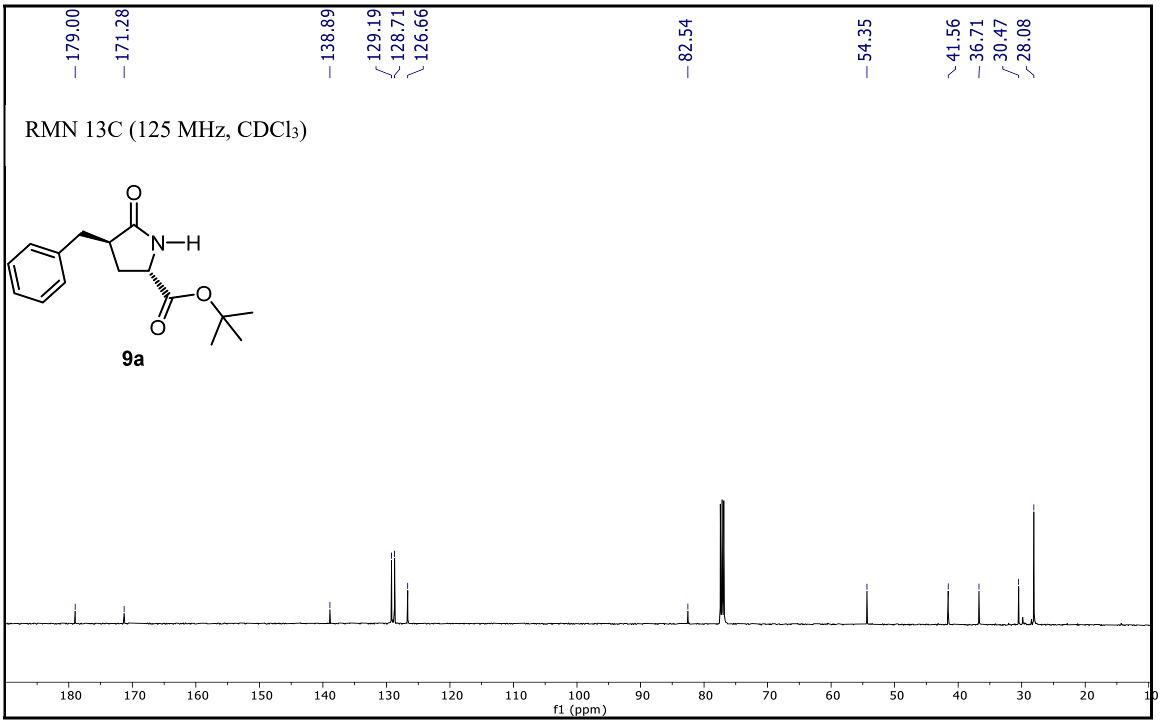


## *tert*-butyl (2*S*,4*R*)-4-(4-fluorobenzyl)-5-oxopyrrolidine-2-carboxylate (9b).

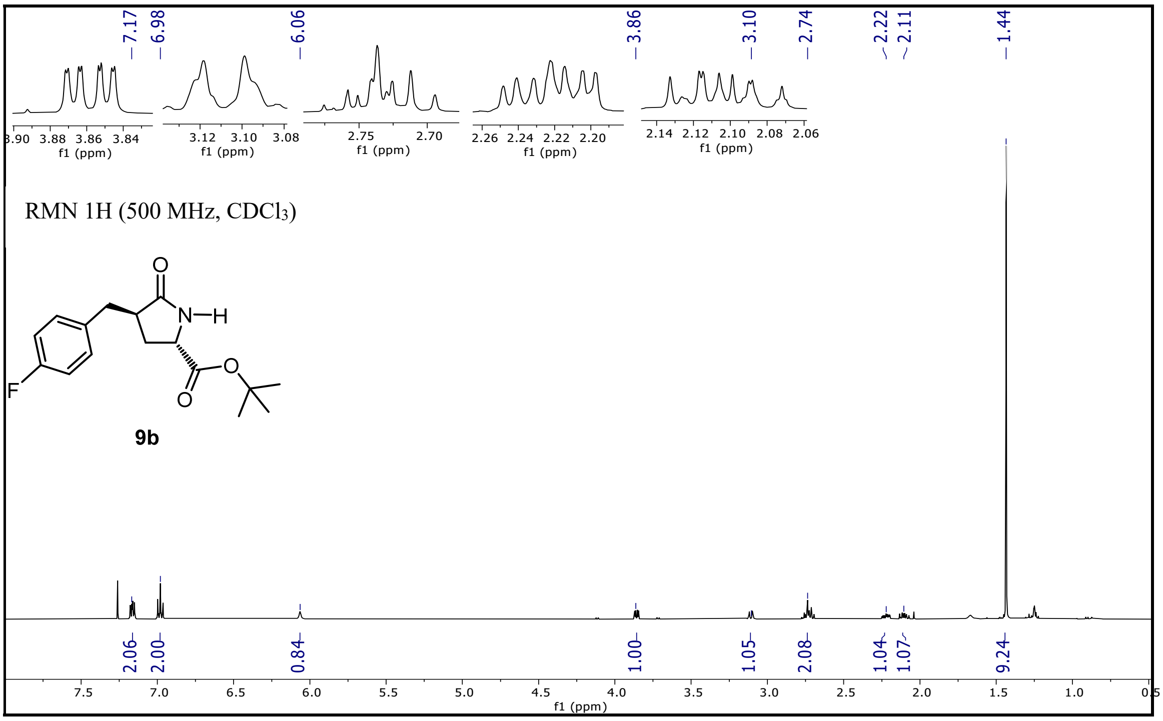

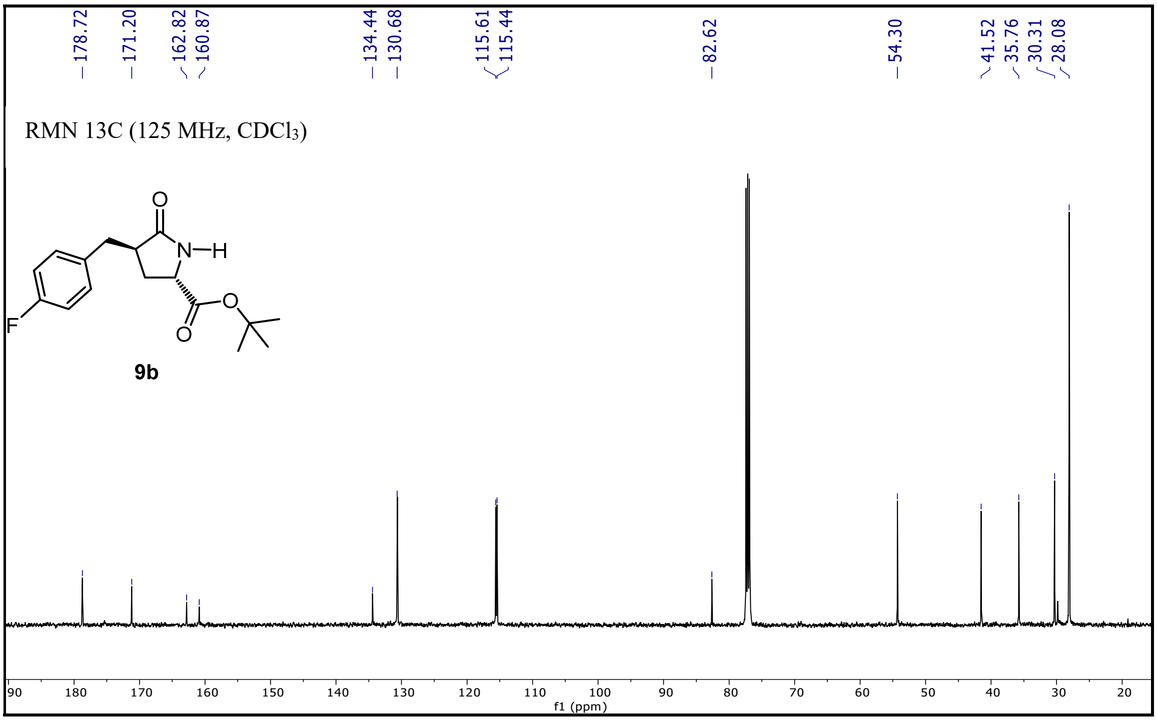


**(2*S*,4*R*)-4-benzyl-5-oxopyrrolidine-2-carboxylic acid (3a).**

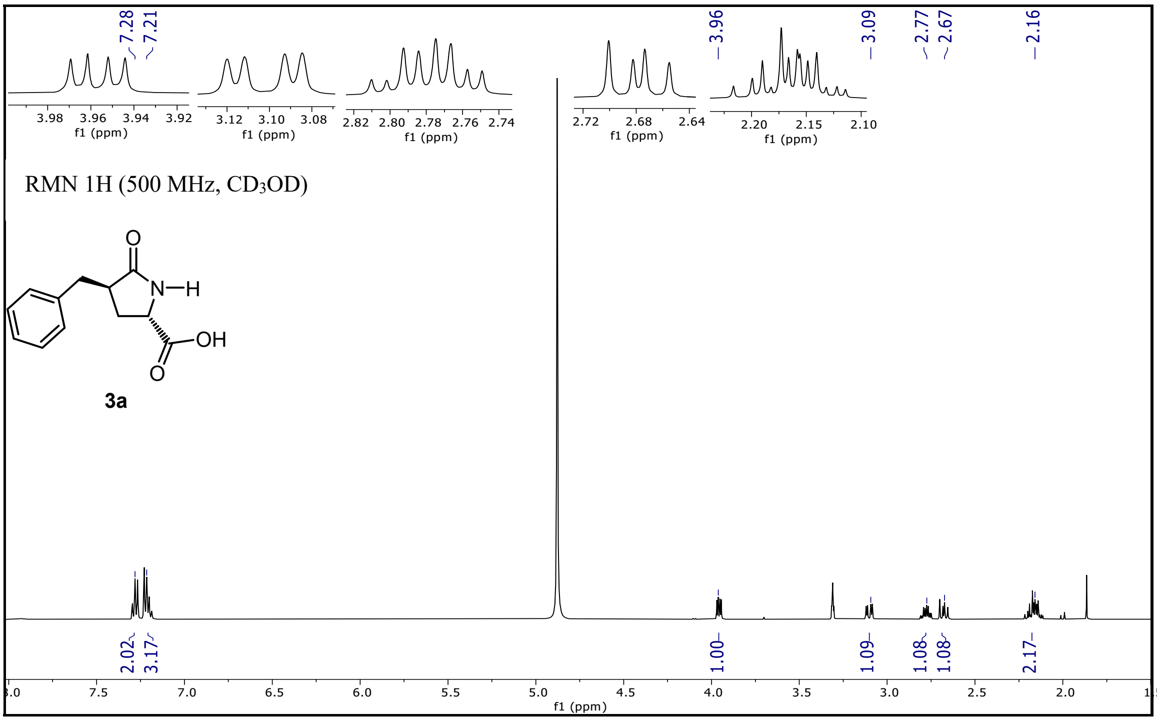

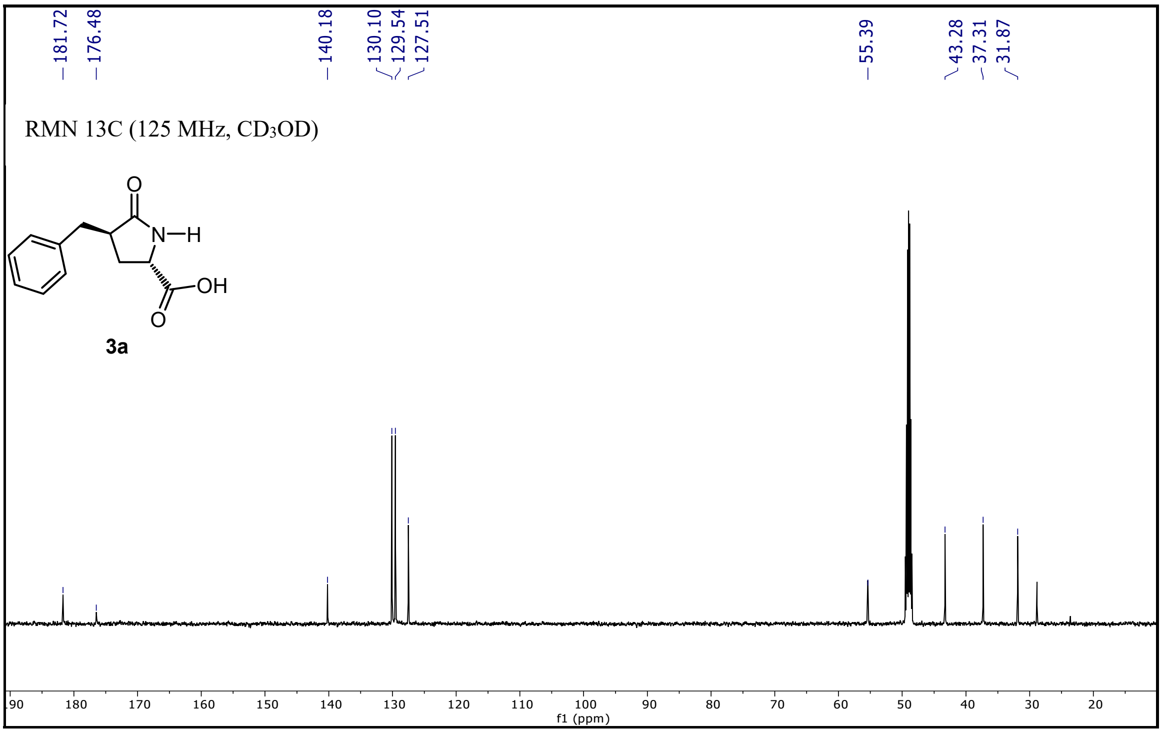


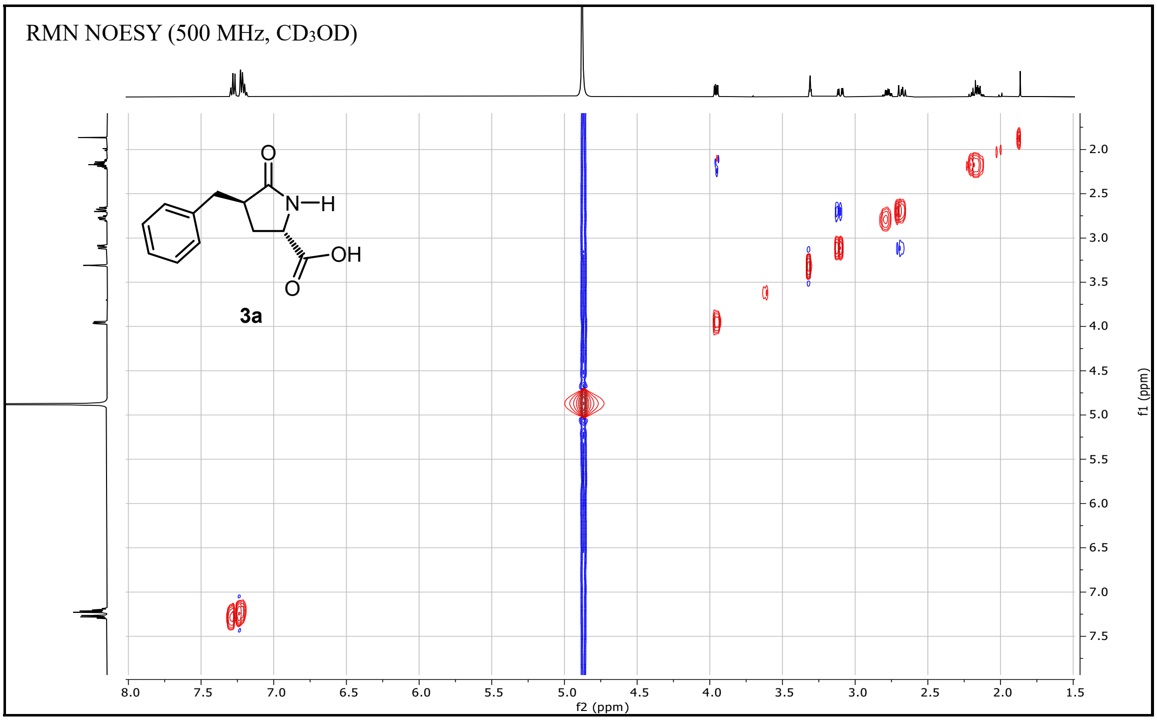


**(2*S*,4*R*)-4-(4-fluorobenzyl)-5-oxopyrrolidine-2-carboxylic acid (3b).**

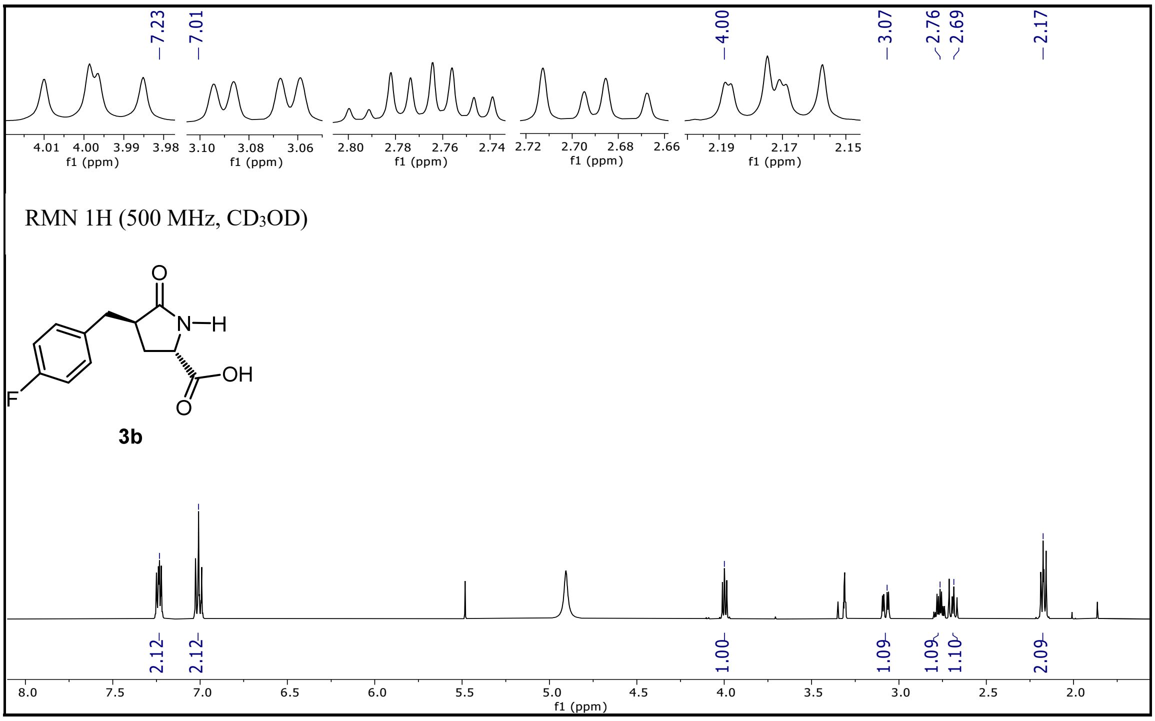


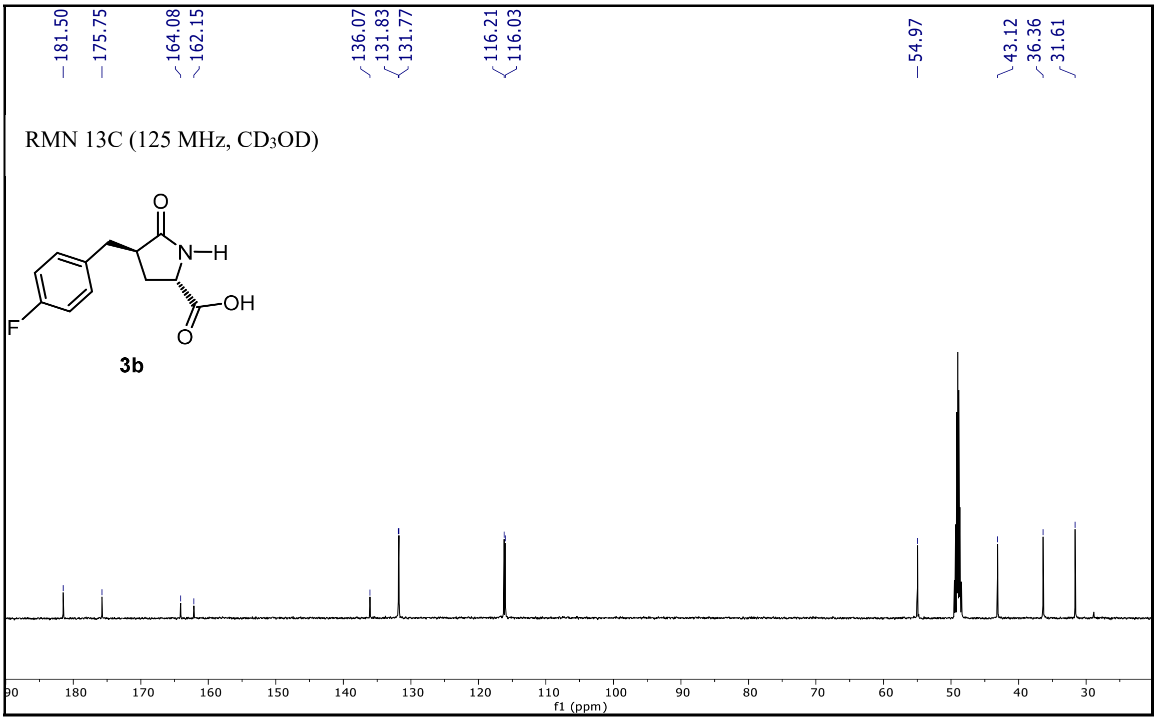


**(3*R*,5*R*)-5-allyl-3-benzylpyrrolidin-2-one (11a).**

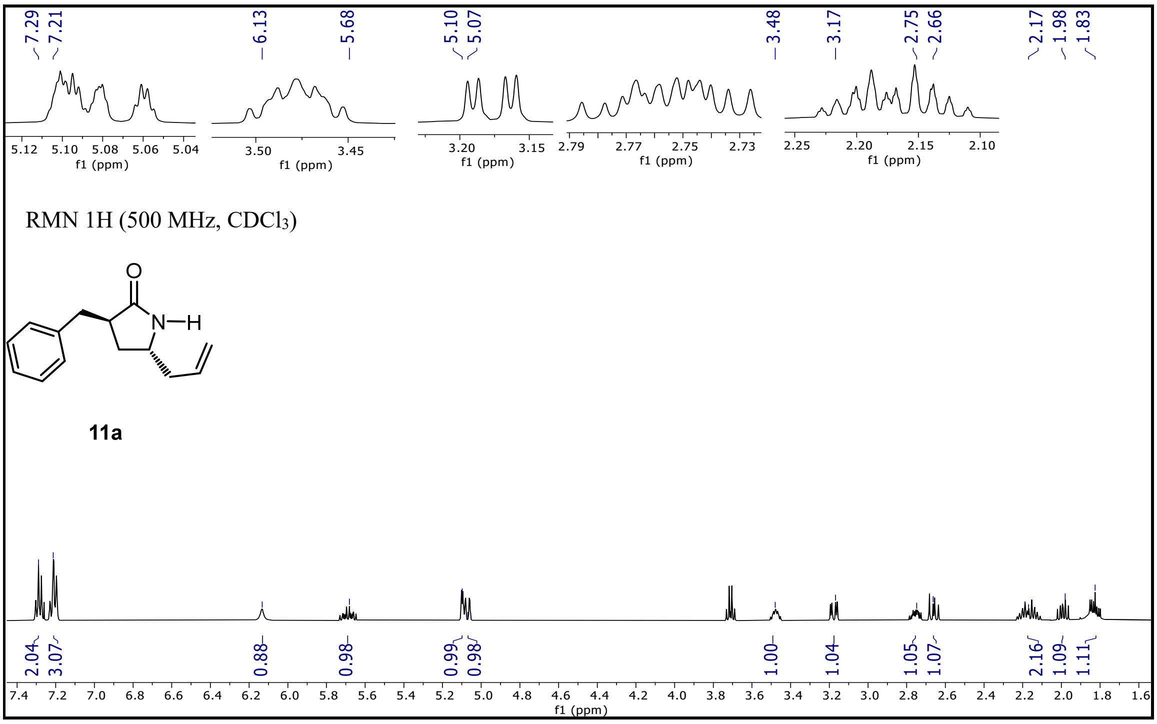

RMN 1H (500 MHz, CDCl3)
11a
7.29
7.21
6.13
5.68
5.10
5.07
3.48
3.17
2.75
2.66
2.17
1.98
1.83
2.04
3.07
0.88
0.98
0.99
0.98
1.00
1.04
1.05
1.07
2.16
1.09
1.11
f1 (ppm)


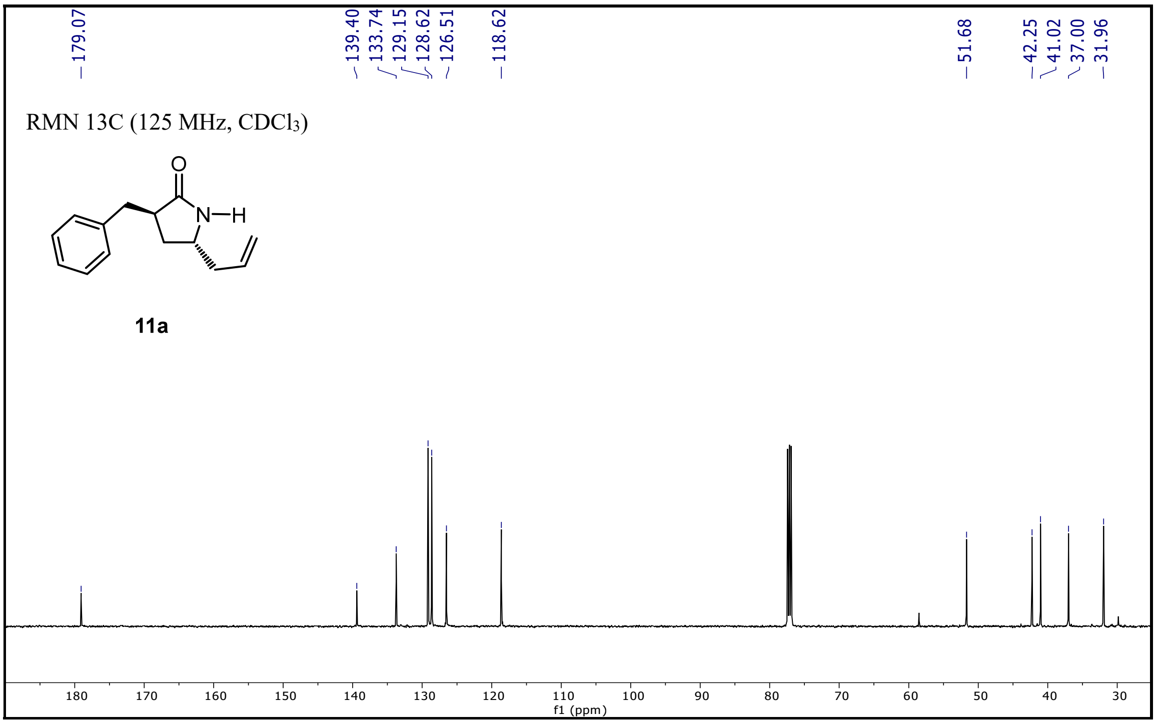

RMN 13C (125 MHz, CDCl3)
11a
179.07
139.40
133.74
129.15
128.62
126.51
118.62
51.68
42.25
41.02
37.00
31.96
f1 (ppm)

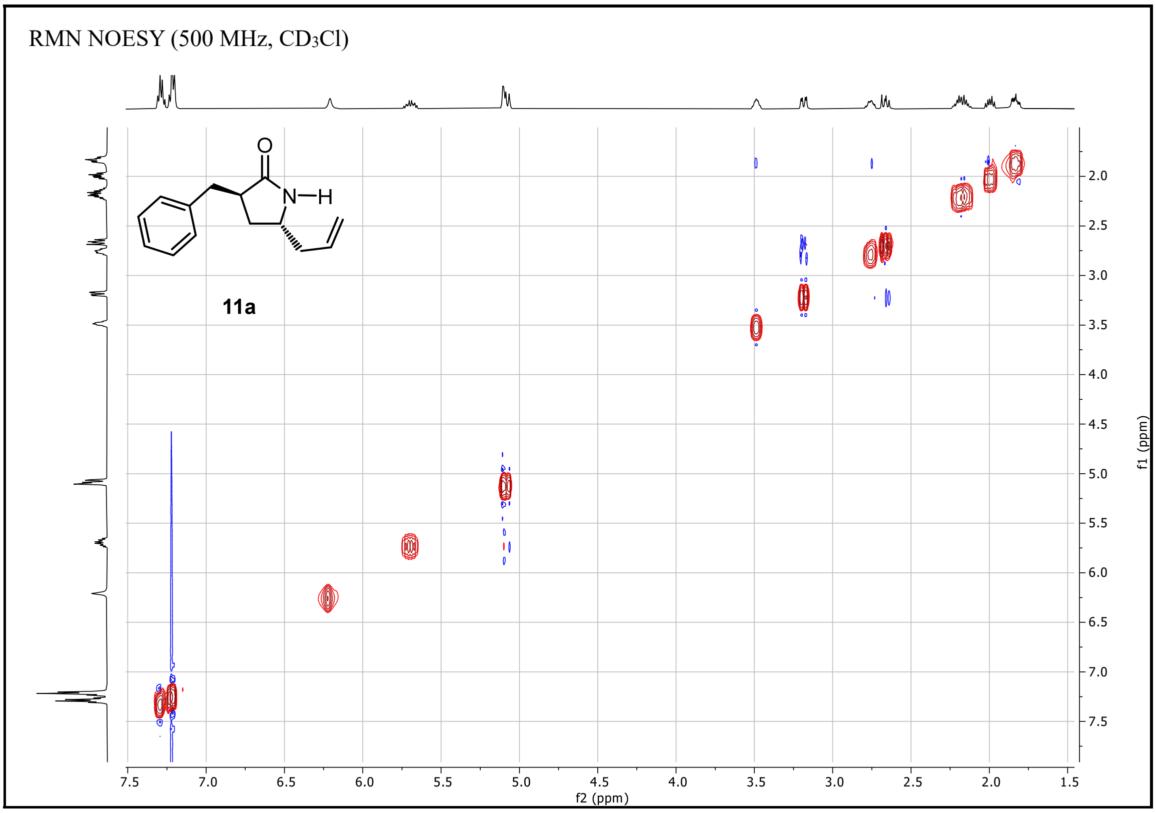


**(3*R*,5*R*)-5-allyl-3-(4-fluorobenzyl)pyrrolidin-2-one (11b).**

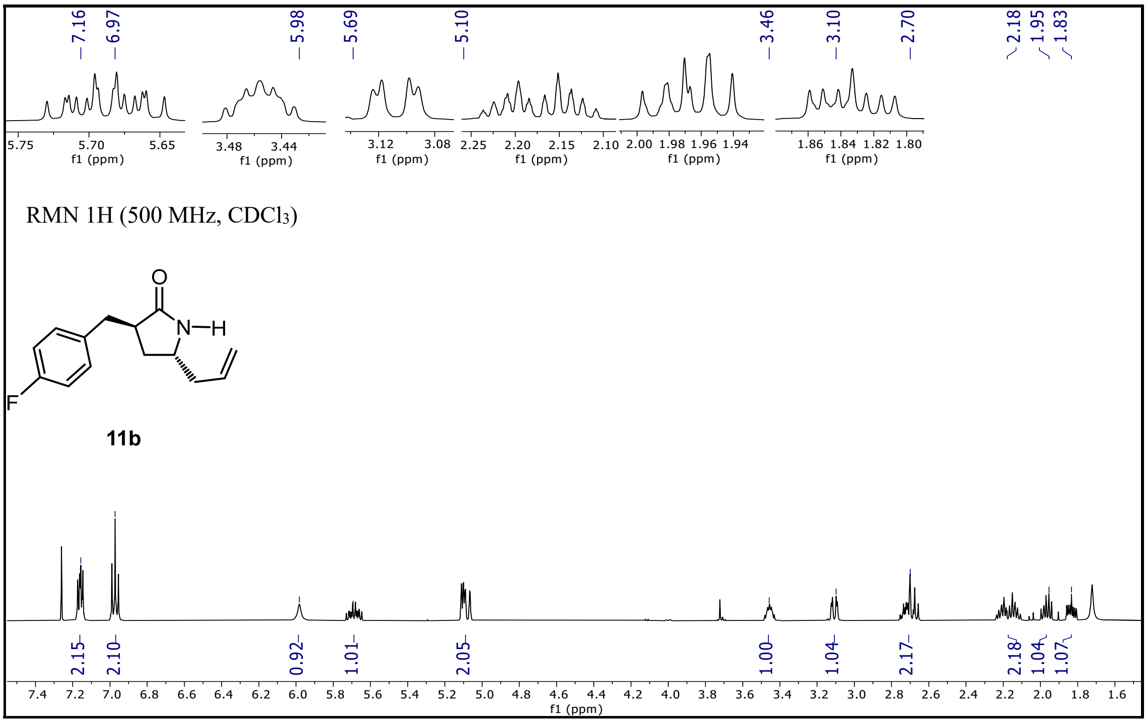


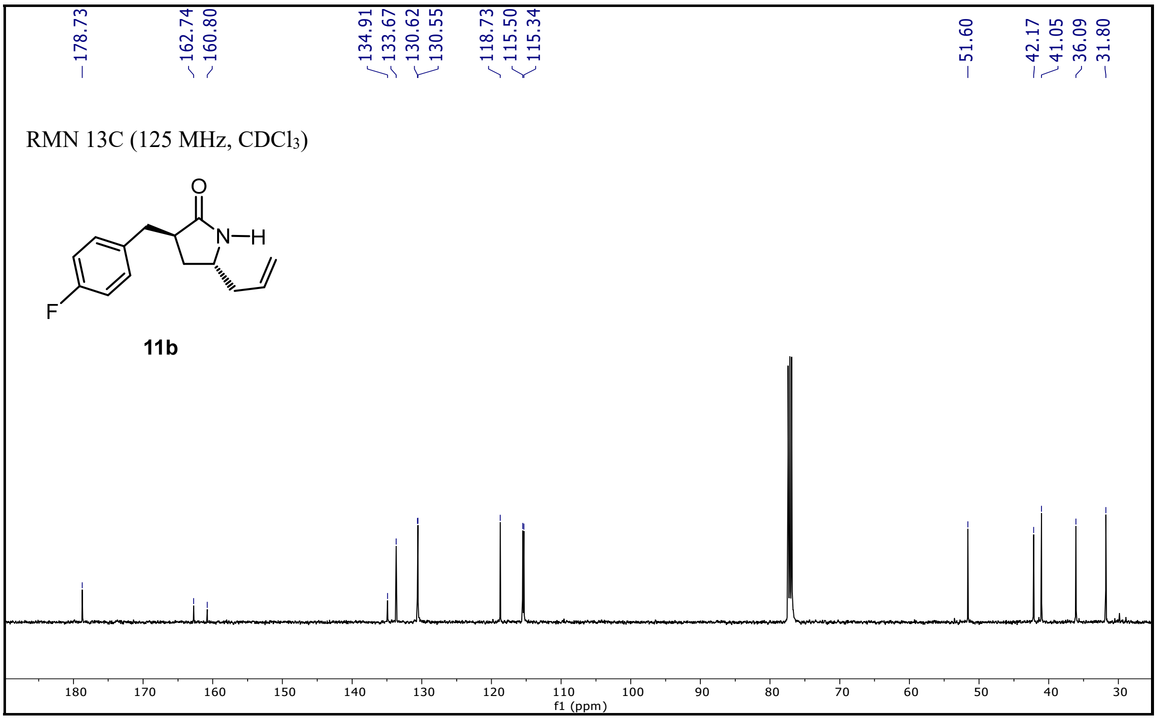


**(3*R*,5*R*)-3-benzyl-5-((*E*)-undec-2-en-1-yl)pyrrolidin-2-one (4a).**

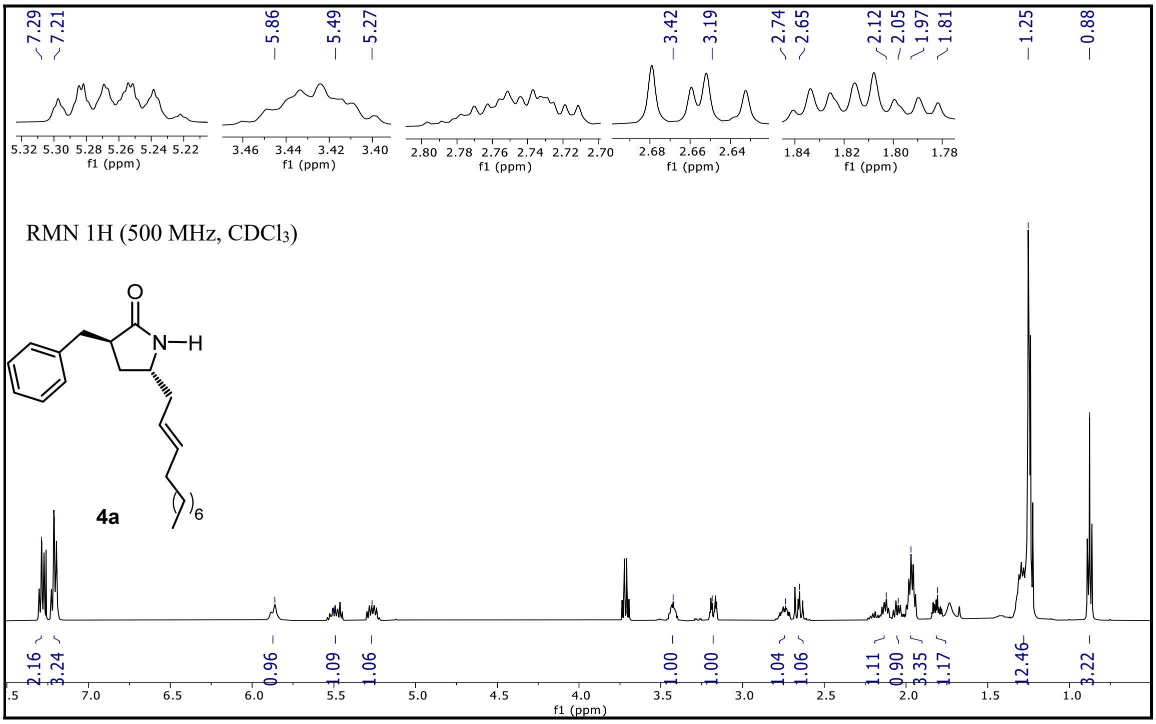

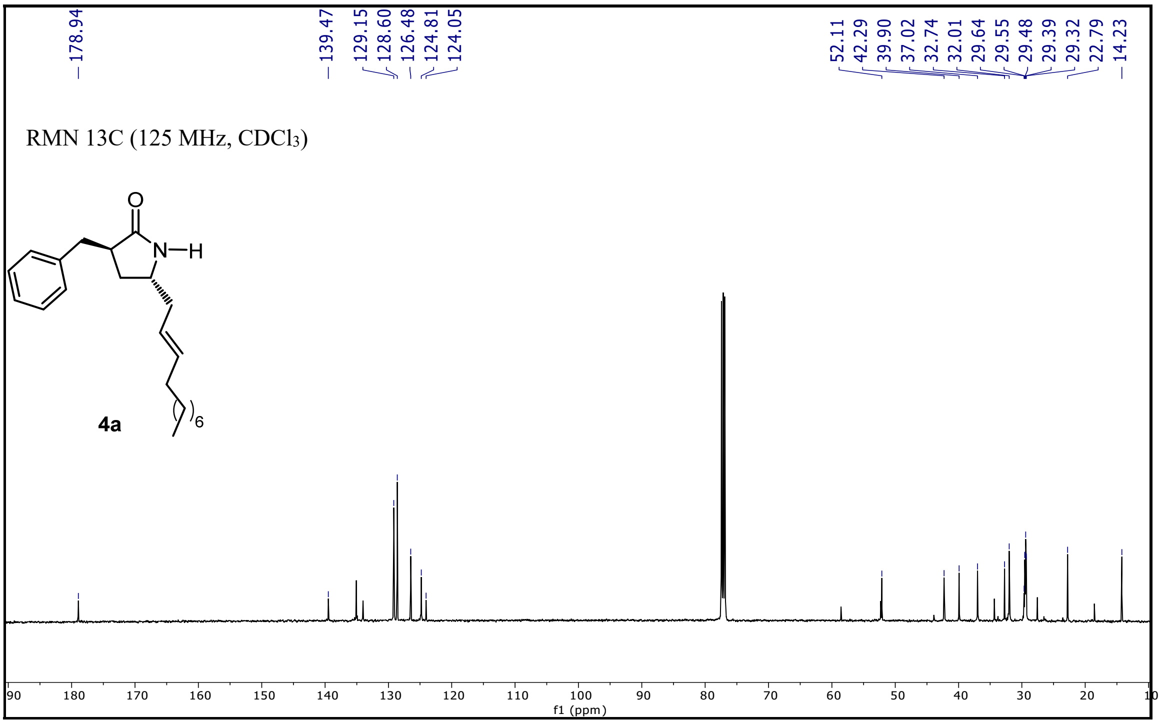

**(3*R*,5*R*)-3-(4-fluorobenzyl)-5-((*E*)-undec-2-en-1-yl)pyrrolidin-2-one (4b).**

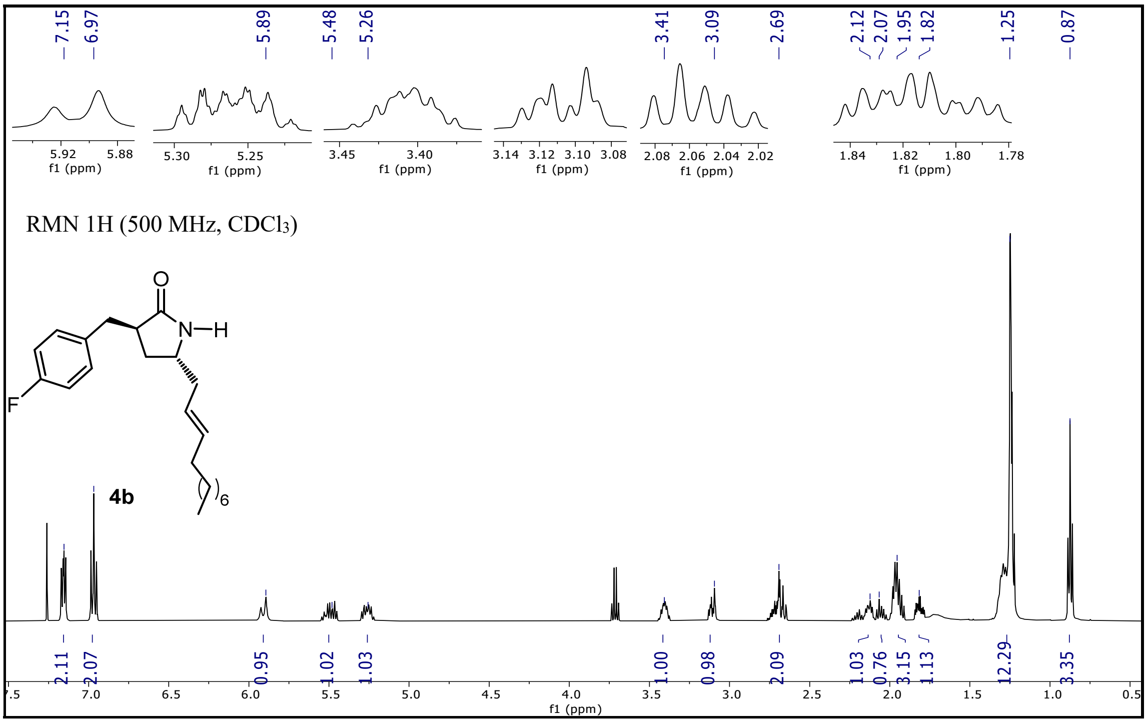


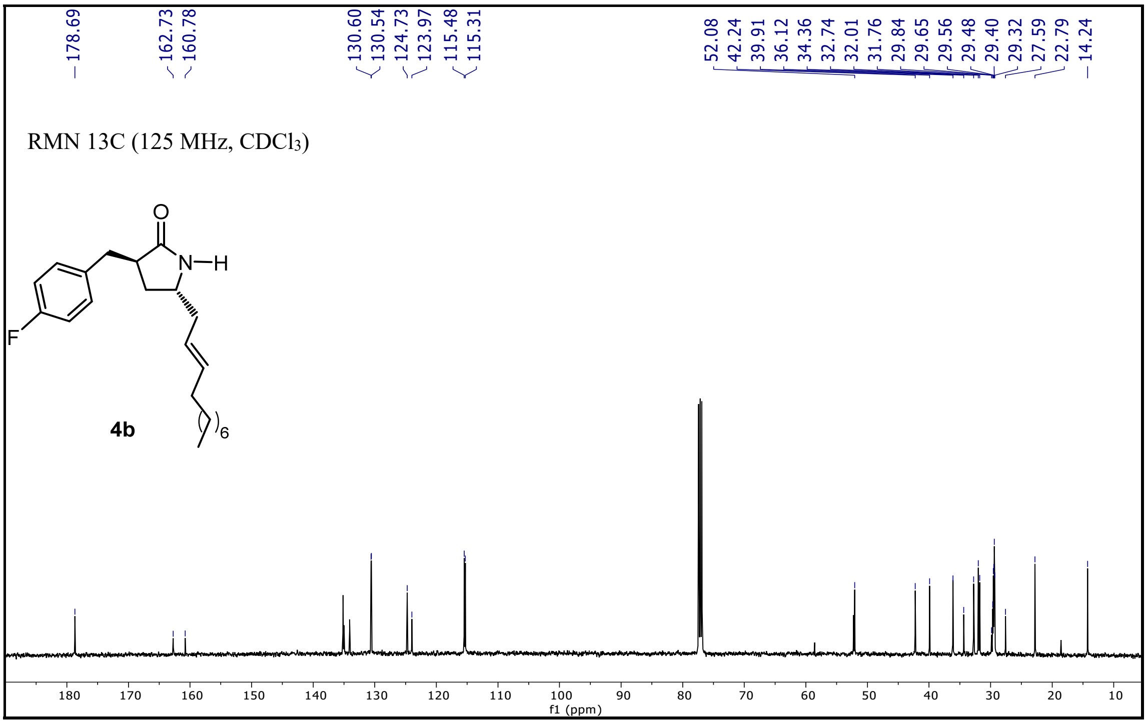


**(3*R*,5*R*)-3-benzyl-5-cinnamylpyrrolidin-2-one (4c).**

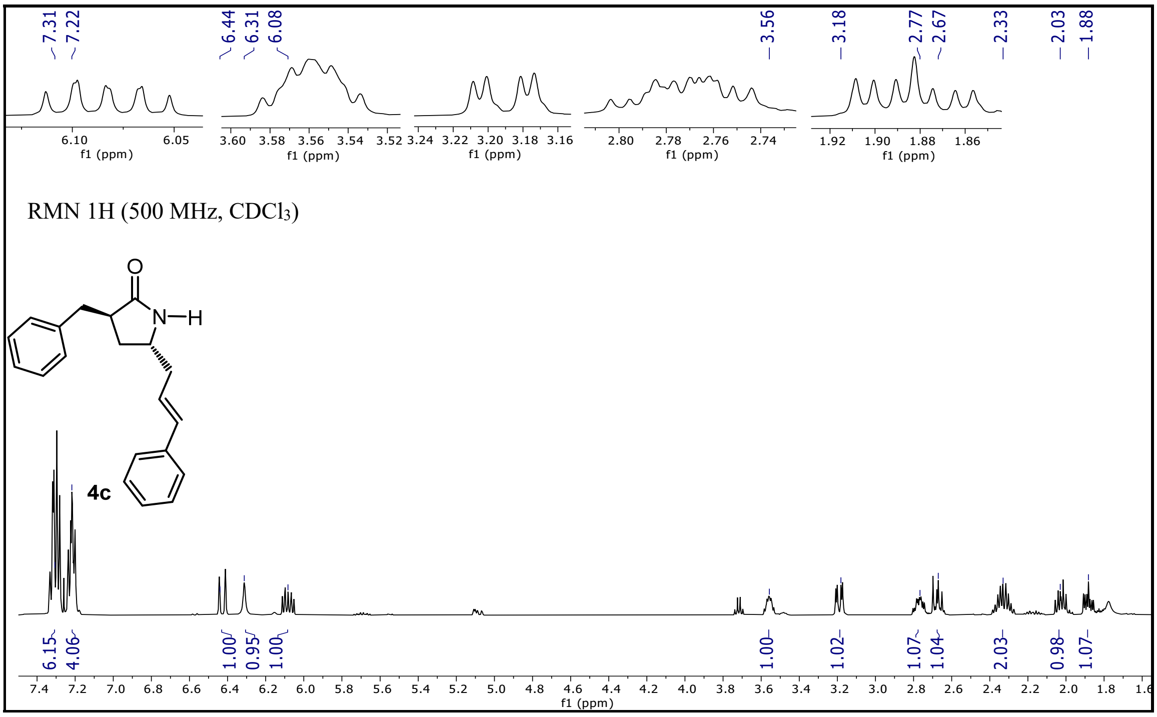


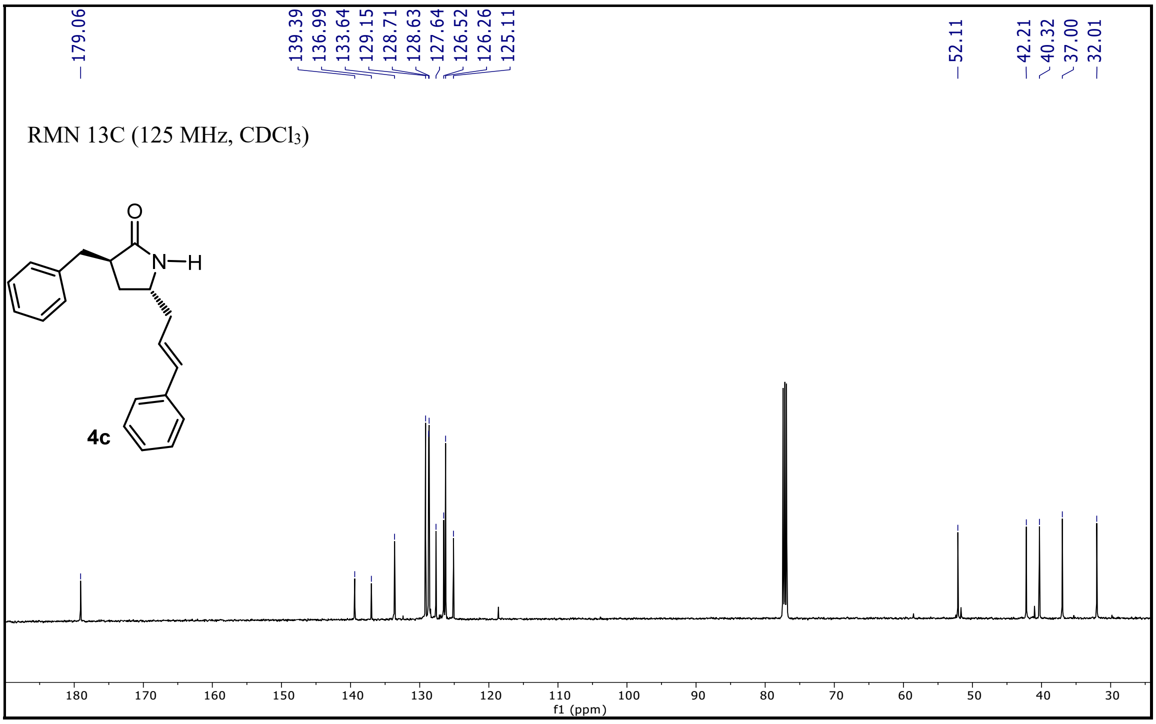


**(3*R*,5*R*)-5-cinnamyl-3-(4-fluorobenzyl)pyrrolidin-2-one (4d).**

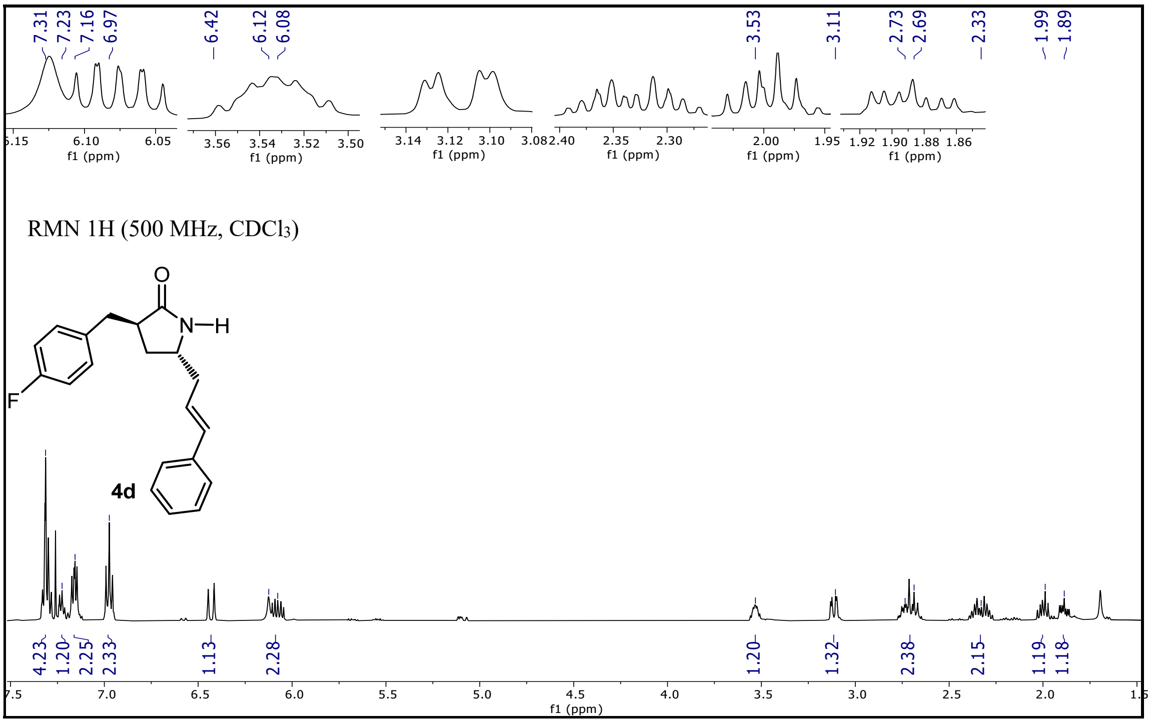


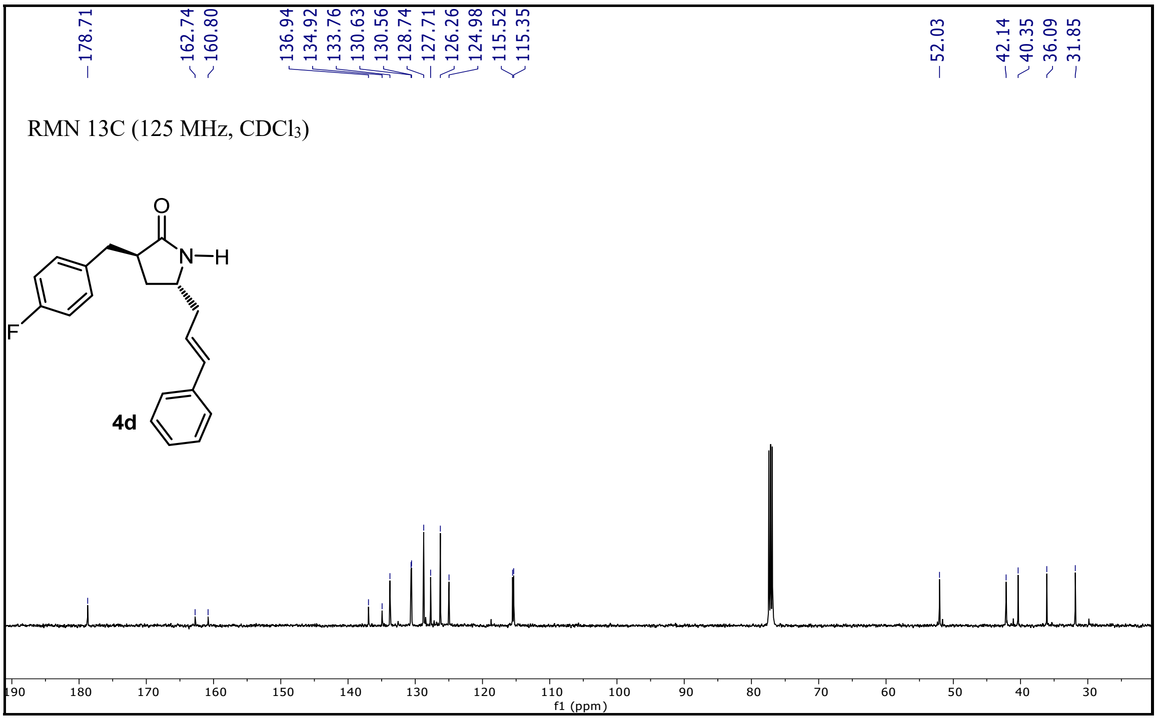


**(3*R*,5*R*)-3-benzyl-5-propylpyrrolidin-2-one (12a).**

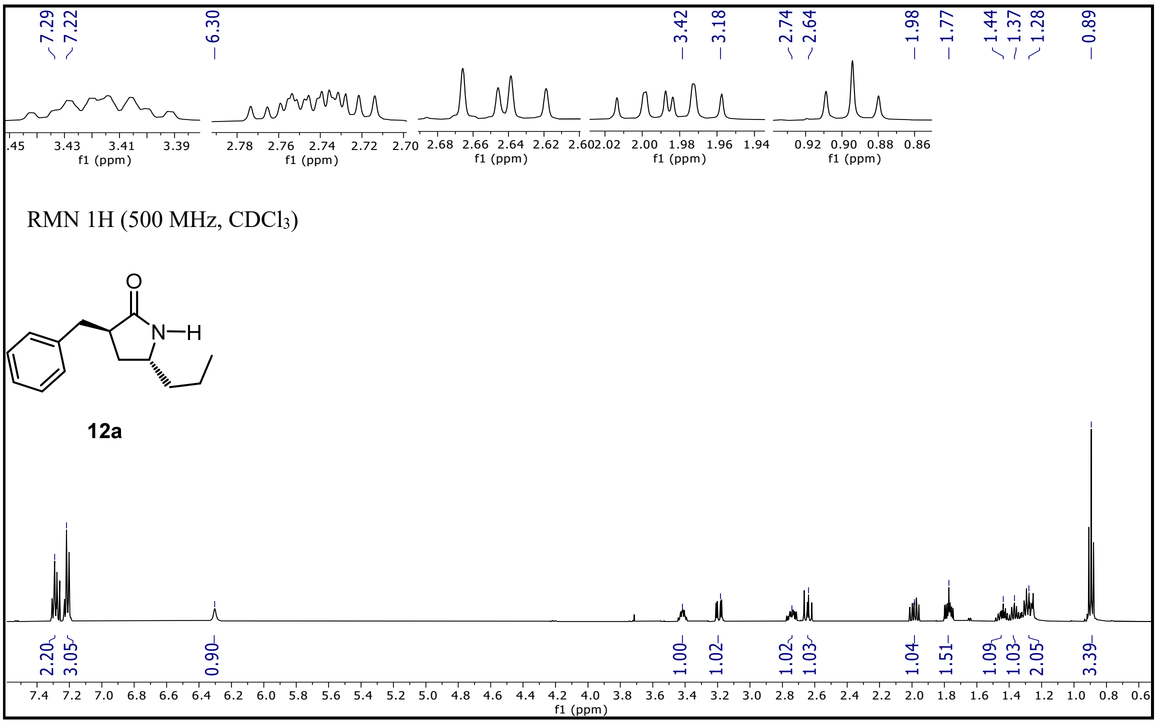

7.29
7.22
6.30
3.42
3.18
2.74
2.64
1.98
1.77
1.44
1.37
1.28
0.89
RMN 1H (500 MHz, CDCl3)
N–H
O
12a
2.20
3.05
0.90
1.00
1.02
1.02
1.03
1.04
1.51
1.09
1.03
2.05
3.39
f1 (ppm)


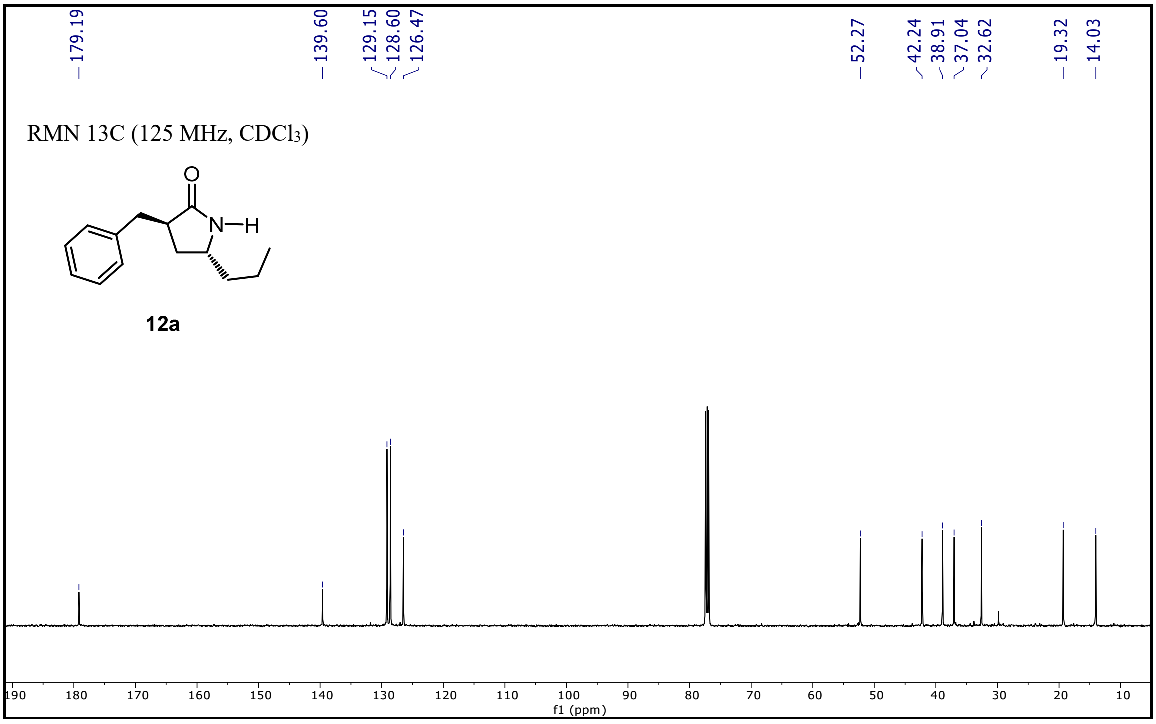

179.19
139.60
129.15
128.60
126.47
52.27
42.24
38.91
37.04
32.62
19.32
14.03
RMN 13C (125 MHz, CDCl3)
O
N–H
12a
f1 (ppm)

**(3*R*,5*R*)-3-(4-fluorobenzyl)-5-propylpyrrolidin-2-one (12b).**

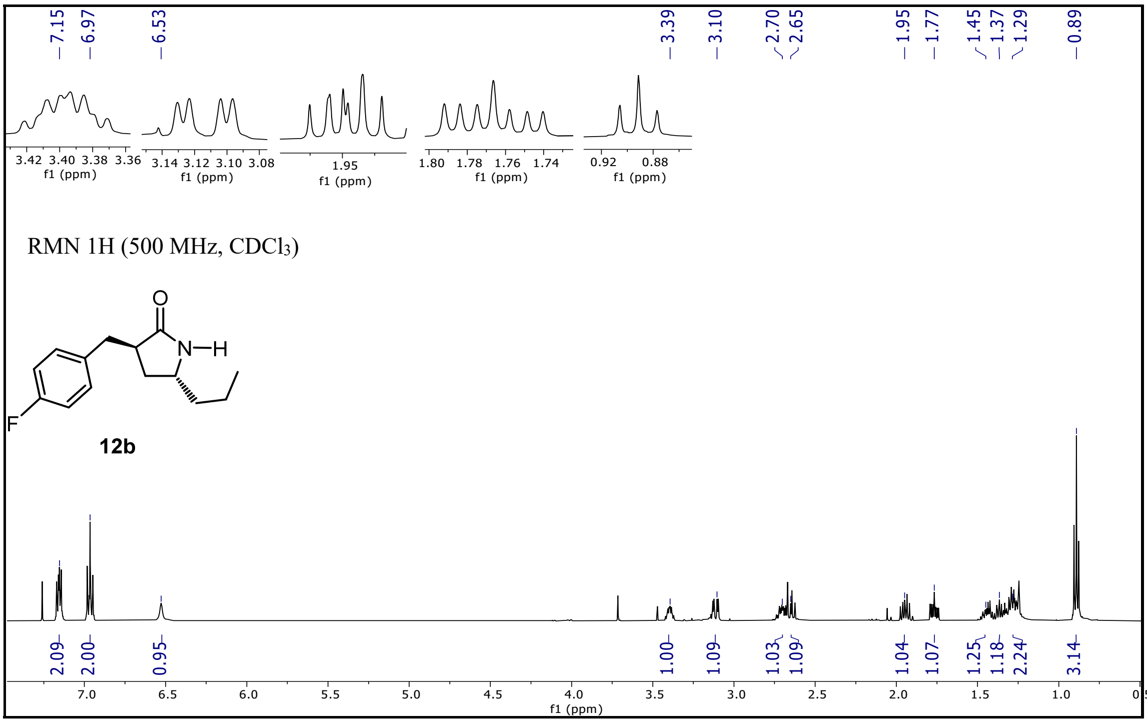


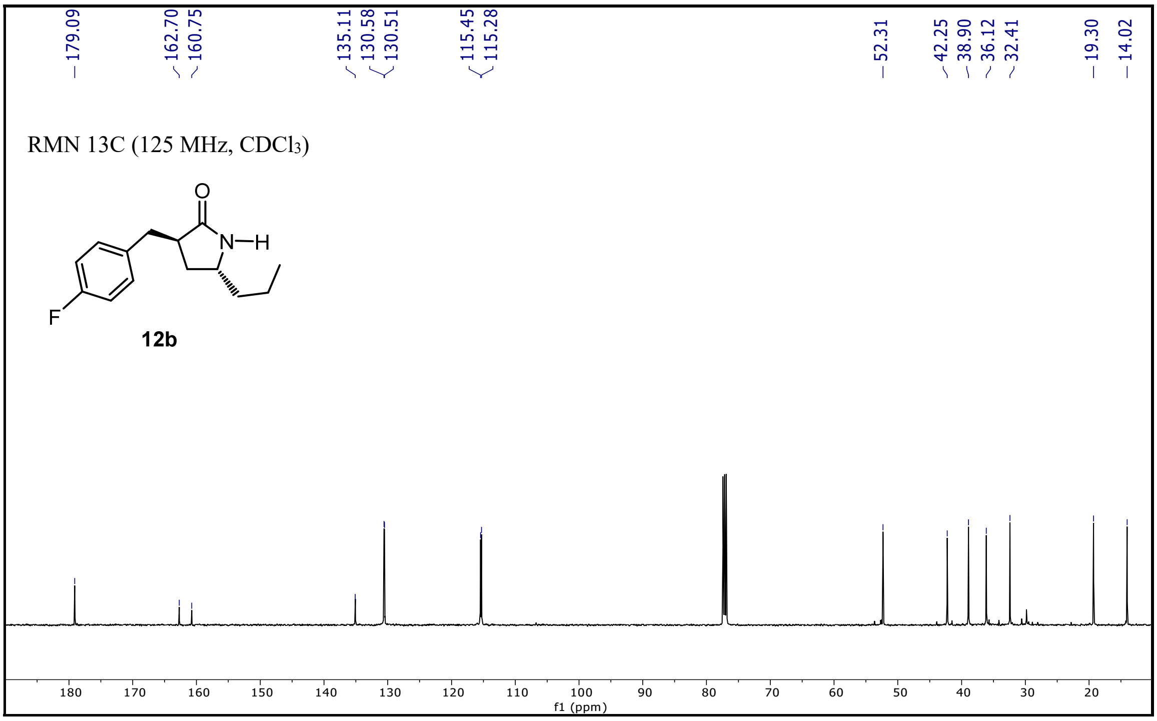

**(3*R*,5*R*)-3-benzyl-5-undecylpyrrolidin-2-one (13a).**

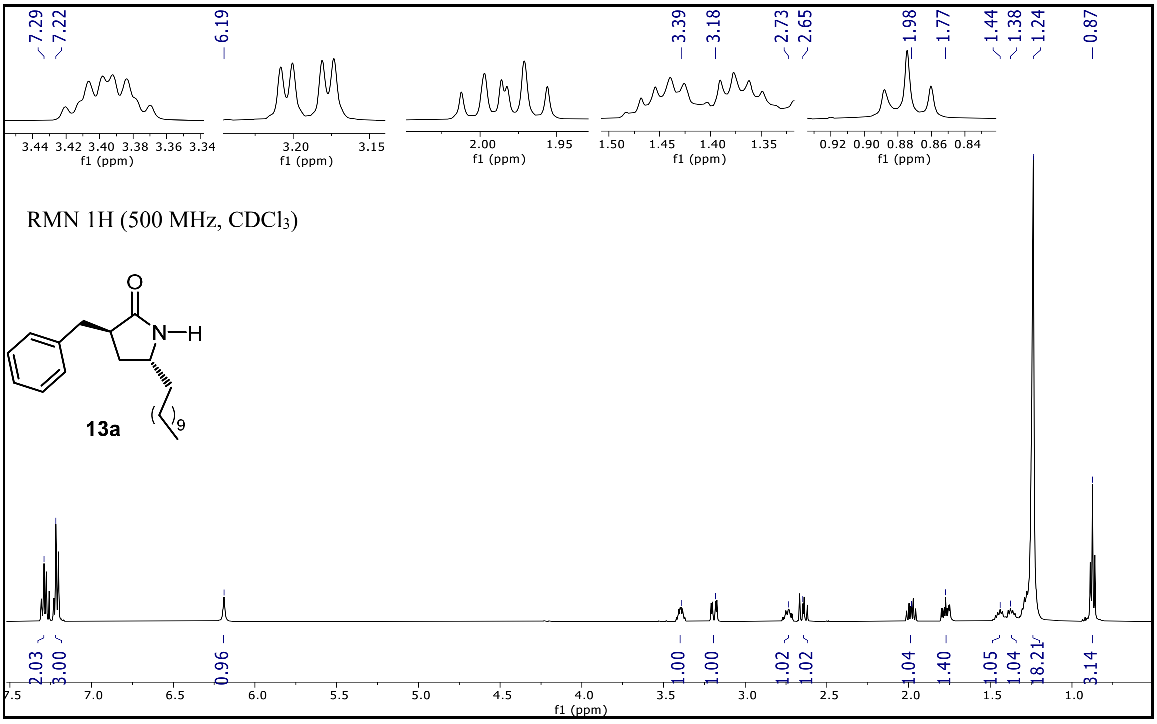


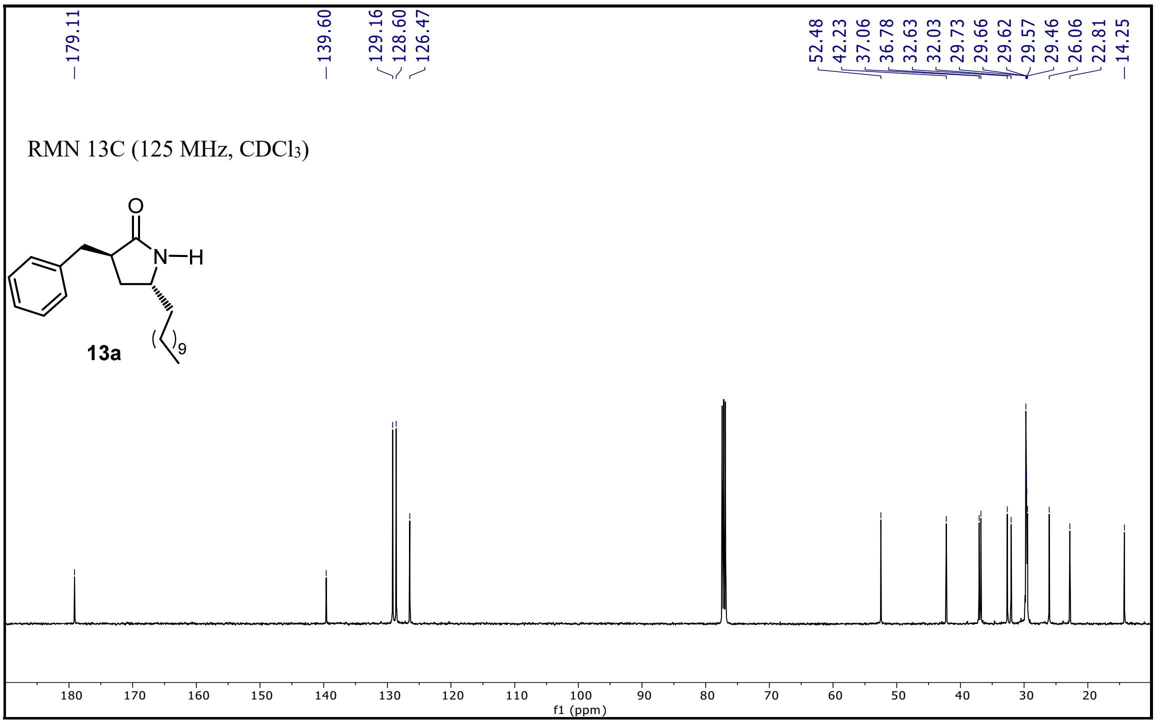

**(3*R*,5*R*)-3-(4-fluorobenzyl)-5-undecylpyrrolidin-2-one (13b).**

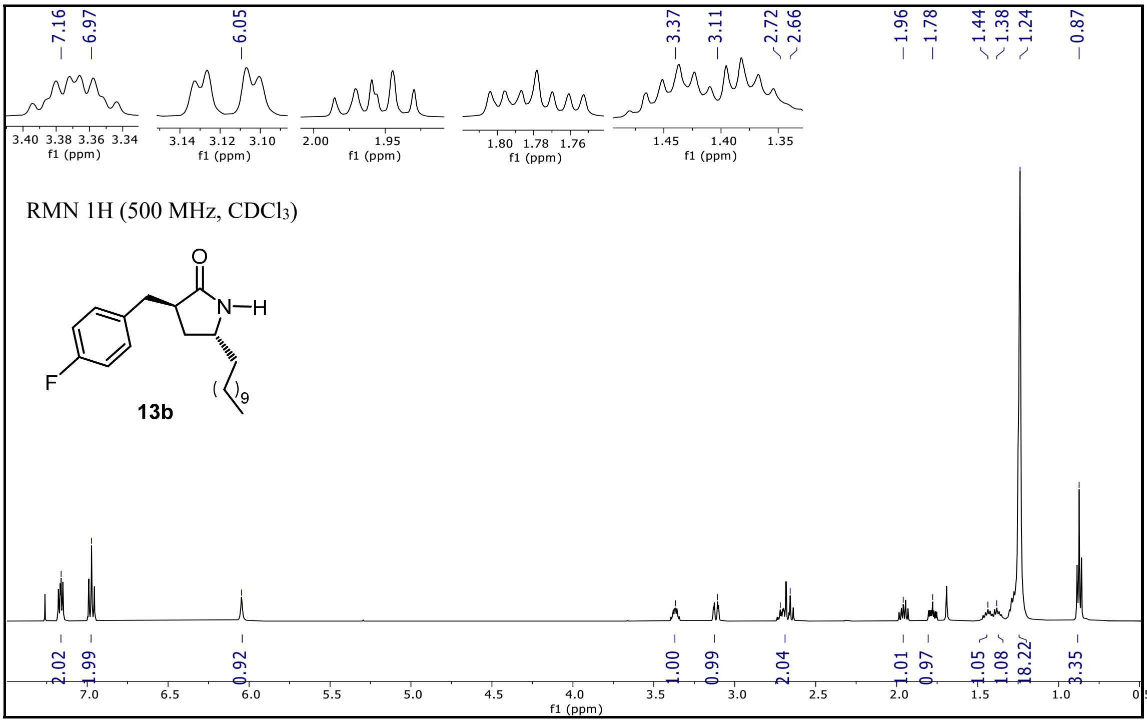


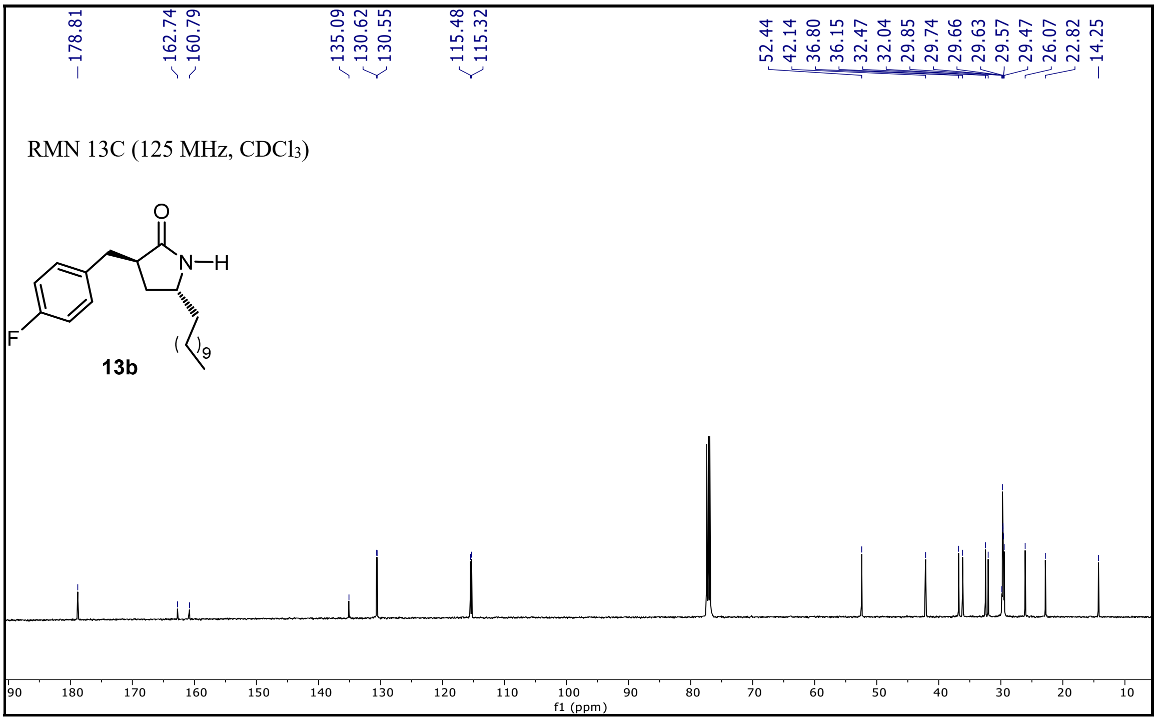

**(3*R*,5*R*)-3-benzyl-5-(3-phenylpropyl)pyrrolidin-2-one (13c).**

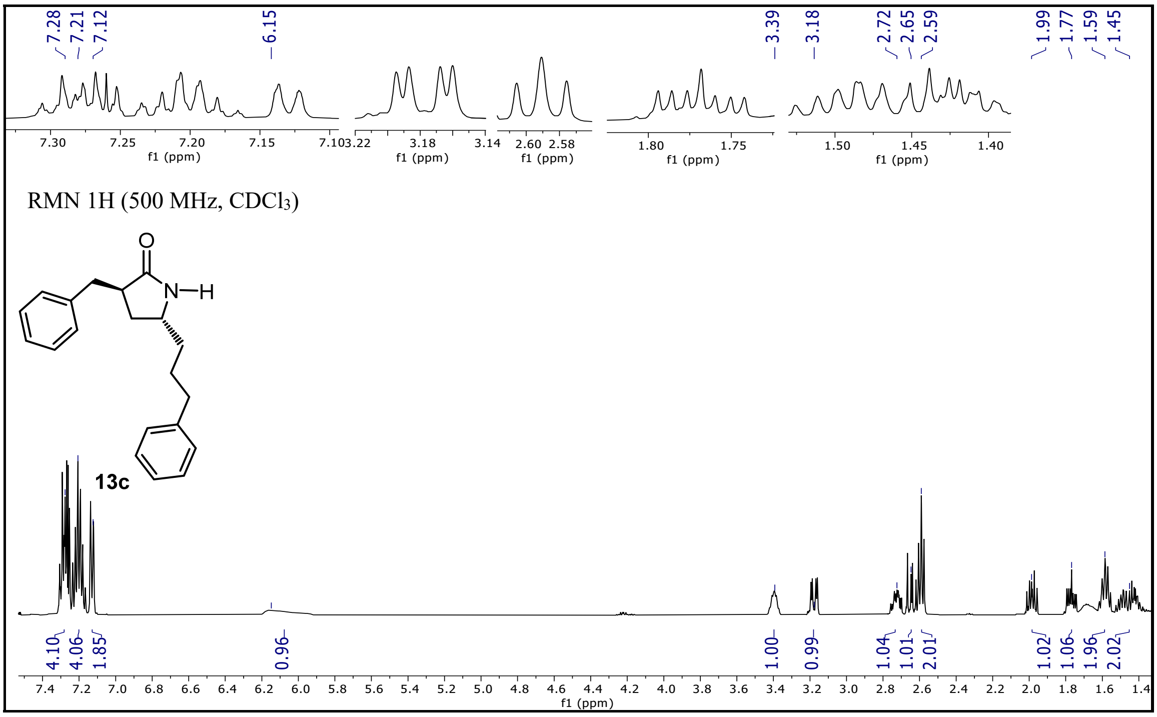


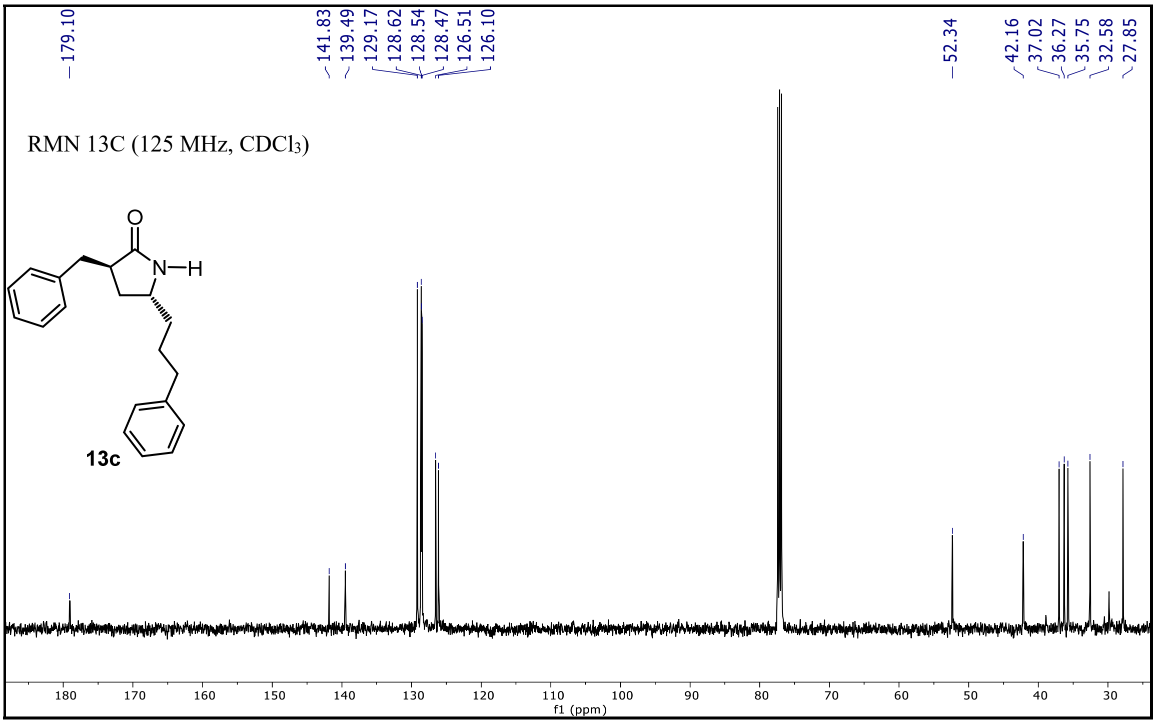

**(3*R*,5*R*)-3-(4-fluorobenzyl)-5-(3-phenylpropyl)pyrrolidin-2-one (13d).**

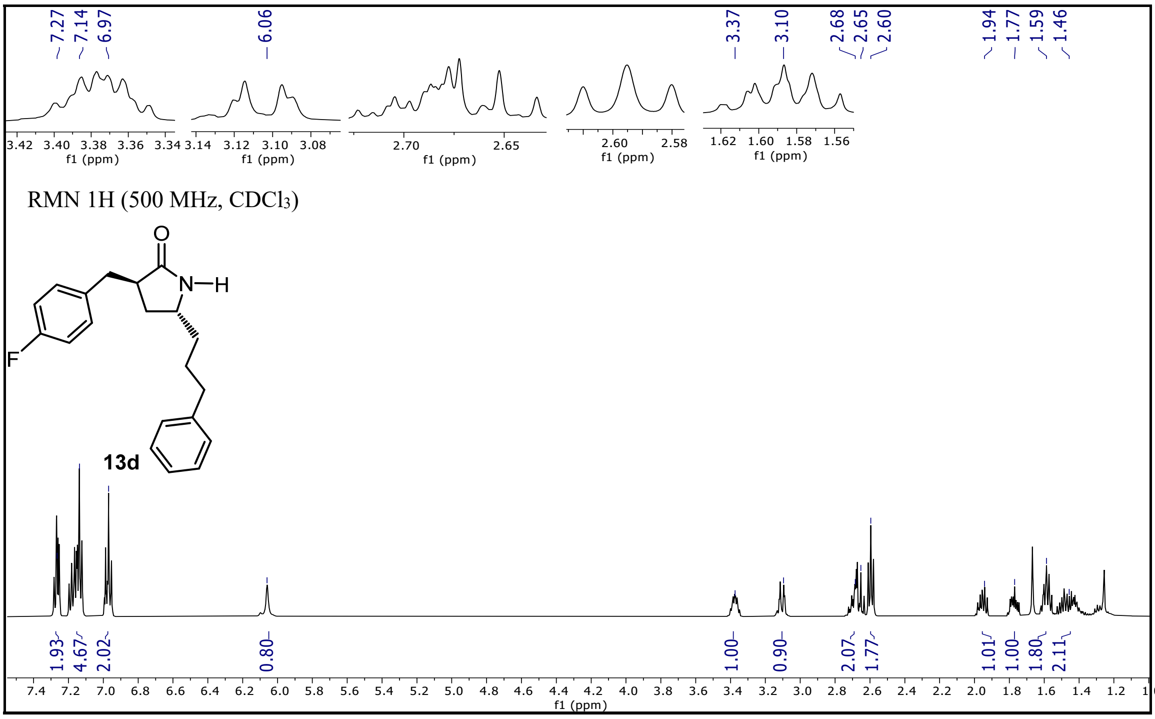


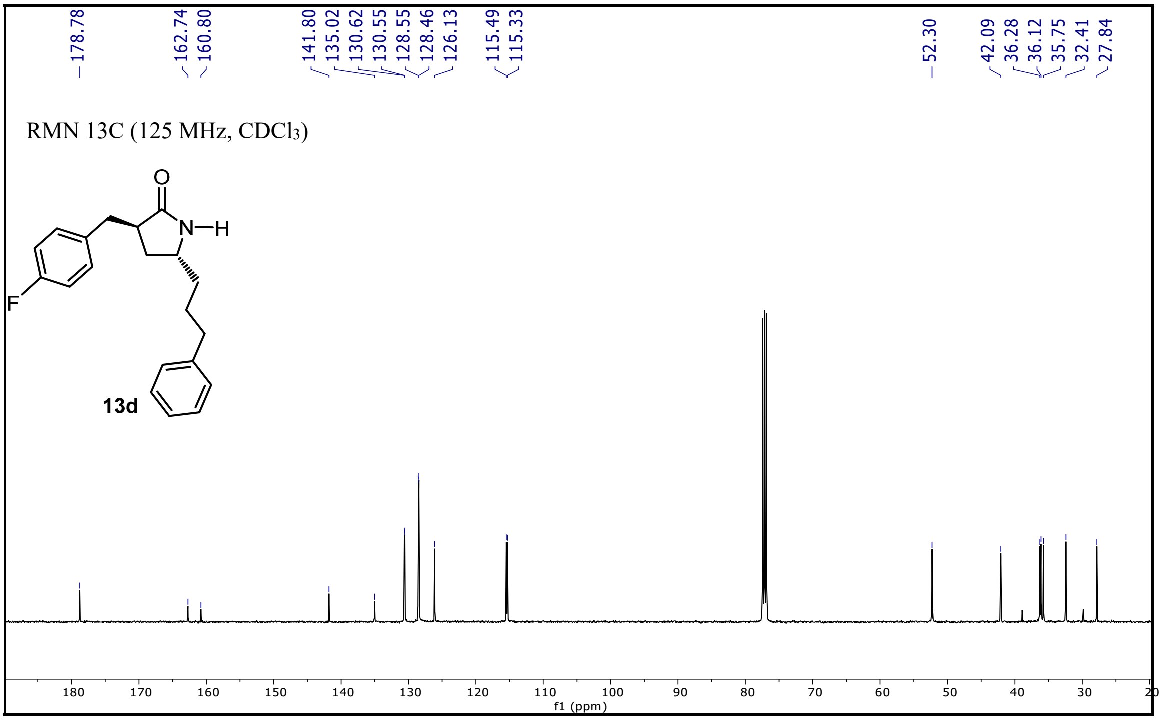

**(3*R*,5*R*)-1,5-diallyl-3-benzylpyrrolidin-2-one (14).**

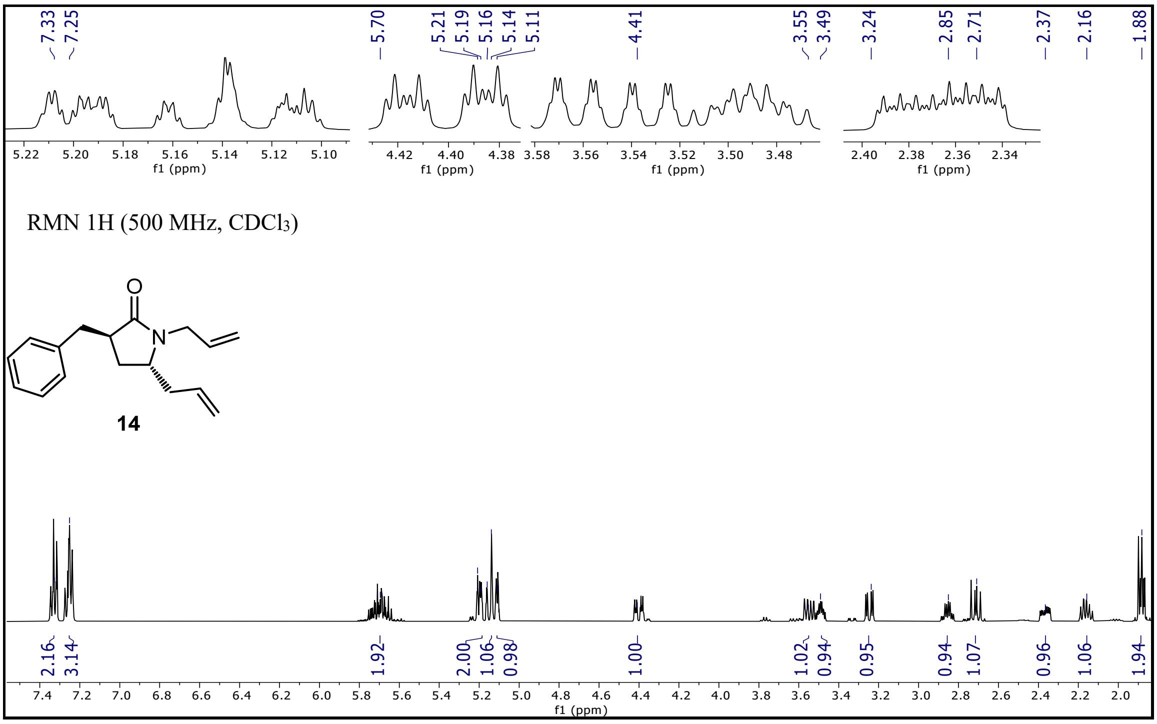


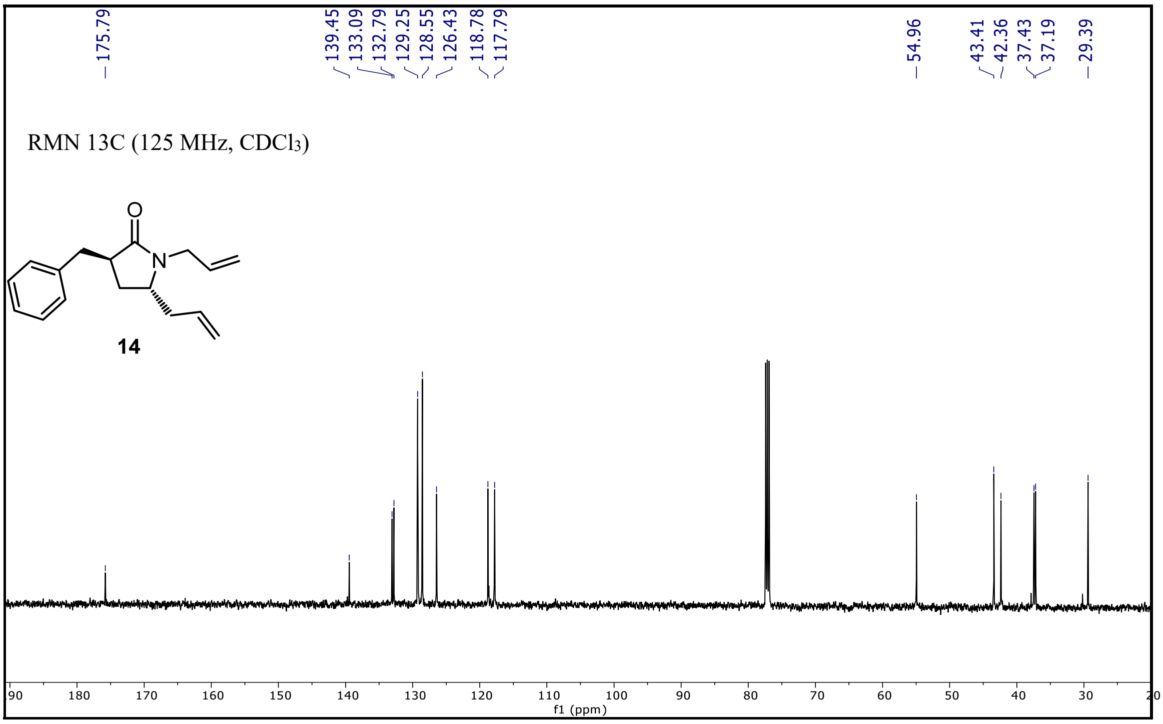

**(2*R*,8a*R*)-2-benzyl-1,5,8,8a-tetrahydroindolizin-3(2*H*)-one (15).**

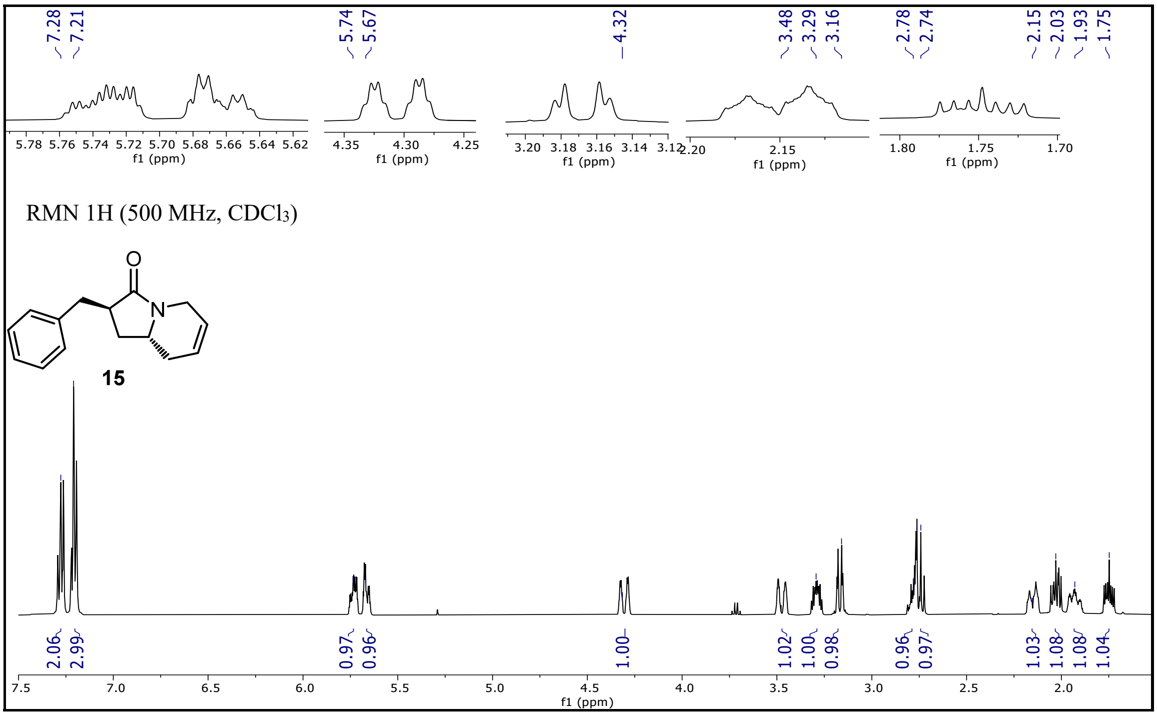


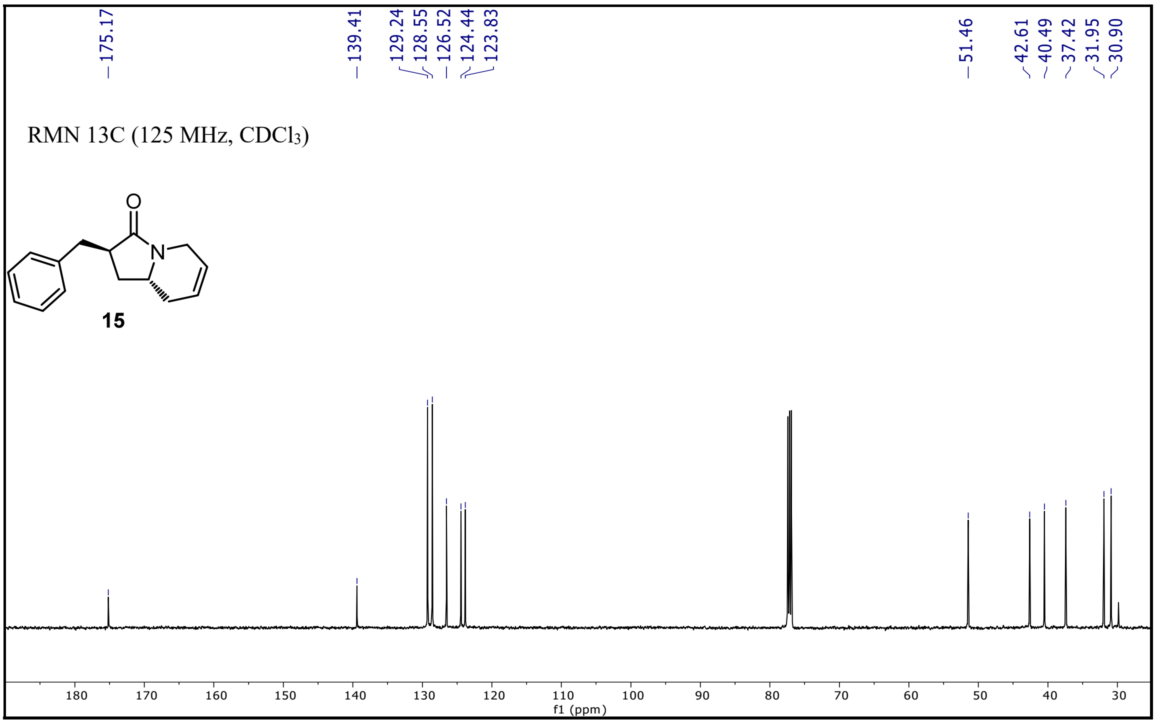

**(2*R*,8a*R*)-2-benzylhexahydroindolizin-3(2*H*)-one (18).**

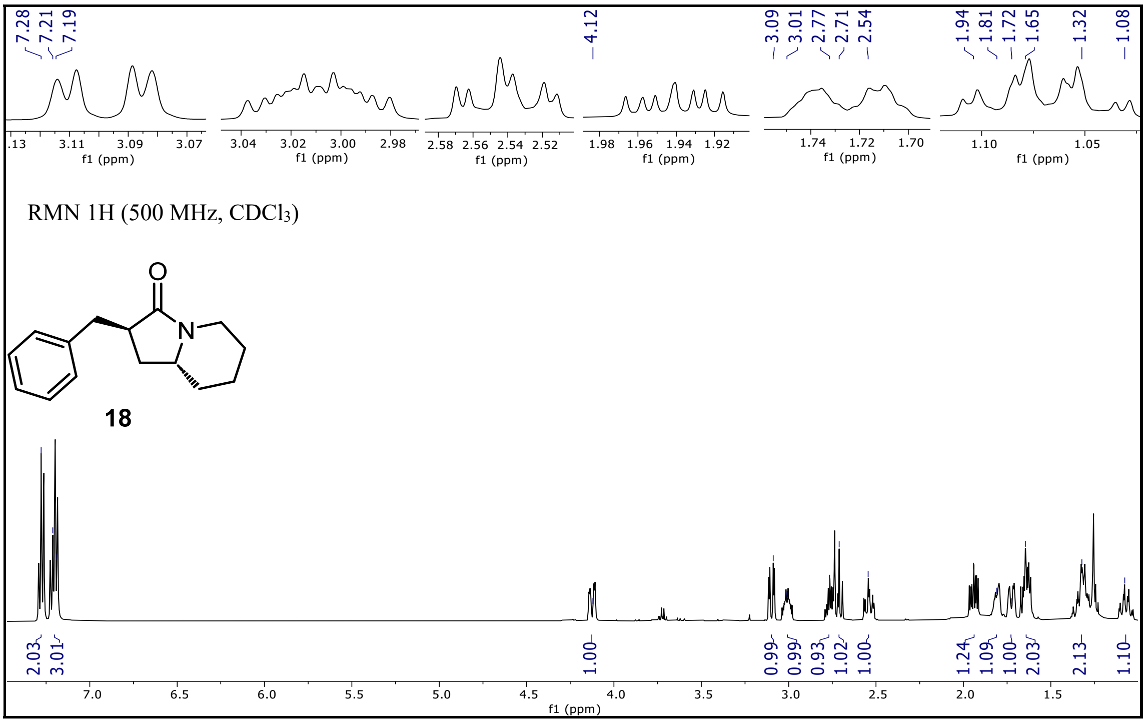


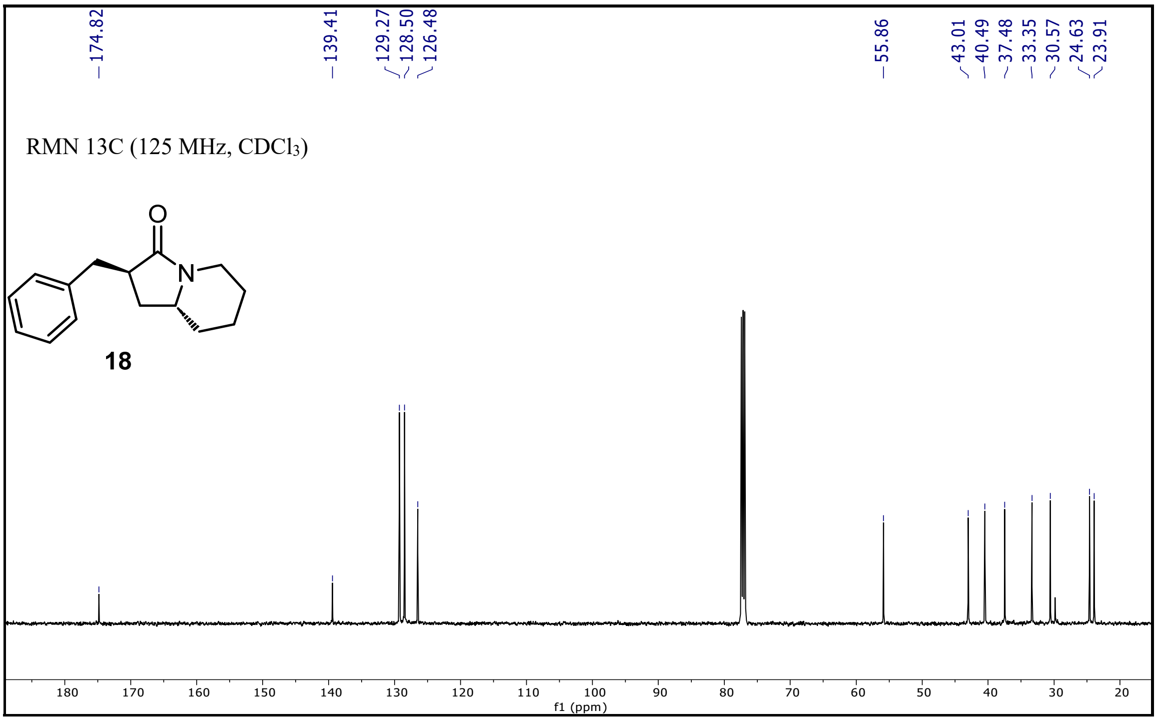

**(2*R*,4*R*)-4-amino-2-benzylheptanoic acid hydrochloride (5a).**

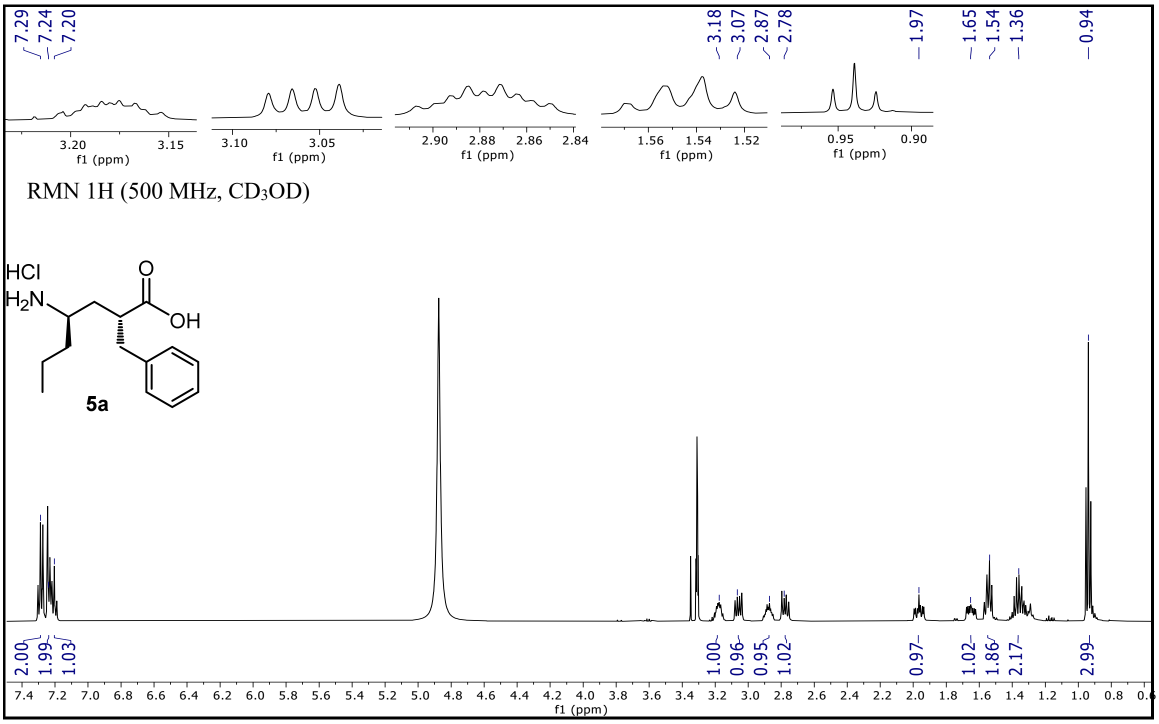


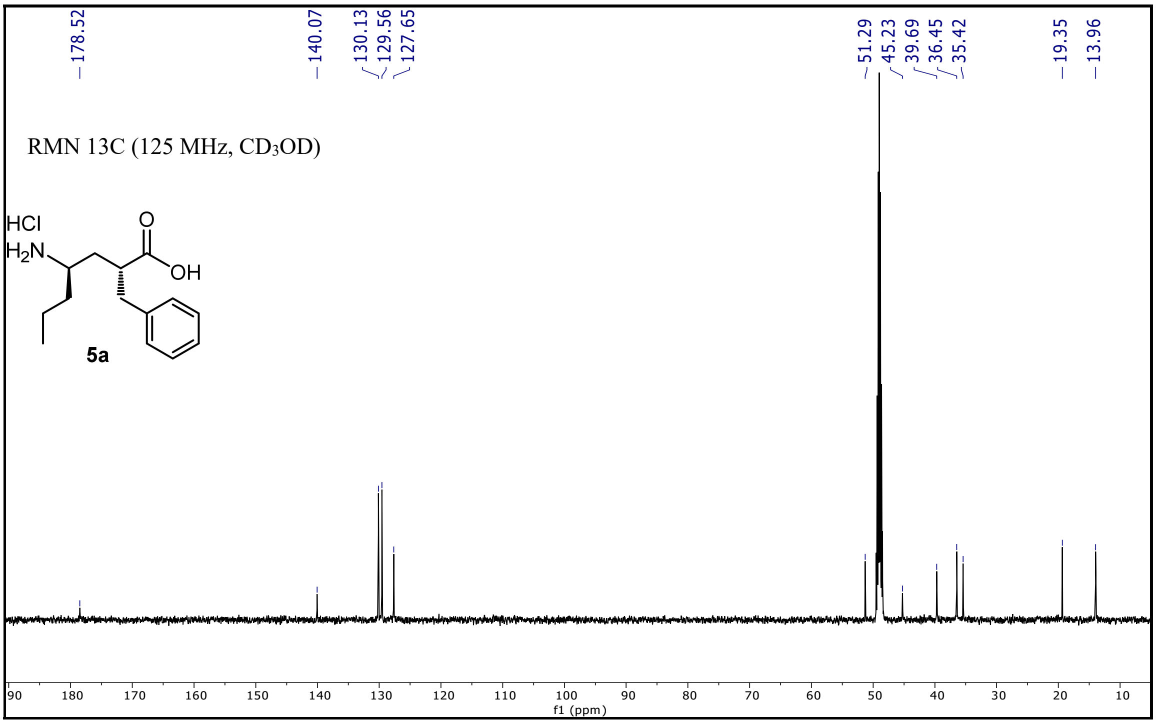

**(2R,4R)-4-amino-2-(4-fluorobenzyl)heptanoic acid hydrochloride 5b.**

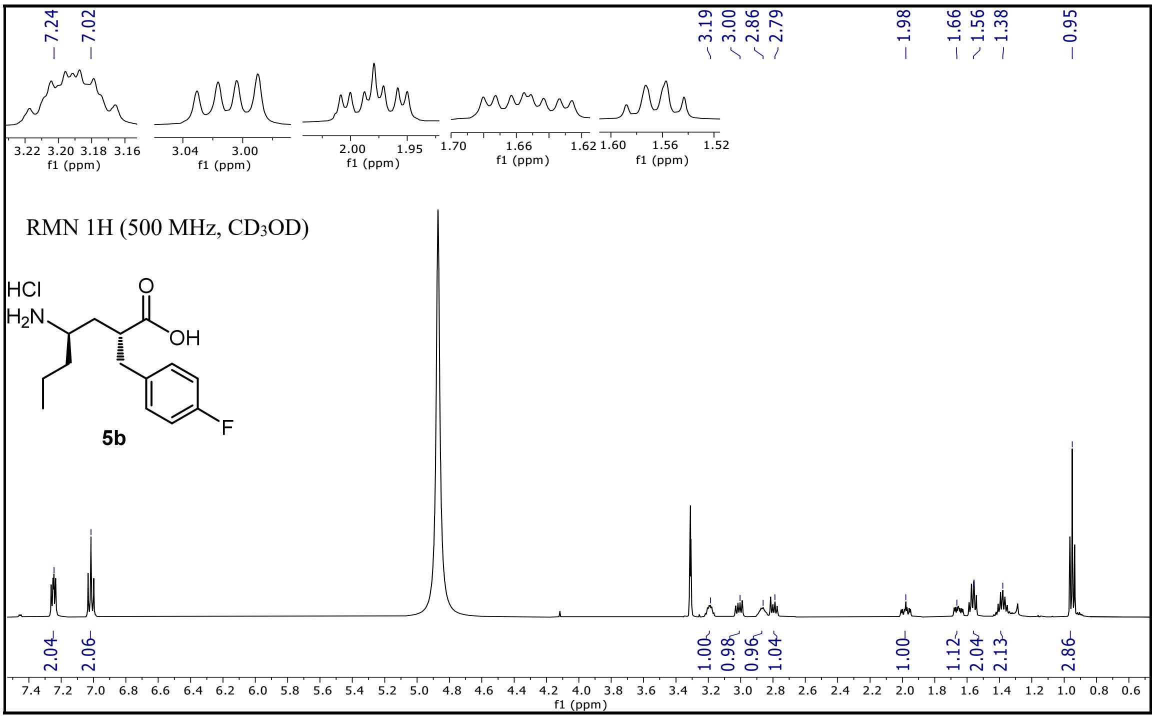


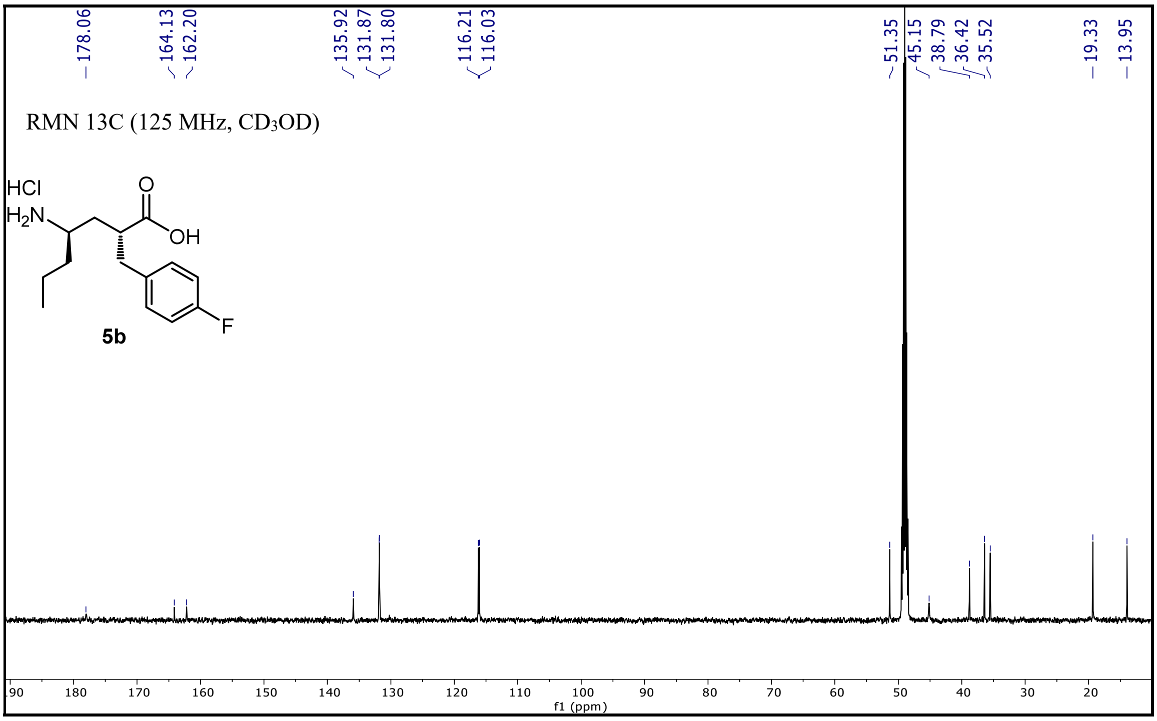

**(2*R*,4*R*)-4-amino-2-benzylpentadecanoic acid hydrochloride (5c).**

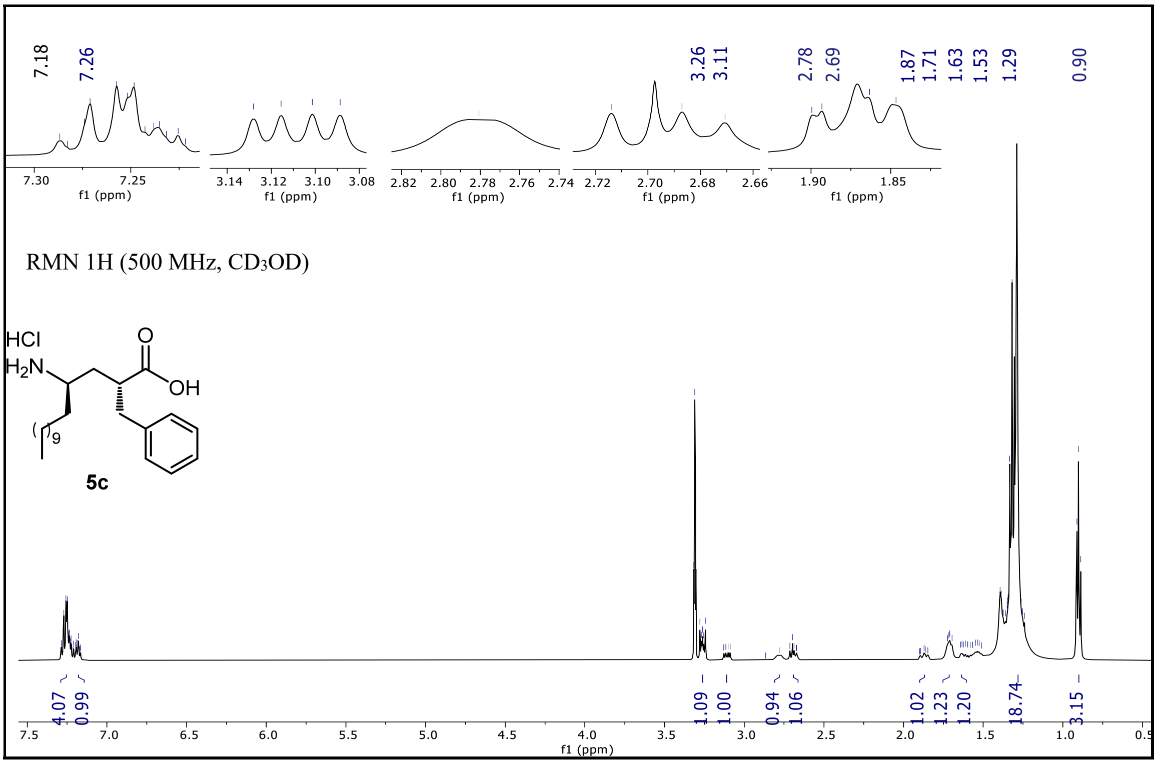


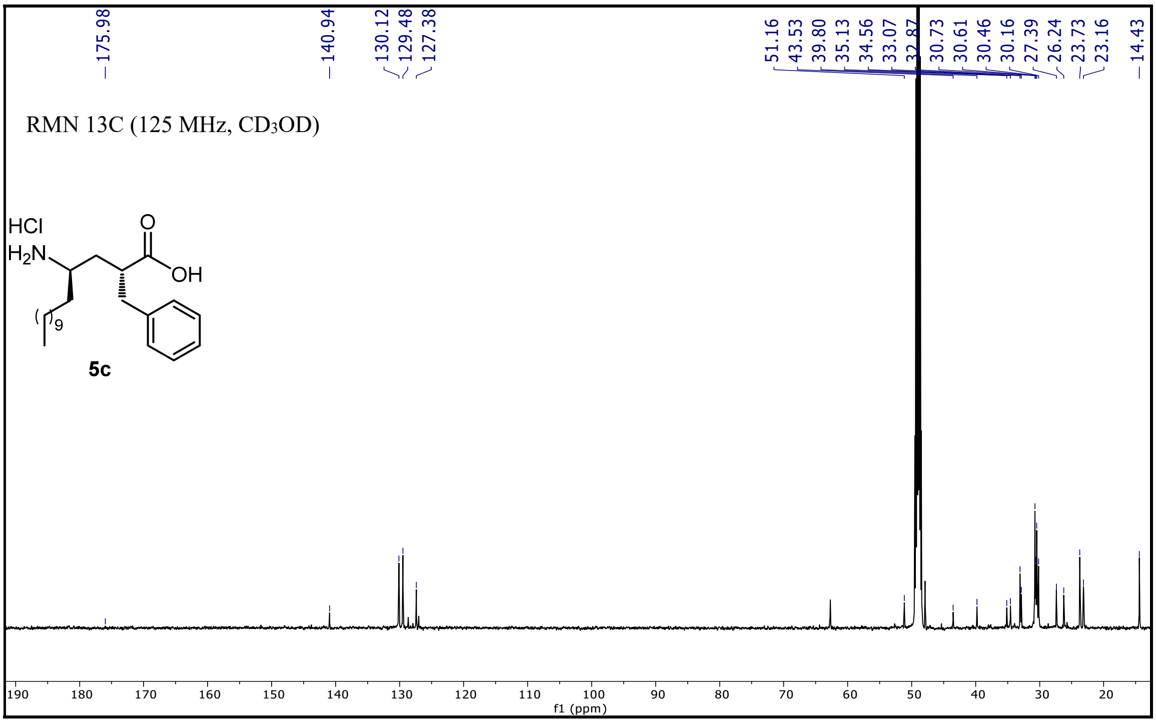

**(2*R*,4*R*)-4-amino-2-(4-fluorobenzyl)pentadecanoic acid hydrochloride (5d).**

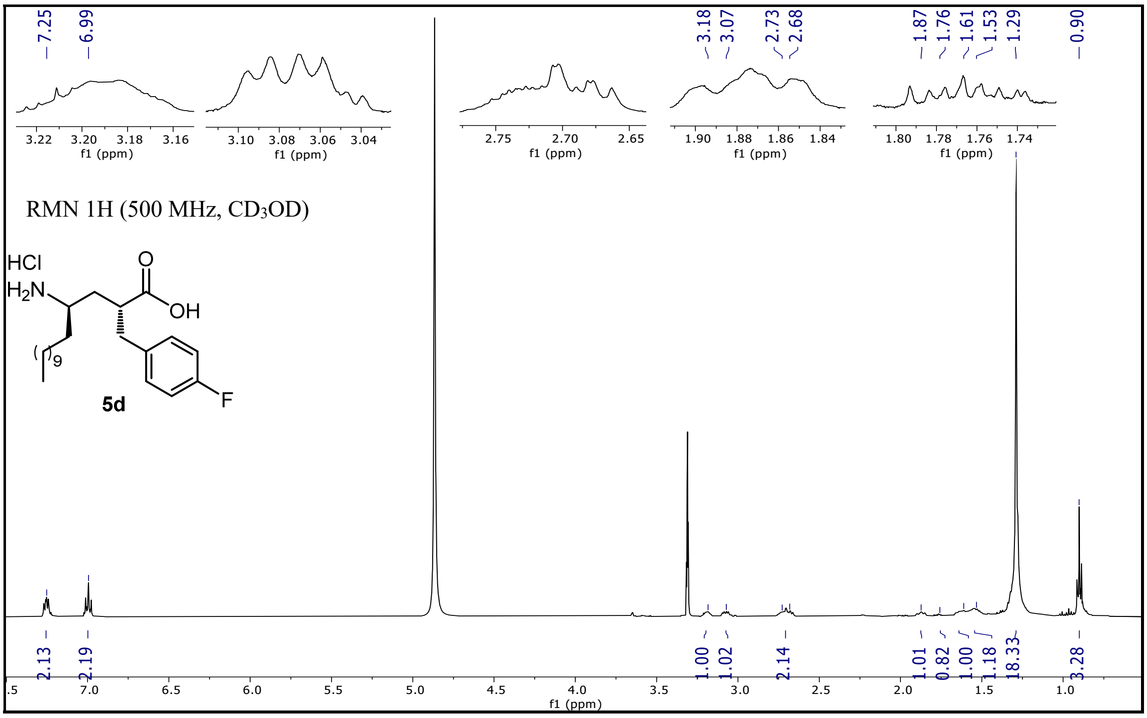


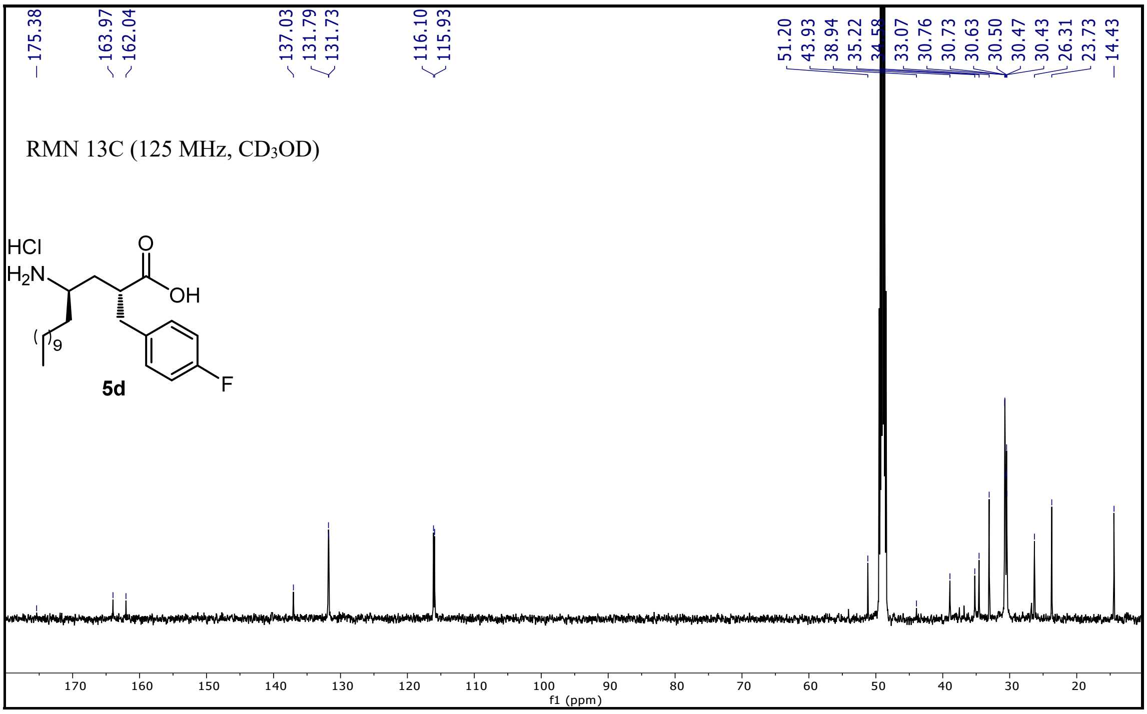

**(2*R*,4*R*)-4-amino-2-benzyl-7-phenylheptanoic acid hydrochloride (5e).**

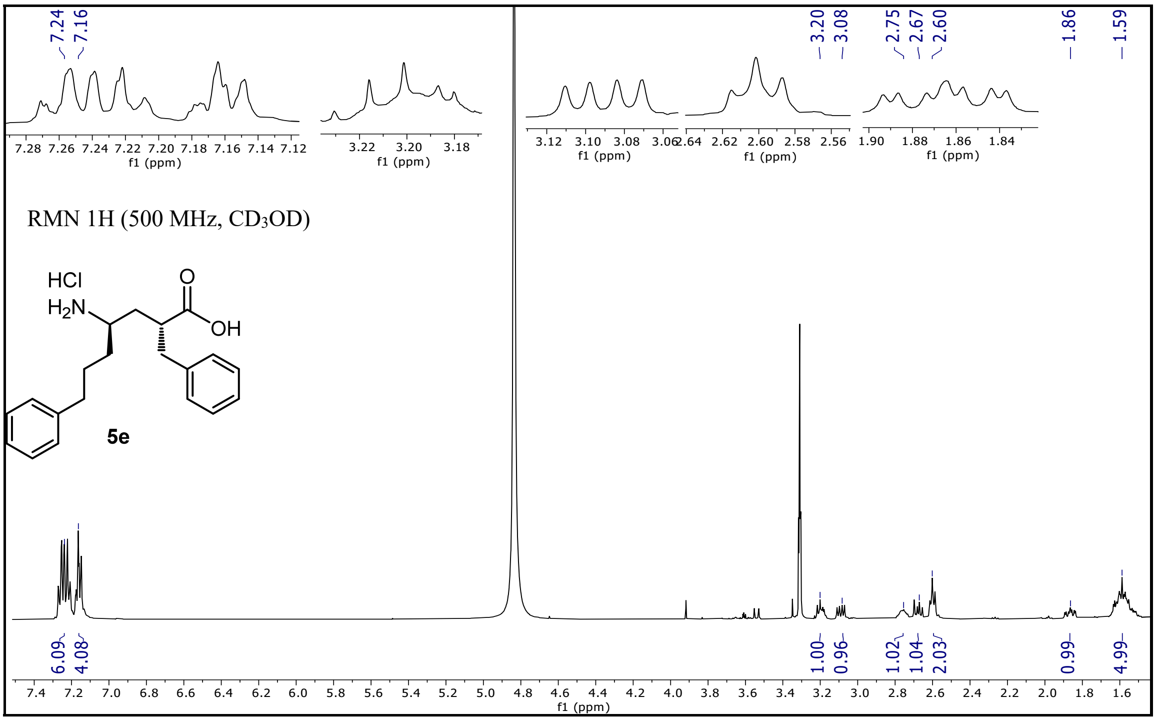


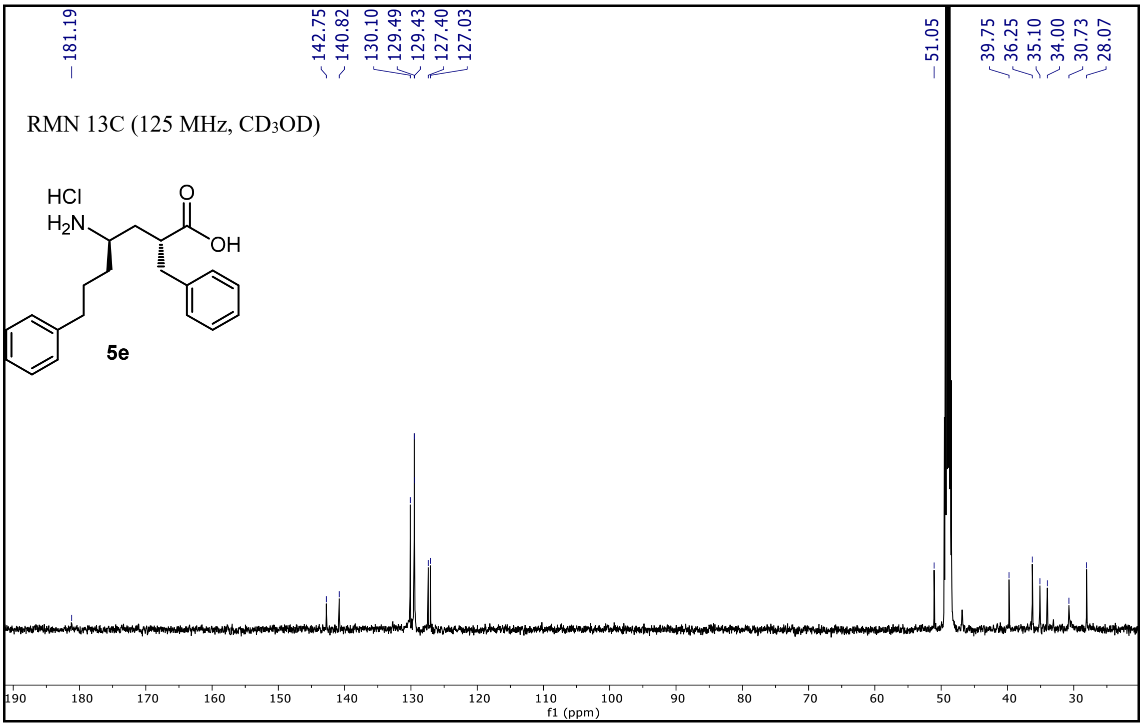

**(2*R*,4*R*)-4-amino-2-(4-fluorobenzyl)-7-phenylheptanoic acid hydrochloride (5f).**

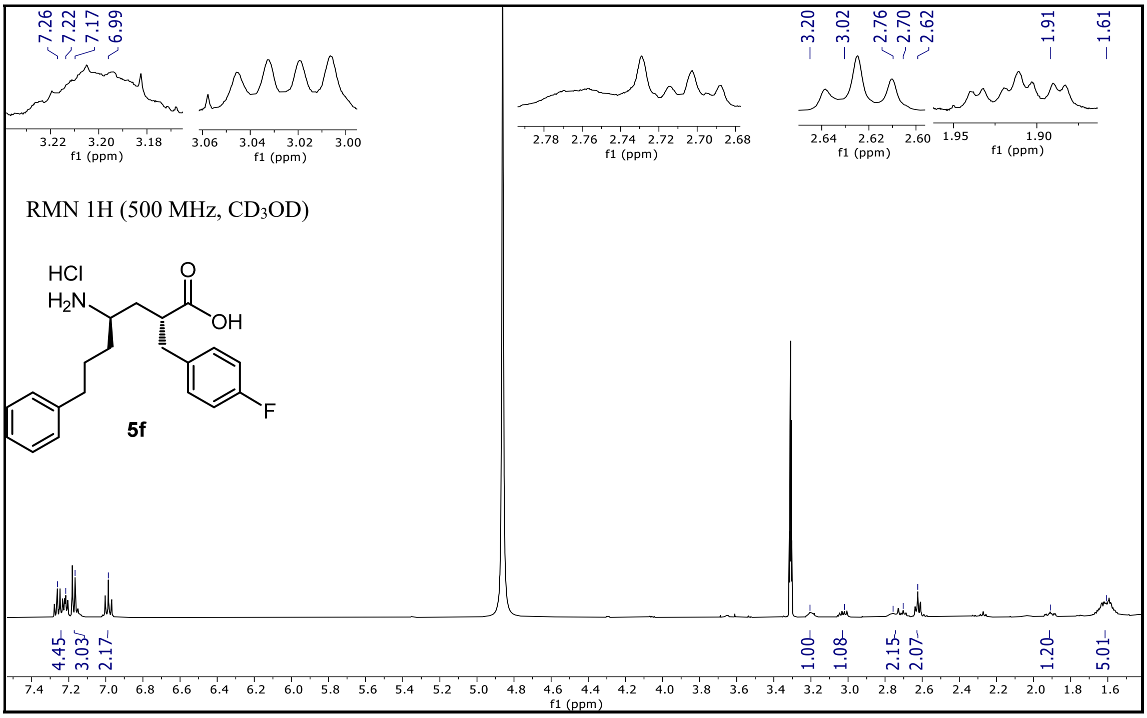


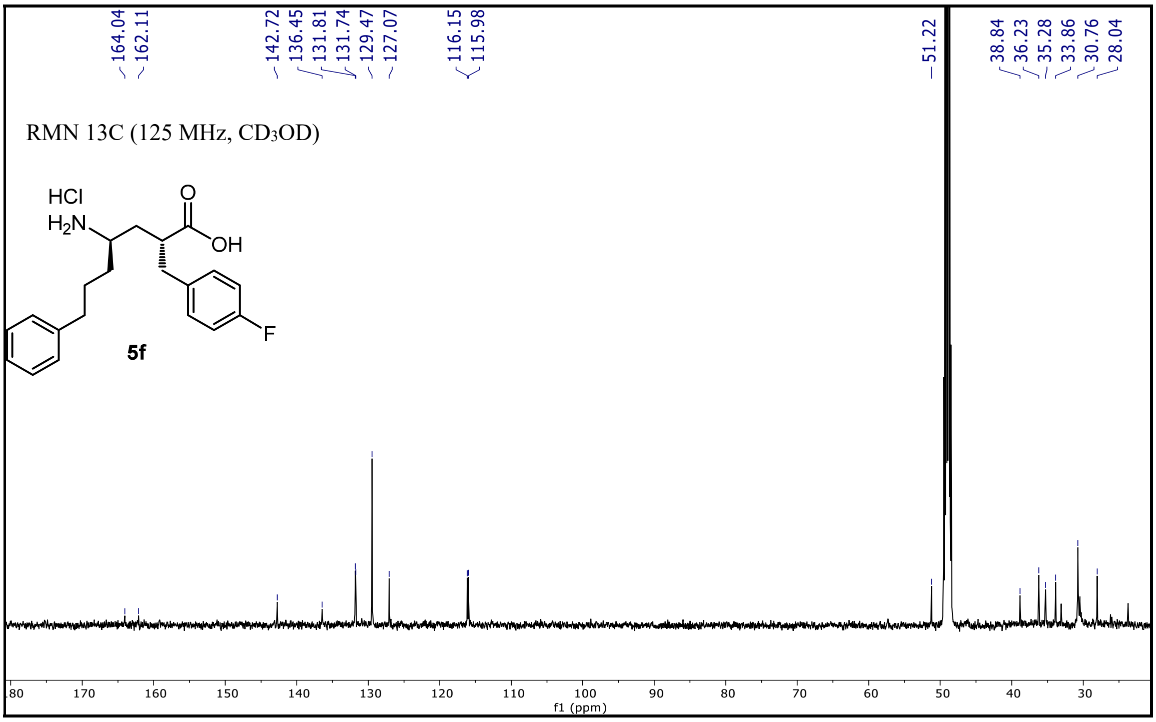

**(*R*)-2-benzyl-3-((*R*)-piperidin-2-yl)propanoic acid hydrochloride (6).**

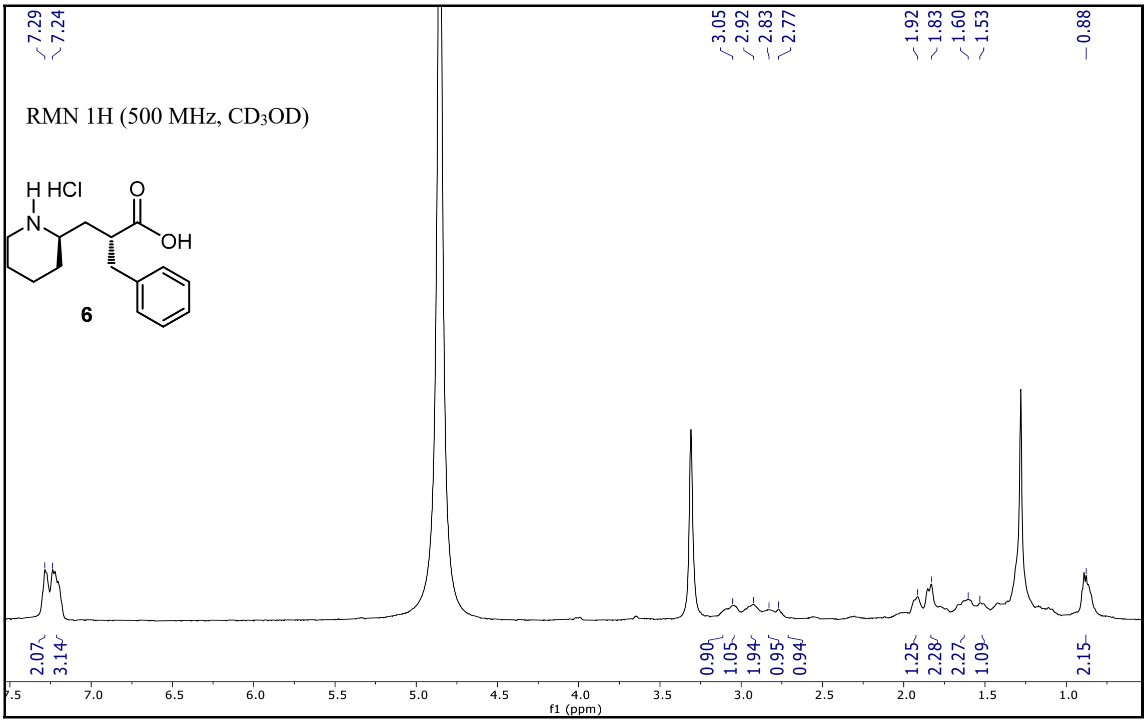


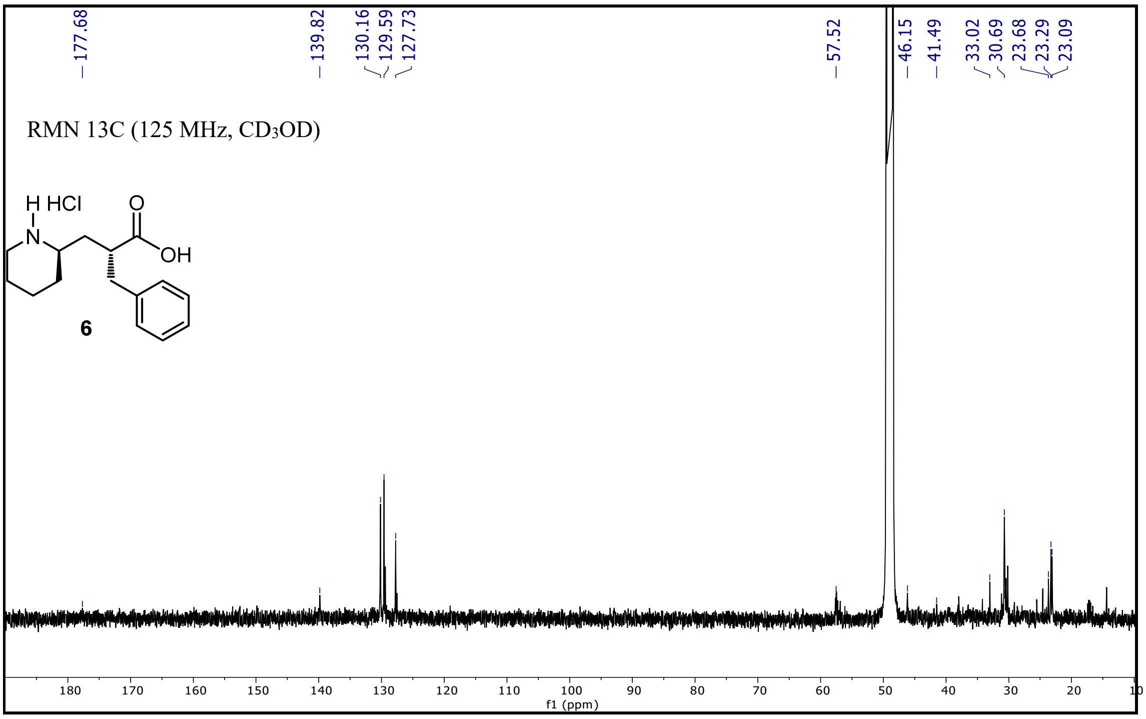

Supplement: Supplementary file 1 [file molecules-31-02087-s001.zip › molecules-4367261-supplementary.pdf]
